# mRNA-based tuberculosis vaccines BNT164a1 and BNT164b1 are immunogenic, well tolerated and efficacious in rodent models

**DOI:** 10.1038/s41590-026-02545-z

**Published:** 2026-06-12

**Authors:** Neha Agrawal, Louis S. Ates, Stefan A. Schille, Anuhar Chaturvedi, Janina Vogt, Natasa Vukovic, Charles L. Dulberger, Annette B. Vogel, Jan Diekmann, Mustafa Diken, Uğur Şahin

**Affiliations:** 1 BioNTech SE, Mainz, Germany; 2 BioNTech US, Cambridge, MA USA; 3 Translational Oncology at the University Medical Center of the Johannes Gutenberg-University Mainz (TRON), Mainz, Germany; 4 Helmholtz Institute for Translational Oncology (HI-TRON), Mainz, Germany

**Keywords:** Tuberculosis, RNA vaccines

## Abstract

We designed and preclinically tested two mRNA–lipid-nanoparticle-based vaccine candidates to protect against tuberculosis. BNT164a1 and BNT164b1 encode the same eight *Mycobacterium tuberculosis* antigens expressed across different infection stages: Ag85A, Hrp1, ESAT-6, RpfD, RpfA, HbhA, M72 and VapB47. BNT164a1 utilizes nucleoside-unmodified mRNA, whereas BNT164b1 utilizes *N*^1^-methylpseudouridine-modified mRNA. Prime-boost immunization with BNT164 candidates elicited antibody and/or T cell responses against all antigens in three mouse strains (C57BL/6, BALB/c and HLA-A2.1/DR1 humanized mice). The candidates demonstrated favorable safety profiles in a rat toxicity study and significantly reduced bacterial burdens of two *M. tuberculosis* strains in murine aerosol challenge models. BNT164 protection was correlated with granuloma infiltration by CD8^+^ T cells with memory precursor phenotypes. In conclusion, BNT164a1 and BNT164b1 were immunogenic, well tolerated and efficacious in preclinical models and have entered phase 1/2 clinical trials (NCT05537038, NCT05547464).

## Abstract (web summary)

Here the authors characterize mRNA-based tuberculosis vaccine candidates BNT164a1 and BNT164b1 and show that they are immunogenic, well tolerated and efficacious in preclinical animal models.

## Main

Tuberculosis (TB) is the leading cause of death by a single infectious agent, *Mycobacterium tuberculosis* (*Mtb*), and caused approximately 1.23 million deaths in 2024^1^. The standard antibacterial therapy for TB is lengthy, including multiple antibiotics with a variety of associated side effects, and can lead to emergence of drug-resistant bacteria. The only licensed TB vaccine is Bacillus Calmette–Guérin (BCG), a live attenuated vaccine derived from *Mycobacterium bovis*, which is routinely administered in countries in which TB is endemic. Although BCG provides protection from severe forms of TB (for example, meningeal and disseminated) in childhood, it has limited to no protective efficacy against pulmonary TB in adolescents and adults and is contraindicated in immunocompromised individuals^2,3^. In addition to representing the highest burden of disease, pulmonary TB in adolescents and adults is responsible for the majority of TB transmission^4^. Thus, a well-tolerated, efficacious and durable vaccine is urgently needed to prevent TB disease in these populations.

*Mtb* infection manifests as a spectrum from asymptomatic to symptomatic disease, with outcomes ranging from bacterial clearance to dissemination and death^5^. Even within an individual, highly heterogenic lung lesions coexist independently^5,6^. This heterogeneity is dictated by an interplay between the host immune system and metabolic adaptations of the bacteria, resulting in active replication, nonreplication and reactivation^6^. Preventing TB disease in adolescents and adults is therefore likely to require a vaccine that targets multiple antigens expressed throughout *Mtb* infection states.

The lack of correlates of protection in humans further complicates vaccine development^7^. Preclinical data suggest that antigen-specific CD4^+^ T helper 1 (T_H_1) cells and IFNγ and TNF secretion are involved in suppression of *Mtb* infection^8,9^. Indeed, HIV infection^8^, genetic deficiencies in IFNγ pathways^10^ and anti-TNF therapy^11^ increase patients’ risk of developing TB. However, an increasing body of evidence indicates that T follicular helper-like cells, T_H_1 and T helper 17 (T_H_17) cells, MHC-restricted or nonrestricted *Mtb*-specific CD8^+^ T cells, and many other immune cell (sub)types also have protective roles in TB^8,9,12^. Protection conferred by humoral and B cell responses has also been studied, although the scope and mechanisms of this protection remain to be determined^13–15^.

We hypothesized that mRNA-based platforms would be appropriate for TB vaccine development, as they can induce robust cellular (CD4^+^ and CD8^+^) and antibody responses^16^. mRNA vaccines developed against COVID-19 may also provide valuable platform learnings that could be applied to TB vaccine development^17–19^. Furthermore, mRNA allows encoding of multiple antigens and can be rapidly scaled up for clinical vaccine production^20^. Different mRNA chemistries have been previously investigated in humans: (1) *N*^1^-methylpseudouridine-containing modified RNA (modRNA), which is used in two currently licensed mRNA vaccines against COVID-19; and (2) nucleoside-unmodified RNA (uRNA), which was considered in early clinical trials of COVID-19 vaccine development (NCT04380701) and is currently being explored in oncology^21,22^. These two RNA platforms differ in the way they interact with pattern recognition receptors^23^ and thus in their adjuvant activity, with potential effects on vaccine effectiveness^24–26^. Although data obtained for other indications cannot be used to predict immunogenicity or efficacy of a TB vaccine, the promising characteristics of both of these platforms led us to explore them in a TB-specific setting.

Here we designed two multiantigen TB vaccine candidates, BNT164a1 and BNT164b1, based on mRNA–lipid nanoparticle (mRNA–LNP) platforms. The candidates encode the same eight *Mtb* antigens and only differ in the RNA chemistry (uRNA for BNT164a1 or modRNA for BNT164b1). The aim of this study was to evaluate the immunogenicity, safety and efficacy of BNT164a1 and BNT164b1 in preclinical models. Immunogenicity was tested in three mouse strains with different MHC backgrounds (C57BL/6, BALB/c and HLA-A2.1/DR1 humanized mice). Safety was evaluated in a Good Laboratory Practice (GLP)-compliant toxicity study in rats, whereas efficacy against two different *Mtb* isolates was tested in low-dose aerosol challenge models in C57BL/6 mice.

## Results

### Vaccine design

**Fig. 1:**
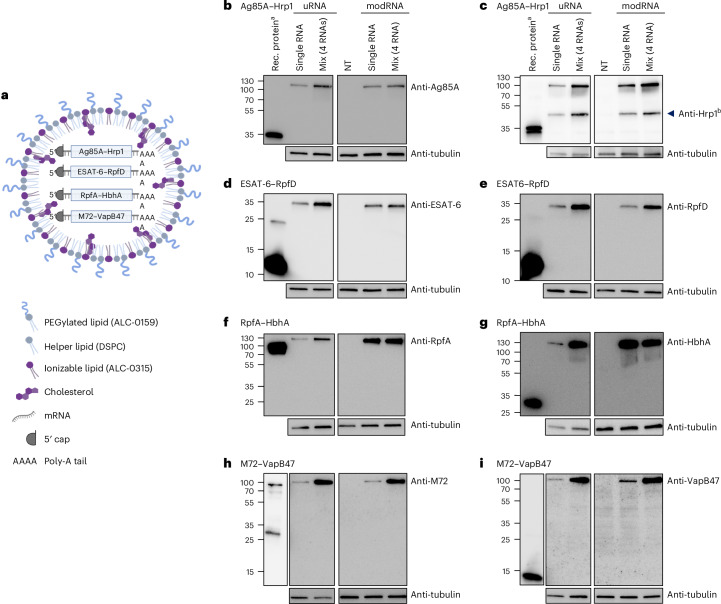
mRNAs encoding fusion *Mtb* antigens are successfully translated into corresponding proteins in vitro. **a**, Schematic illustration of BNT164 vaccine candidates. Four mRNAs, each encoding a fusion of two *Mtb* antigens, were formulated as lipid nanoparticles. **b**–**i**, HEK293T cells were transfected with BNT164 mRNAs either as a single mRNA (0.25 µg ml^−1^) or a manually generated mixture of the four mRNAs (1 µg ml^−1^) using a RiboJuice transfection kit. Nontransfected cells were used as a negative control, and respective recombinant proteins were used as positive controls. Protein expression was assessed by western blot using antigen-specific antibodies: anti-Ag85A (**b**), anti-Hrp1 (**c**), anti-ESAT-6 (**d**), anti-RpfD (**e**), anti-RpfA (**f**), anti-HbhA (**g**), anti-M72 (**h**) or anti-VapB47 (**i**). The western blots shown are representative of three individual experiments. The expected molecular weights were as follows: Ag85A–Hrp1, 50 kDa (**b**,**c**); ESAT-6–RpfD, 28 kDa (**d**,**e**); RpfA–HbhA, 120 kDa (**f**,**g**); and M72–VapB47, 86 kDa (**h**,**i**). The black arrow indicates the Ag85A–Hrp1 monomer. The loading control was tubulin. In **h** and **i**, brightness and contrast were adjusted independently for lanes containing recombinant protein controls to improve visualization of the bands. Uncropped blot images are provided in the source data. ^a^Asterisk indicates that the recombinant protein tested in each individual blot was matched to the detection antibody. ^b^Double asterisk indicates that the anti-Hrp1 antibody detected both monomeric and dimeric Ag85A–Hrp1 fusion protein. NT, nontransfected; rec., recombinant. Panel **a** created in BioRender; Vukovic, N. https://biorender.com/zg085sb (2026). Source data

A multiantigen strategy was adopted for the design of BNT164a1 and BNT164b1 to ensure broad immune coverage. Each candidate contained eight *Mtb* antigens (Ag85A, ESAT-6, VapB47, Hrp1, RpfA, RpfD, M72 and HbhA) distributed among four mRNAs, with each mRNA encoding a fusion of two antigens (Fig. 1a). These antigens are expressed during different stages of infection and were immunogenic and protective against TB in different models (Extended Data Table 1). Six of them (Ag85A, ESAT-6, VapB47, Hrp1, RpfA, and RpfD) are part of a cytomegalovirus vector vaccine candidate that has shown protection in nonhuman primates (NHP) (41% sterilizing immunity)^27^, whereas M72 (a fusion of the *Mtb* antigens PepA/Mtb32A and PPE18/Mtb39A) has shown efficacy against TB in a phase 2b clinical trial^28^. HbhA, a surface protein involved in extrapulmonary dissemination of *Mtb*^29^, was included as a potential antibody and T cell target. Owing to the high HLA diversity of populations in TB endemic regions, all antigens were encoded as full-length proteins, excluding amino-terminal methionines and predicted signal peptides (Extended Data Table 1), to provide multiple CD4^+^ and CD8^+^ T cell epitopes.

**Extended Data Fig. 1:**
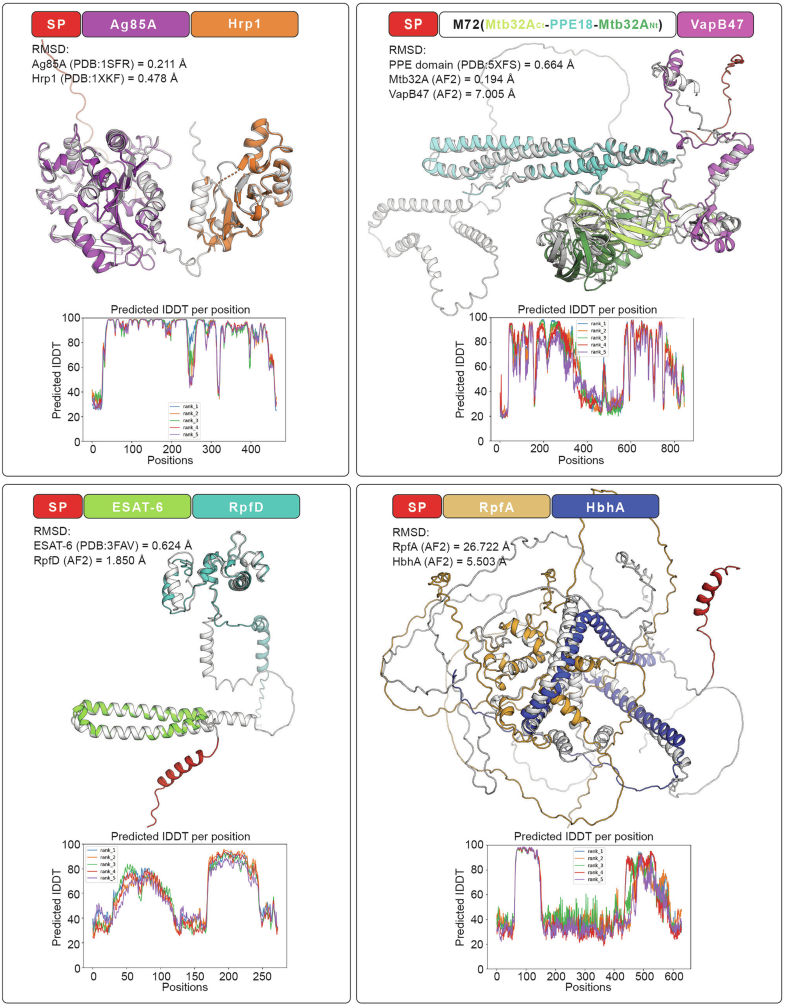
Protein structural visualization of the BNT164 vaccine antigens. Structural models are shown for the full-length fusion antigen cassettes of BNT164 vaccine candidates (depicted in white cartoon representation) that were predicted by AlphaFold2 (AF2) and aligned with experimental structures (when available) or AF2 models of individual antigens (depicted in cartoon representation and colored by antigen). Five independent models were generated and compared for each fusion antigen to identify areas of agreement and better understand modeling robustness. The top ranked model was used for alignment and root mean square deviation (RMSD) calculation in Pymol; RMSD is shown in Angstroms (Å). Predicted local distance difference test (LDDT) values for each ranked model were generated and plotted (colored lines) via AlphaFold2 to depict model confidence, with higher scores indicating higher confidence. SP, signal peptide.

To better understand how the BNT164 antigen design strategy could affect protein conformation and antigenicity, we generated structure predictions of the four chimeric fusion antigens with AlphaFold2^30^ and aligned them to protein structures of single antigens (Extended Data Fig. 1). For antigens for which experimental structures were available (Ag85A, Hrp1, ESAT-6 and the PPE domain within M72), there was a high level of overlap between the chimeric fusion protein models and structures of single antigens, with calculated root mean square deviation (r.m.s.d.) values of <1 Å. We observed poor alignment, indicated by higher r.m.s.d. values, for antigens and domains lacking experimental structures and for regions predicted to be unstructured. Regions of lower structural overlap between single and fusion antigens were especially concentrated in domains with low model confidence, as indicated by local distance difference testing (Extended Data Fig. 1). These predictions suggest that the chimeric antigens of BNT164 have the potential to be folded correctly and can access native protein conformations capable of inducing relevant immune responses.

### mRNA-encoded antigens are expressed in vitro

To confirm successful protein expression of BNT164a1 and BNT164b1 mRNA constructs in human cells, we transfected HEK293T cells with either individual mRNAs (each containing two antigens) or a mix of all four mRNAs per vaccine candidate and performed western blot analysis. All eight mRNA-encoded *Mtb* antigens were detected in HEK293T cell lysates at the expected molecular weights (calculated for fusion proteins) (Fig. 1b–i). Anti-Hrp1 detection revealed the presence of two bands with molecular weights corresponding to monomeric and dimeric Ag85A–Hrp1 fusion protein. Dimerization of Hrp1 has been previously reported^31^, and our data indicate that this was not affected by fusion to Ag85A. The monoclonal Ag85A antibody detected only the dimeric version of Ag85A–Hrp1. Protein expression did not decrease for any of the constructs when mRNA was delivered in a mix compared with individual mRNAs, suggesting no major translational interference in vitro. Taken together, these results indicate that the mRNAs used in BNT164 candidates were successfully translated into the corresponding *Mtb* fusion proteins in vitro.

### BNT164 candidates elicit cellular and humoral immunogenicity in mice

**Fig. 2:**
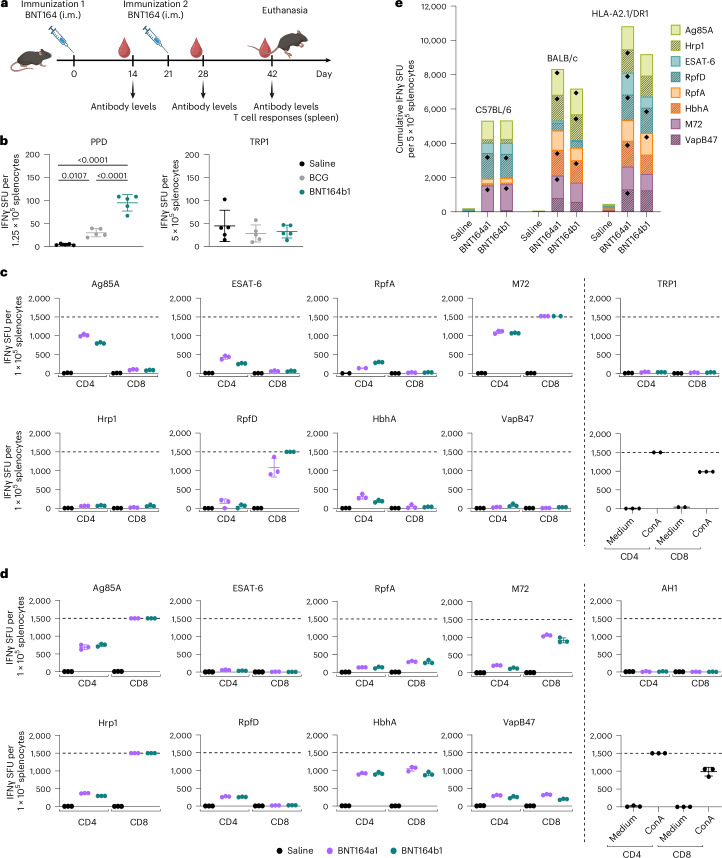
BNT164 vaccine candidates induced T cell responses against purified protein derivative and all target *Mtb* antigens in mice. **a**, Design of immunogenicity studies performed with BNT164 candidates, unless indicated otherwise. **b**–**e**, C57BL/6 mice (**b**,**c**,**e**), BALb/c mice (**d**,**e**) or HLA-A2.1/DR1 mice (**e**) were immunized with a saline control (i.m.) or 4 µg BNT164a1/b1 (i.m.) on days 0 and 21, or with ~10^6^ CFU BCG on day 0 (s.c.; only in **b**). Splenocytes were isolated on day 42 and stimulated with purified protein derivative or TRP1 (nonspecific peptide) (**b**) or with individual *Mtb* antigens (**e**), and responses were assessed by IFNγ ELISpot assay after ~18-h incubation. In **b**, group mean values are indicated by horizontal bars (±s.d.); means from individual mice (*n* = 5 per group, measured in duplicate) are depicted as circles. One-way ANOVA with Tukey’s multiple comparisons test was performed; *P* values are depicted in the figure. In **c** and **d**, splenocytes were isolated on day 42. CD4^+^ and CD8^+^ T cells were magnetically sorted from splenocyte pools from each group (*n* = 5 per group) and stimulated with individual *Mtb* antigens or nonspecific peptides (TRP1 and AH1, respectively). Media only and concanavalin A were used as assay controls. BMDCs from nonvaccinated mice (unrelated cohort of naive C57BL/6 or BALB/c mice) were cocultured with T cells as antigen-presenting cells. Responses were assessed by IFNγ ELISpot assay after ~18-h incubation. Circles represent duplicate or triplicate measurements; horizontal bars represent group mean ± s.d. No statistical analysis was performed to evaluate the differences, as the data were technical replicates of pooled samples within each treatment group. In **e**, bars indicate mean splenocyte responses from individual mice (*n* = 5 per group except for BNT164a1 HLA-A2.1/DR1 mice (*n* = 4), measured in duplicate) for each antigen. The horizontal dashed line (**c**,**d**) or rhombus symbol (**e**) indicate conditions in which the upper limit for the number of spots that could be correctly counted was reached (~1,500 spots). ConA, concanavalin A; PPD, purified protein derivative; SFU, spot-forming unit. Panel **a** created in BioRender; Vukovic, N. https://biorender.com/dbrt21u (2026).

We assessed BNT164 immunogenicity by analyzing T cell and B cell responses in a series of experiments in different mouse strains, following the immunization scheme shown in Fig. 2a. The dosing regimen was based on internal experience with mRNA vaccine design, such as that described in ref. ^16^.

#### T cell responses

For a preliminary assessment of T cell immunogenicity, we compared day 42 splenic T cell responses induced by BCG (subcutaneous (s.c.) immunization, day 0) and BNT164b1 (intramuscular (i.m.) immunization, days 0 and 21) in C57BL/6 mice, upon ex vivo restimulation with purified protein derivative in an ELISpot assay. Whereas both BCG and BNT164b1 induced significantly higher numbers of IFNγ-secreting T cells compared with saline, BNT164b1 was superior to BCG (Fig. 2b). Stimulation with unrelated peptide TRP1 served as a control for nonspecific responses and induced low numbers of IFNγ-secreting cells in all test groups (Fig. 2b).

We next analyzed individual T cell responses against each antigen encoded by BNT164 candidates in mice immunized with BNT164a1, BNT164b1 or saline. IFNγ secretion from total splenocytes or purified CD4^+^ or CD8^+^ T cells was measured using ELISpot assay 3 weeks after the boost (day 42). BNT164a1 and BNT164b1 raised CD4^+^ or CD8^+^ T cell responses, or both, against each of the eight *Mtb* antigens in C57BL/6 and/or BALB/c wild type mice (Fig. 2c,d). T cell responses against Ag85A, Hrp1 and HbhA were generally higher in BALB/c mice than in C57BL/6, whereas responses against M72 and RpfD were higher in C57BL/6 mice. Stimulation with nonspecific peptides TRP1 and AH1, or medium only, induced minimal CD4^+^ or CD8^+^ T cell responses (Fig. 2c,d).

**Extended Data Fig. 2:**
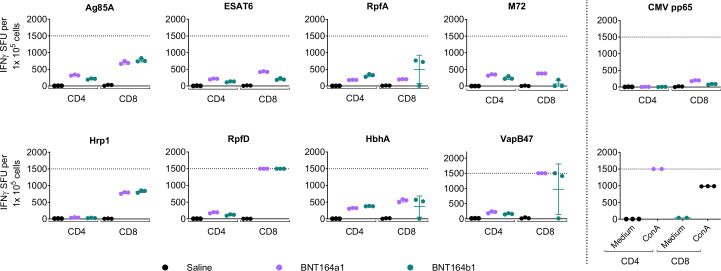
BNT164 candidates induced antigen-specific CD4^+^ and/or CD8^+^ T cell responses in HLA-A2.1/DR1 humanized mice. Splenocytes were isolated on Day 42 from mice injected twice (Days 0 and 21) i.m. with 4 µg of BNT164a1, 4 µg of BNT164b1, or a saline control. CD4^+^ and CD8^+^ T cells were magnetically sorted from splenocytes pooled from each treatment group (n = 5/group) and stimulated with individual Mtb antigens, medium only, concanavalin A (con A), or non-specific peptide (CMVpp65). Bone marrow-derived dendritic cells from non-vaccinated mice (unrelated cohort of naïve mice) were co-cultured with T cells as antigen-presenting cells. The responses were assessed by IFNγ ELISpot assay after ~18 hours incubation. Circles represent duplicate/triplicate measurements; horizontal bars represent group means ± standard deviation. SFUs for some samples reached the upper limit in number of spots that can be correctly counted (indicated with the dashed horizontal line). SFU, spot-forming unit.

**Extended Data Fig. 3:**
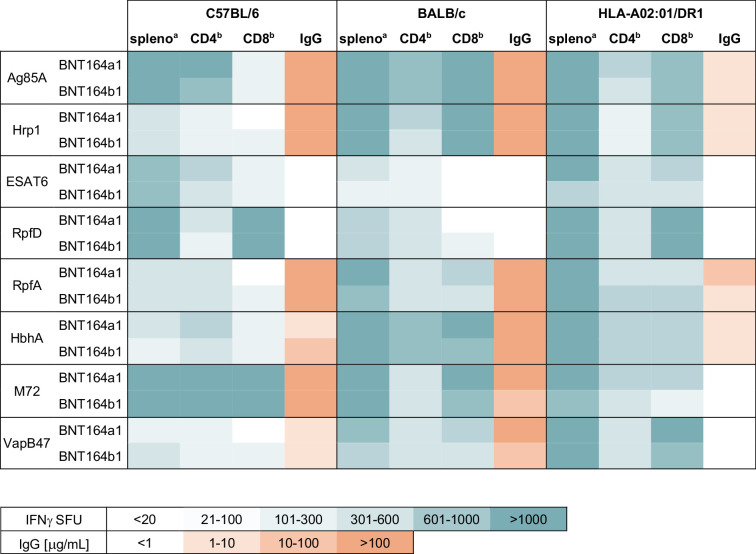
Heatmap overview of BNT164-induced immune responses. BNT164a1 and BNT164b1 immunogenicity in three different mouse models following prime-boost immunization. Green shades: T cell responses measured via IFNγ ELISpot using ^a^ total splenocytes (SFU/5 × 10^5^ cells) or ^b^ purified splenic CD4^+^ or CD8^+^ T cells (SFU/ 1 × 10^5^ cells). Orange shades: IgG serum concentrations measured via ELISA. Data are presented in Supplementary Fig. 1, Fig. 2c, d, Extended Data Fig. 2, Fig. 3a, and Extended Data Fig. 4.

To understand potential immunogenicity for certain human HLA alleles, we next used HLA-A2.1/DR1 humanized mice in which endogenous MHC molecules had been knocked out and human HLA-A2.1 and HLA-DR1 expressed instead^32^. In this model, BNT164 candidates raised antigen-specific T cell responses against all antigens (Extended Data Fig. 2). ELISpot analysis using total splenocytes showed results consistent with the trends observed with purified CD4^+^ and CD8^+^ splenic T cells in all three mouse models, but there were overall higher responses (Supplementary Fig. 1 and summary in Fig. 2e and Extended Data Fig. 3). Taken together, these results indicate that all BNT164-encoded antigens raised T cell responses in at least two of three tested mouse models, with observed differences likely to have been driven by MHC/HLA variations between these mouse models.

For a more detailed analysis of T cell responses, we quantified the percentages of CD4^+^ and CD8^+^ cells producing IFNγ, IL-2 or TNF, or all three cytokines, as well as T cell memory subsets, via flow cytometry 3 weeks after the boost (day 42). Following splenocyte stimulation with a pool of peptides against all eight *Mtb* antigens, we observed significantly higher percentages of single cytokine-secreting and polyfunctional (IFNγ^+^IL-2^+^TNF^+^) CD4^+^ and CD8^+^ T cells in C57BL/6 mice immunized with BNT164 candidates compared with saline (Supplementary Fig. 2). Although there was no difference between candidates in terms of elicited CD4^+^ responses, BNT164a1 induced a significantly higher percentage of CD8^+^ cytokine-secreting T cells than BNT164b1 (Supplementary Fig. 2). Analysis of T cell memory subsets revealed that BNT164 candidates induced both memory precursor effector cells (MPEC; cytokine^+^CD127^+^KLRG1^−^CD62L^−^) and long-lived memory precursor (LLMP; cytokine^+^CD127^+^KLRG1^−^CD62L^+^) cells (Supplementary Fig. 2). Similar to observations with cytokine-producing T cells, BNT164a1 induced significantly higher percentages of CD8^+^ memory precursor cells compared with BNT164b1.

Taken together, these results indicate that BNT164 candidates induced cellular immunity against the selected *Mtb* antigens in multiple tested mouse strains following prime-boost dosing.

#### IgG responses

**Fig. 3:**
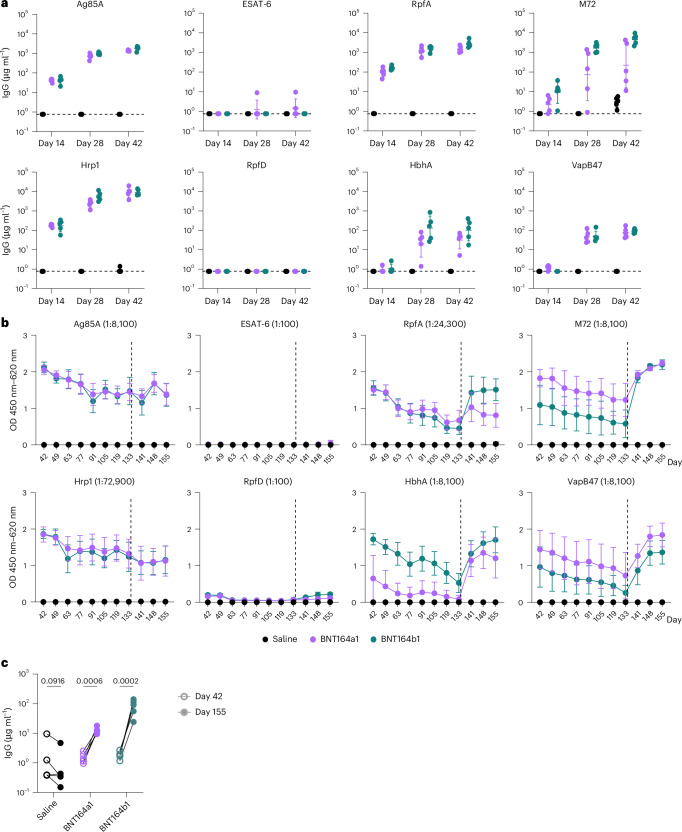
BNT164 vaccine candidates induced IgG responses against majority of vaccine antigens in C57BL/6 mice. **a**–**c**, C57BL/6 mice received i.m. injection of 4 µg BNT164a1, 4 µg BNT164b1 or a saline control on days 0 and 21 (**a**) or days 0, 21 and 134 (**b**,**c**). Sera isolated on the indicated days were used in ELISA to measure antigen-specific IgG antibodies using recombinant *Mtb* proteins (**a**,**b**), or anti-*Mtb* IgG antibodies using *Mtb* H37Rv lysate (**c**). In **a** and **b**, group mean values are indicated by horizontal bars (±s.d.); means from individual mice (*n* = 5 per group, measured in duplicate) are depicted as circles. The horizontal dashed line in **a** indicates the lower limit of detection; the vertical dashed line in **b** indicates the timing of the third immunization. In **c**, means from individual mice (*n* = 5 per group, measured in duplicate) are depicted as circles; lines connect the same mouse on different days. Differences between days for each group were compared with ratio-paired *t*-tests corrected for multiple comparisons by false discovery rate calculation using Benjamini, Krieger and Yekutieli methodology.

**Extended Data Fig. 4:**
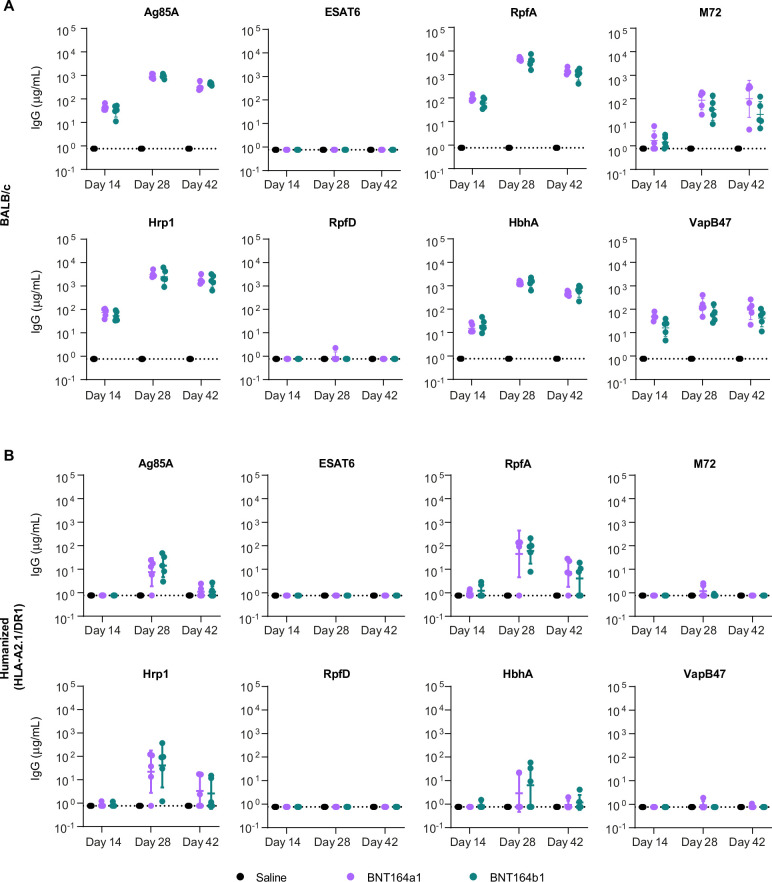
IgG responses in BALB/c and HLA-A2.1/DR1 humanized mice following prime-boost immunization with BNT164 candidates. BALB/c mice (**a**) and HLA-A2.1/DR1 humanized mice (**b**) were injected i.m. with 4 µg BNT164a1, 4 µg BNT164b1, or saline control on Days 0 and 21. Antigen-specific IgG antibodies were assessed by ELISA from sera isolated on indicated days. Group mean values are indicated by horizontal bars (±standard deviations), means from individual mice (n = 5/group, measured in duplicates) are depicted as circles. Dashed line indicates lower limit of detection (LLOD). In (**b**) one mouse in BNT164a1 group had to be sacrificed before the end of the experiment due to treatment-unrelated health issues and could not be included in the analysis.

We assessed BNT164-induced humoral responses in sera of C57BL/6, BALB/c and HLA-A2.1/DR1 humanized mice using ELISA. In C57BL/6 (Fig. 3a) and BALB/c (Extended Data Fig. 4a) mice, prime-boost immunization with BNT164a1 or BNT164b1 elicited IgG responses against all *Mtb* target antigens, except ESAT-6 and RpfD. IgG responses were mostly detectable by day 14 after the prime dose, and titers increased until the end of experiment on day 42. We detected comparable levels of IgG antibodies against Ag85A, RpfA and Hrp1 in both mouse models but observed some strain-specific differences in IgG responses against M72 (higher in C57BL/6) and HbhA (higher in BALB/c). As expected, in HLA-A2.1/DR1 humanized mice, BNT164a1 and BNT164b1 immunization induced only marginal IgG responses against a few antigens (Extended Data Fig. 4b). An overview of BNT164-induced T cell and IgG responses across all three mouse models is provided in Extended Data Fig. 3.

#### Antigen interference

**Extended Data Fig. 5:**
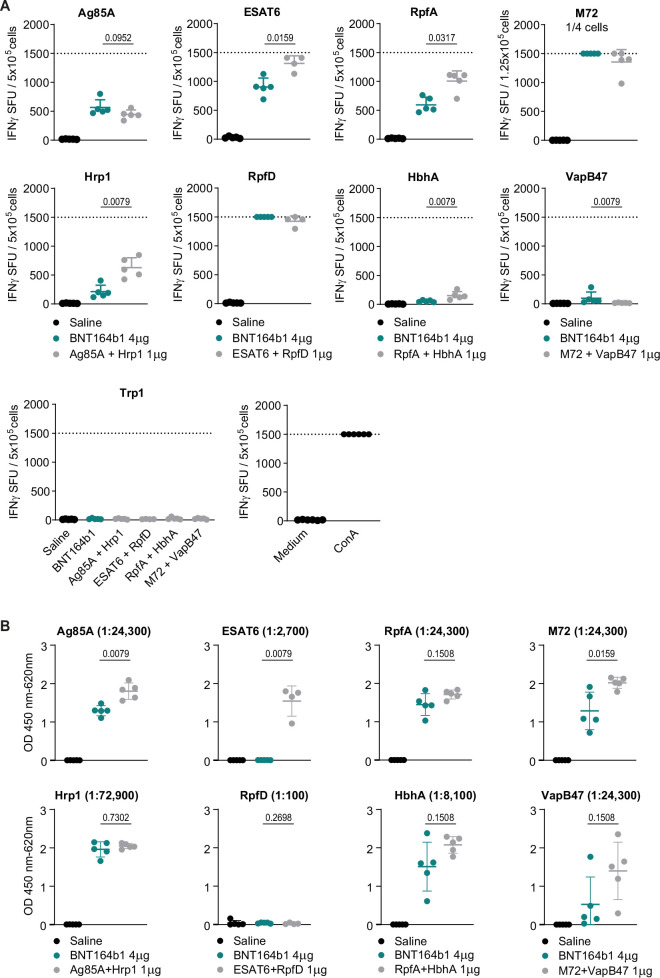
Modest antigen interference in BNT16b1 immunogenicity. C57BL/6 mice received i.m. injection of 4 µg BNT164b1, 1 µg of individual BNT164b1 mRNA cassettes formulated in the same LNP as BNT164b1, or saline control on Days 0 and 21. (**a**) Splenocytes were isolated on Day 42 and stimulated with antigen-specific peptide pools, medium only, concanavalin A (con A), or non-specific peptide TRP1. Responses were assessed by IFNγ ELISpot assay after ~18 hours incubation. (**b**) Antigen-specific IgG antibodies were assessed by ELISA from sera isolated on Day 42. (**a-b**) Group mean values are indicated by horizontal bars ( ± standard deviations), means from individual mice (n = 5/group, except for ESAT-6+RpfD n = 4) measured in duplicates) are depicted as circles. In (**a**), technical replicates are shown for stimulation with medium or concanavalin A. Two-sided Mann-Whitney test was performed to statistically compare the BNT164b1 group against individual mRNA cassettes groups. In (**a**), SFUs for some samples reached the upper limit in number of spots that can be correctly counted (indicated with dashed horizontal line) and were not statistically analyzed. LNP, lipid nanoparticle; SFU, spot-forming unit.

For multivalent vaccine candidates, it is important to investigate antigen interference. After confirming that there was no interference at the expression level (Fig. 1), we investigated interference at the immunogenicity level in vivo. To this end, we compared BNT164b1 (4 µg) and four individual antigenic cassettes of BNT164b1 (1 µg) in a prime-boost regimen in C57BL/6 mice. For T cell responses, modest antigen interference was observed for Hrp1, ESAT-6, RpfA and HbhA, as mice immunized with individual cassettes showed higher responses than BNT164b1-immunized mice (Extended Data Fig. 5a). IgG responses were overall comparable between groups, except for ESAT-6 (Extended Data Fig. 5b). Whereas immunization with the individual cassette induced an IgG response to ESAT-6, this response was abrogated by combination with the other three cassettes in BNT164b1. As ESAT-6 was primarily included in BNT164 as a T cell antigen^27^, loss of ESAT-6 antibody responses was considered acceptable. Taken together, these results indicate that interference between the eight selected BNT164 antigens was acceptable for an octavalent vaccine, with most of the antigen-specific immune responses being preserved.

#### Durability and boostability

**Extended Data Fig. 6:**
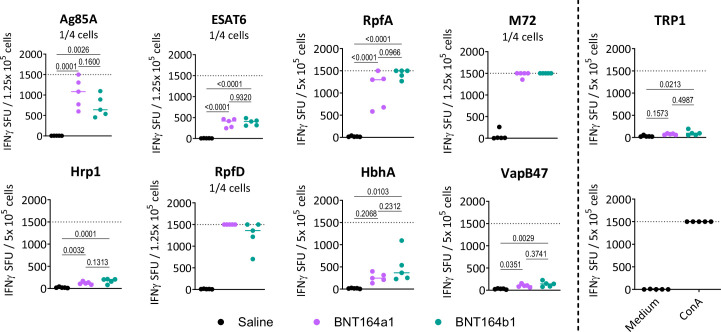
Splenic T cell responses in C57BL/6 mice following three doses of BNT164 candidates. C57BL/6 mice received i.m. injection of 4 µg BNT164a1, 4 µg BNT164b1, or saline control on Days 0, 21, and 134. Splenocytes were isolated on Day 155 and stimulated with antigen-specific peptide pools, medium only, concanavalin A (con A), or non-specific peptide TRP1. Responses were assessed by IFNγ ELISpot assay after ~18 hours incubation. Group mean values are indicated by horizontal bars ( ± standard deviations), means from individual mice (n = 5/group, measured in duplicates) are depicted as circles. For stimulation with medium or concanavalin A, technical replicates are shown. One-way ANOVA with Tukey’s multiple comparisons test was performed. SFUs for some samples reached the upper limit in number of spots that can be correctly counted (indicated with dashed horizontal line) and were not statistically analyzed. SFU, spot-forming unit.

Next, we investigated the long-term kinetics of IgG responses in C57BL/6 mice immunized with two BNT164a1 or BNT164b1 doses. Biweekly monitoring revealed a modest gradual decrease in antibody titers from day 42 to day 133 (Fig. 3b). For antigens for which titers decreased the most (RpfA, HbhA, M72 and VapB47), a third BNT164a1 or BNT164b1 immunization on day 134 successfully boosted responses to levels similar to or higher than those observed on day 42 (Fig. 3b). Similarly, splenic T cell responses 3 weeks after the third BNT164a1/b1 dose (Extended Data Fig. 6) were comparable to or higher than those obtained 3 weeks after the second dose (Supplementary Fig. 1). The greatest increases in splenocyte response were observed for Ag85A, ESAT-6, RpfD, RpfA and M72 following the three-dose schedule.

Finally, to confirm that BNT164-induced antibodies could bind to *Mtb* antigens in their native conformation, we performed an *Mtb* H37Rv lysate ELISA with sera of immunized mice. *Mtb* lysate likely does not include Hrp1, VapB47, RpfA and RpfD, as these antigens are not expressed in general culture conditions. IgG binding to *Mtb* lysate was observed at relatively low levels at day 42 for all BNT164-immunized animals and one saline-injected animal (Fig. 3c). The IgG response against the *Mtb* lysate increased significantly between days 42 and 155 in both BNT164a1 (8-fold) and BNT164b1 (40-fold) groups. These data show that the third BNT164 dose improves the binding of induced antibodies to native bacterial antigens within *Mtb* lysate.

### BNT164 candidates show a favorable safety profile in rats

**Fig. 4:**
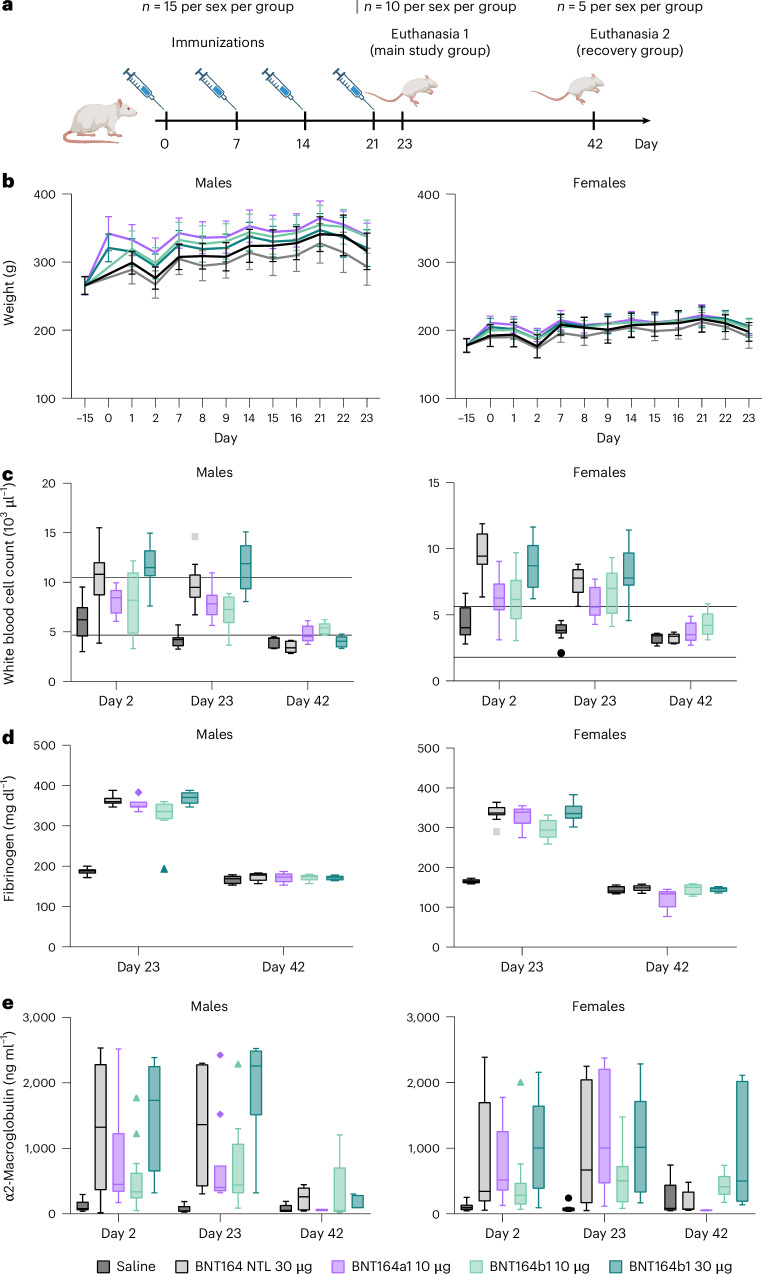
BNT164 candidates were well tolerated in a repeat-dose toxicity study in Wistar Han rats. **a**, Schematic representation of the study. Wistar Han rats were injected i.m. with saline, BNT164 NTL (30 µg), BNT164a1 (10 µg) or BNT164b1 (10 µg or 30 µg) on days 0, 7, 14 and 21. Rats in the main study group were euthanized on day 23 (*n* = 10), whereas those in the recovery group were euthanized on day 42 (*n* = 5). Some samples were unsuitable for hematological analysis following coagulation and/or owing to unreliable instrumental data; these are indicated in Methods. **b**, Animal weights (mean ± s.e.m.). **c**–**e**, Tukey box plots of laboratory findings for white blood cell count (**c**), fibrinogen (**d**) and α2-macroglobulin (**e**), showing group medians (middle line), 25th and 75th percentiles (box), upper and lower adjacent values (whiskers) and outliers (symbols). Horizontal lines in **c** represent the range of historical values for white blood cells for this strain at the study site. Panel **a** created in BioRender; Vukovic, N. https://biorender.com/1ycit4s (2026).

To evaluate the safety profiles of BNT164a1 and BNT164b1, we performed a GLP-compliant toxicity study in which rats received four weekly i.m. injections of BNT164a1 (10 µg), BNT164b1 (10 µg or 30 µg), saline or BNT164 nontranslatable control (NTL) (30 µg, modRNA). Toxicity was assessed at two time points: rats in the main study group were euthanized 2 days after the last immunization, whereas those in the recovery group were euthanized 3 weeks after the last immunization to study the reversibility of vaccine-induced changes (Fig. 4a).

**Extended Data Fig. 7:**
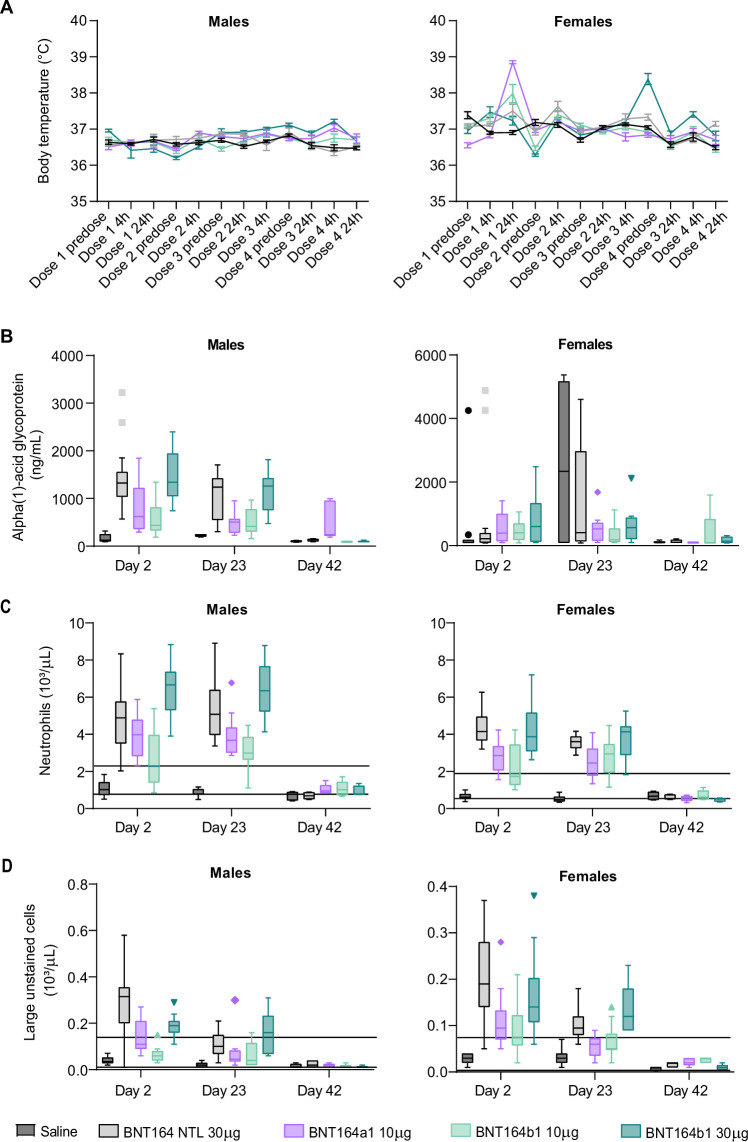
Additional readouts from a repeat-dose toxicity study in Wistar Han rats. Wistar Han rats were injected i.m. with saline, BNT164 non-translatable control (NTL, 30 µg), BNT164a1 (10 µg), or BNT164b1 (10 µg or 30 µg), on Days 0, 7, 14, and 21. Main study group was sacrificed on Day 23 (n = 10), while the recovery group was sacrificed on Day 42 (n = 5). Some samples were unsuitable for hematological analysis following coagulation and/or following unreliable instrumental data and are indicated in the method section. (**a**) Animal rectal temperature (mean ± standard error of the mean). (**b-d**) Laboratory findings plotted with Tukey box plots showing group medians (middle line), 25th and 75th percentiles (box), upper and lower adjacent values (whiskers), and outliers (symbols). Horizontal lines in (**c**) and (**d**) represent historical values range for this strain at study site.

We did not observe any vaccine-related systemic clinical signs or mortalities, or any vaccine-related changes in body weight (Fig. 4b) or in the urinalysis. In males, there were no remarkable changes in body temperature throughout the study, whereas increases in body temperature were observed in females 24 h after the first injection of BNT164a1 and 24 h after the third injection of 30 µg BNT164b1 (Extended Data Fig. 7a). Macroscopic observations of the injection sites did not reveal redness, swelling, scabbing or other macroscopic alterations. However, histological examination showed muscle fiber damage and inflammatory reactions in animals injected with 30 µg NTL, 10 µg BNT164a1 or 30 µg BNT164b1 that partially or completely resolved by day 42 (Supplementary Fig. 3). On days 2 and 23, the BNT164 candidates induced increased levels of white blood cells, neutrophils, large unstained cells and acute phase proteins (fibrinogen, α2-macroglobulin and α1-acid glycoprotein), indicating expected inflammatory responses to a vaccine (Fig. 4c–e and Extended Data Fig. 7b–d). These changes were transient: all values returned to normal by day 42, except for α2-macroglobulin levels in some animals treated with BNT164b1 (both doses) and α1-acid glycoprotein levels in some males treated with BNT164a1 and some females treated with 10 µg BNT164b1.

All observed changes were dose-dependent, as the animals vaccinated with 30 μg BNT164b1 showed a greater increase of inflammatory parameters compared with animals vaccinated with 10 μg BNT164b1. Animals injected with NTL also showed an increase in inflammatory parameters (comparable to that seen with BNT164b1 at the same dose), indicating that the effects were formulation-dependent rather than antigen-specific. Overall, BNT164 candidates were well tolerated and induced expected inflammatory reactions that mostly resolved within 3 weeks of the last dose.

### BNT164 candidates reduce the bacterial burden in *Mtb*-challenged mice

**Fig. 5:**
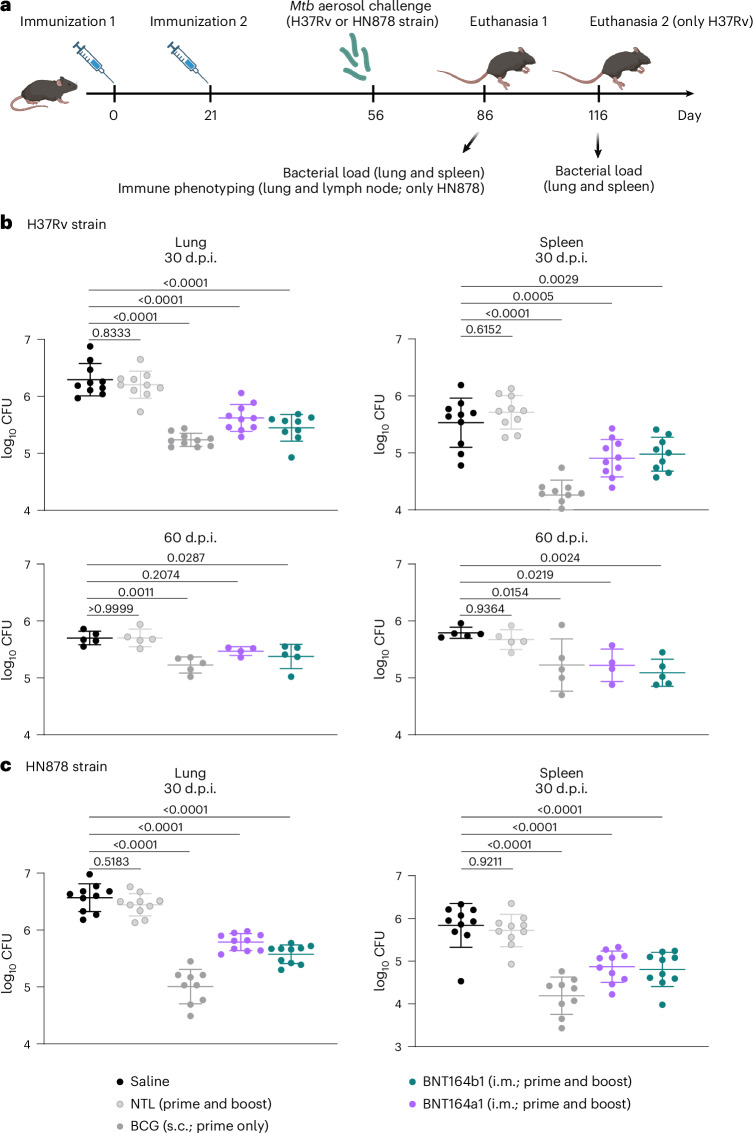
BNT164 candidates significantly reduced the bacterial load in *Mtb*-challenged mice. **a**, Schematic representation of the study. C57BL/6 mice were injected with saline (i.m., days 0 and 21), BCG (s.c. 10^6^, day 0) or mRNA–LNP (i.m. 4 µg, days 0 and 21). On day 56, mice were aerosol-challenged with ~100 CFUs of *Mtb* H37Rv or HN878. The left lobe of the lung and the spleen were harvested, processed into a homogenate, serially diluted onto 7H11 agar and cultured to enumerate viable bacteria on day 86 (30 days postinfection (d.p.i.)) or day 116 (60 d.p.i.). **b**,**c**, CFUs in lungs (left) and spleen (right). In **b** (top) and **c**, *n* = 10 for all groups (except group BNT164b1 in **b** and BCG in **c** (*n* = 9)). In **b** (bottom), *n* = 5 (except for BNT164a1 (*n* = 4)). Group mean values are indicated by horizontal bars (±s.d.); individual mouse values are depicted as circles. One-way ANOVA with Dunnett’s multiple comparisons test between the saline-treated group and all other groups was performed. Panel **a** created using BioRender; Vukovic, N. https://biorender.com/h4d96wj (2026).

To assess the efficacy of BNT164 candidates, we used a low-dose aerosol *Mtb* challenge model with two different *Mtb* strains: reference strain H37Rv (genetic lineage L4) and hypervirulent HN878 (genetic lineage L2). Both L2 and L4 are globally dispersed^33^ and although genetically very similar (>90% sequence identity) show phenotypic differences in antigenicity, pathogenesis and vaccine protection^34,35^. BNT164-immunized or saline-injected C57BL/6 mice were exposed to ~100 colony-forming units (CFUs) of H37Rv or HN878 *Mtb* at week 8 of the experiment, and the bacterial load in lung (site of infection) and spleen (extrapulmonary site) was determined 30 days postinfection (d.p.i.) for both strains and 60 d.p.i. only for H37RV (Fig. 5a). Nontranslatable BNT164 modRNA formulated in LNP (NTL) and saline were used as controls.

In H37Rv-infected mice, BNT164a1 and BNT164b1 significantly reduced bacterial burden compared with saline (~0.55–0.85 log reduction) in both lung and spleen at 30 d.p.i. (Fig. 5b). At 60 d.p.i., lung CFUs remained comparable to 30 d.p.i. values in all test groups except the saline and NTL control groups, in which CFU values declined (Fig. 5b). In addition, all test groups showed slight increases in splenic bacterial load at 60 d.p.i. compared with 30 d.p.i. (Fig. 5b). Mice immunized with NTL control did not have significant reductions in bacterial burden compared to the saline group at either time point, indicating that BNT164 candidates reduced the bacterial burden predominantly via antigen-specific immunity (Fig. 5b). Mice immunized with BCG served as a positive control group and showed ~1 log reduction of viable bacteria compared with saline at 30 d.p.i. (Fig. 5b). In HN878-infected mice, BNT164 candidates also significantly reduced the bacterial burden in lung and spleen at 30 d.p.i. compared with saline (~0.8–1.0 log reduction) (Fig. 5c).

**Fig. 6:**
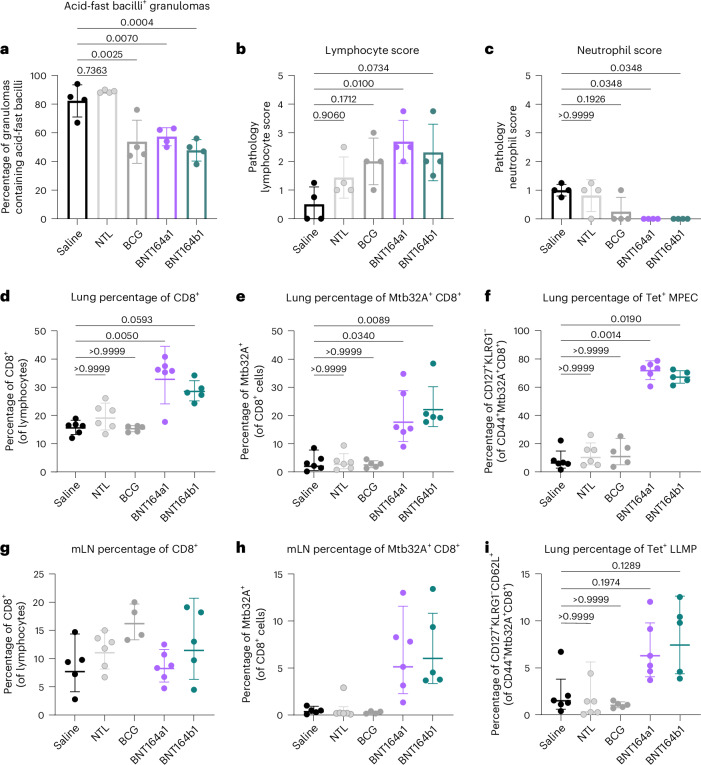
BNT164-induced protection correlates with antigen-specific CD8^+^ T cells with a memory precursor phenotype in the lungs. **a**–**c**, Right lung lobes of the mice depicted in Fig. 5, infected with *Mtb* HN878, were harvested at the time of euthanasia (30 days postinfection). For four mice per group, these lungs were paraffin-embedded and subjected to acid-fast (**a**) or hematoxylin and eosin (**b**,**c**) staining. A pathologist blinded to group identities scored the percentage of granulomas containing acid-fast bacteria (**a**) and scored the immune cell composition (lymphocytes in (**b**) and neutrophils in (**c**)) within granulomas by multiplying the granuloma severity score by cell composition (further details are provided in Methods). **d**–**h**, Right lung lobes and mLNs of the remaining 6 mice (*n* = 5 for BCG-immunized mice) in each group were homogenized and analyzed by flow cytometry. Plotted are percentages of CD8^+^ cells in the lung (**d**) and mLN (**g**), percentages of Mtb32A tetramer^+^ CD8^+^ cells in the lung (**e**) and mLN (**h**), and percentages of Mtb32a tetramer^+^ MPECs (**f**) and LLMPs (**i**) in the lung. Some samples were excluded from analysis as specified in Methods. Plots were analyzed by ANOVA or Kruskal–Wallis test, and significant (*P* < 0.05) results were tested for multiple comparisons of all groups against the saline control using ordinary one-way ANOVA followed by Dunnet´s test (**a**) or Kruskal–Wallis test followed by Dunn´s test (**b**–**i**). MPEC, memory precursor effector cells (CD127^+^KLRG1^−^); LLMP, long-lived memory precursor (CD127^+^KLRG1^−^CD62L^+^).

**Extended Data Fig. 8:**
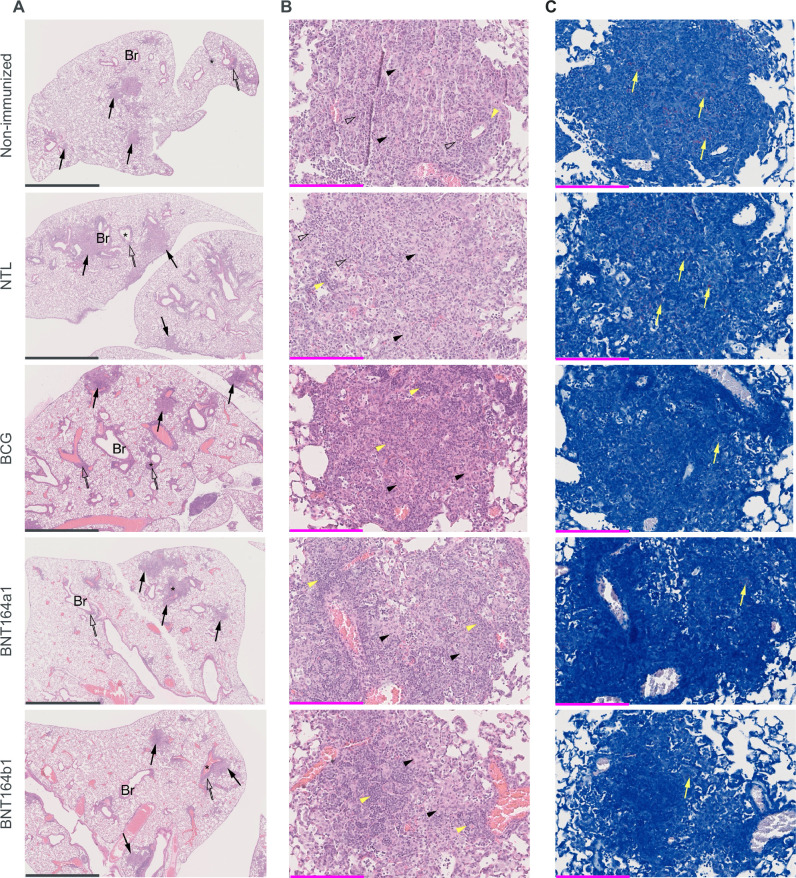
Pathology of *Mtb* HN878 infected mouse lungs. The study design is depicted in Fig. 5a. Right lung lobes from four mice per group were paraffin embedded, formalin fixed and stained with hematoxylin & eosin (**a-b**) or acid-fast staining (**c**). Representative images are shown. (**a**) Closed black arrows indicate granulomas, while open black arrows indicate perivascular/peribronchiolar lymphocyte infiltrate cuffs surrounding either blood vessels (*) or bronchioles (Br). (**b**) Closed black arrowheads point to macrophages, yellow arrowheads point to lymphocytes, and open black arrowheads point to neutrophils. (**c**) Yellow arrows indicate acid-fast bacteria within granulomas. Black scale bars depict 2 mm (**a**), while pink scale bars depict 200 µm (**b-c**).

To assess the effect of BNT164 vaccination on pathology of *Mtb* HN878 infection, lung sections were evaluated histologically (Extended Data Fig. 8). Although all groups had structured granulomas, they differed with respect to the presence of acid-fast bacteria. In the saline- or NTL-injected groups, >80% of granulomas contained acid-fast bacteria, whereas this proportion was significantly lower (~50%) for BCG- and BNT164-immunized groups (Fig. 6a), in line with the bacterial burden obtained by CFU counting (Fig. 5). This reduction in bacterial burden in the groups immunized with BCG or BNT164 candidates was negatively correlated with lymphocyte infiltration (*r* = −0.7785; *P* < 0.0001) (Fig. 6b). By contrast, lung sections from groups injected with saline or NTL showed high numbers of neutrophils, which were not detected in animals immunized with BNT164 candidates (Fig. 6c). Macrophage presence was similar across all groups (Extended Data Fig. 8b).

In summary, BNT164 vaccine candidates significantly reduced *Mtb* burden following aerosol challenge in mice with two different *Mtb* lineages. Protection induced by BNT164 candidates was associated with lymphocytic infiltration into the lungs.

### BNT164 candidates induce CD8^+^ T cells with memory precursor phenotypes

**Extended Data Fig. 9:**
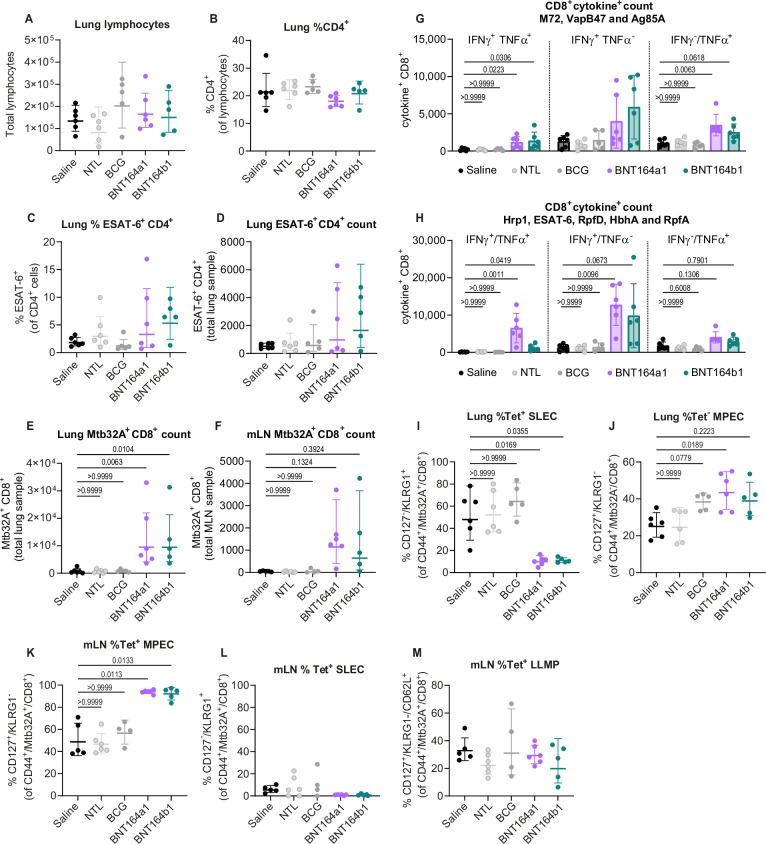
Lymphocyte quantification and phenotype in lungs and mediastinal lymph nodes of *Mtb* HN878 infected mice. The study design is depicted in Fig. 5a. Right lung lobes (**a–e** and **g–j**), and mLNs (**f** and **k–m**) were homogenized 30 days post-infection and analyzed by flow cytometry directly (**a–f** and **i–m**) or after stimulation with overlapping peptide mixes of M72, VapB47, and Ag85A (**g**) or of Hrp1, ESAT-6, RpfD, HbhA, and RpfA (**h**). Plots show percentages or counts of analyzed cell populations as indicated. 6 mice were analyzed for each group except for BCG (n = 5). Some samples were excluded from analysis as specified in the methods. Dots show values from individual animals. **a-f, i-m**: Lines depict geometric means ± geometric SD. G-H: Bars depict mean ± SD. All data were analyzed by Kruskal-Wallis non-parametric ANOVA followed by Dunn´s test for multiple comparisons against the saline control only if p < 0.05. LLMP, Long-lived memory precursor; mLN, mediastinal lymph node; MPEC, Memory precursor effector cell; SLEC, Short-lived effector cell.

In parallel to the pathological assessment, we analyzed small lung lobes from the remaining mice by flow cytometry using a lymphocyte phenotyping panel (Supplementary Fig. 4). Although tetramers in the C57BL/6 background are very limited, the panel included one class I tetramer derived from Mtb32A (a subcomponent of M72) and one ESAT-6 specific class II tetramer. Consistent with microscopical findings, the BNT164-immunized groups showed a higher (although not statistically significant) total number of lung lymphocytes than negative control groups (saline- or NTL-injected) (Extended Data Fig. 9a). This was not driven by increased frequencies of total or antigen-specific CD4^+^ cells (Extended Data Fig. 9b–d) but rather by an increase (approximately two-fold) in CD8^+^ T cell frequencies (Fig. 6d). A large proportion (~20%) of these CD8^+^ cells were activated (CD44^+^) and antigen-specific as shown by the Mtb32A tetramer (Fig. 6e and Extended Data Fig. 9e). Functionality of CD8^+^ lung lymphocytes was confirmed by intracellular staining of IFNγ and TNF after stimulation with two peptide pools of similar amino acid content, encompassing BNT164 antigens. One pool included peptides from M72, VapB47 and Ag85; the other included peptides from Hrp1, ESAT-6, RpfD, HbhA and RpfA. Stimulation with either pool induced significantly higher numbers of IFNγ^+^TNF^+^ double-positive cells compared with the control (Extended Data Fig. 9g–h), confirming that BNT164-induced antigen-specific CD8^+^ T cells are functional.

We further characterized the CD44^+^Mtb32A tetramer^+^CD8^+^ T cells to distinguish between short-lived effector cells (SLEC; CD127^−^KLRG1^+^) and MPEC (CD127^+^KLRG1^−^), further defined as LLMP cells (CD127^+^KLRG1^−^CD62L^+^). Approximately 70% of Mtb32A tetramer^+^CD8^+^ T cells in the lungs of BNT164-immunized groups had an MPEC phenotype; this proportion was significantly higher than those in BCG-immunized or negative control groups (~10%) (Fig. 6f). Percentages of LLMP cells were also increased in BNT164-immunized groups (Fig. 6i). Although the difference in frequencies of these cells (within the CD44^+^Mtb32A tetramer^+^CD8^+^ T cell population) was not statistically significant owing to low cell numbers in control groups (Fig. 6i), mean counts of Mtb32A tetramer^+^ LLMP cells ranged between 8 and 10 for the saline, BCG and NTL groups, compared to ~791 for BNT164a1 and ~760 for BNT64b1 (not depicted). This increase in MPEC and LLMP cells coincided with a significant decrease in SLEC frequencies (Extended Data Fig. 9i), although absolute numbers of these cells remained constant (not depicted). A trend of MPEC enrichment in lungs was also observed among CD44^+^Mtb32A tetramer^−^CD8^+^ T cells in BCG- and BNT164-immunized groups (Extended Data Fig. 9j).

In contrast to the lungs, mediastinal lymph nodes (mLNs) of BNT164-immunized animals did not show increased frequency of CD8^+^ T cells (Fig. 6g). A trend of enrichment of Mtb32a tetramer^+^ cells could be observed, but this was overall less pronounced than in lung tissues (Fig. 6h and Extended Data Fig. 9f). In the mLNs, the MPEC population was more abundant than in lungs for all groups but still enriched in BNT164-immunized animals (Extended Data Fig. 9k). Very low numbers of SLEC were detected in mLNs, and no enrichment of LLMP cells was observed (Extended Data Fig. 9l–m).

Taken together, the results of this comparative assessment of mLNs and lungs suggest that the antigen-specific CD8^+^ T cells induced by BNT164 candidates homed to the primary site of infection (lungs), exhibiting a memory precursor phenotype with hallmarks of long-lived immunity.

## Discussion

Here we describe two mRNA–LNP TB vaccine candidates, BNT164a1 and BNT164b1. Both candidates encode the same eight full-length *Mtb* antigens as fusion proteins on four mRNAs, which are successfully expressed in human cells. Prime-boost BNT164 immunization induced T cell and/or IgG responses against each of the eight encoded *Mtb* antigens, with expected differences in type and intensity of response between tested mouse strains. In low-dose aerosol *Mtb* challenge studies in C57BL/6 mice with two different *Mtb* strains, mice vaccinated with BNT164a1 or BNT164b1 had significantly lower bacterial load in lungs and spleens than those injected with saline.

Antigen selection for BNT164 candidates was driven by high-quality immunogenicity data in humans and, where available, efficacy signals in humans or NHP (Extended Data Table 1). To reduce murine bias in BNT164 immunogenicity testing, we used three different mouse models with varying MHC/HLA backgrounds. Antigens with limited T cell immunogenicity in one model, such as VapB47 in C57BL/6 and ESAT-6 in BALB/c, performed well in other models. Furthermore, antigens with low T cell immunogenicity in C57BL/6, such as Hrp1 and VapB47, induced IgG responses, confirming that they had been successfully expressed. Taken together, these results indicate that delivery and expression of BNT164 antigens was consistent in tested murine models and that differences in T cell immunogenicity were likely to have been driven by MHC/HLA differences. The data, therefore, support the inclusion of all eight antigens into the final BNT164 candidates aimed at diverse human HLA backgrounds. No IgG responses were detected against RpfD in our experiments, and antibodies against ESAT-6 were abrogated in the octavalent BNT164b1 candidate compared with the individual ESAT-6–RpfD mRNA cassette, suggesting antigen interference. This was considered to be acceptable, as ESAT-6 and RpfD were primarily included as T cell antigens^27^.

The only difference between BNT164 candidates was the mRNA platform used. Evidence suggests different adjuvant activity between these platforms: whereas uRNA is considered to signal through Toll-like receptors (TLR3, TLR7 and TLR8) and exhibits strong adjuvant activity, modRNA activates other innate immunity pathways (for example, melanoma differentiation-associated protein 5 signaling) and has lower adjuvant activity^23,25^. We detected higher levels of polyfunctional (IFNγ^+^TNF^+^IL-2^+^) and memory CD8^+^ cells in the BNT164a1 (uRNA) group compared with the BNT164b1 (modRNA) group. This was in alignment with previous findings in a B16-OVA murine melanoma model in which uRNA induced higher frequency of polyfunctional (granzyme B^+^IFNγ^+^TNF^+^) antigen-specific CD8^+^ T cells compared with modRNA, resulting in better tumor growth control^26^. However, we did not find consistent significant differences between modRNA- and uRNA-induced T cell responses via IFNγ ELISpot, nor in T cell phenotypes and protection following *Mtb* challenge. Platform differences between BNT164a1 and BNT164b1 may be underestimated in murine models owing to a generally lower sensitivity to innate immune signals compared with humans. Innate signaling pathways induced by mRNA–LNP are species-specific and remain to be fully elucidated^36^. For example, Tahtinen et al. recently showed that human immune cells respond to mRNA by producing inflammatory IL-1 cytokines, whereas mice produce markedly higher levels of anti-inflammatory IL-1ra^37^. NHP studies can be more informative for comparing mRNA platforms^38^, but even these higher-order models do not fully recapitulate human signaling pathways. Thus, whether the use of different mRNA platforms affects the efficacy of BNT164 candidates remains to be explored in humans.

The lack of established animal models for efficacy testing of TB vaccine candidates represents a hurdle in TB vaccine development. Many different TB preclinical challenge models can be explored, such as different mouse backgrounds or other rodent or NHP models; different dose, route, duration and timing of the challenge; or vaccination after BCG prime or *Mtb* exposure. However, none of these has been proven to predict human protection^7,39,40^. Therefore, we first selected the most widely used model in the field, a low-dose aerosol *Mtb* H37Rv challenge in C57BL/6 mice, and showed that BNT164a1 and BNT164b1 significantly reduced the bacterial load in lungs and spleen of infected mice compared with a saline control. In the second challenge study, we extended these findings and demonstrated protection against the hypervirulent L2 isolate HN878. In C57BL/6 mice, HN878 has been reported to induce TB immune responses that more closely resemble those in humans, such as formation of granulomas^41^, granuloma-associated lymphoid tissues^42^ and a protective role for IL-17 signaling^35^. BCG was included in our studies as a positive control but should not be used for efficacy comparisons. Despite the efficacy of BCG in mouse models and human infants, BCG revaccination does not protect against *Mtb* infection in adolescents and adults^43^. Rather, achieving similar lung CFU reductions with BCG, as previously reported^44^, helps to validate the fidelity of an experiment and allows cautious historical comparisons to other vaccine candidates^44^. Our preclinical efficacy data for BNT164 were consistent with findings for other vaccine candidates aimed at prevention of disease in adolescents and adults^44–46^. These include the M72 antigen adjuvanted with AS02 or AS01B, which provided approximately 0.6–0.7 log_10_ CFU reduction compared with saline or adjuvant-only controls in the same C57BL/6 challenge model^45^. No results of murine protection studies with clinical candidate M72/AS01E, which is currently being evaluated in a phase 3 trial (NCT06062238), are publicly available to our knowledge^44^. It is also important to consider some limitations of our challenge studies when interpreting the data. These include the lack of balanced host–pathogen interactions in mice, the lack of expression of hypoxia-associated (Hrp1 and VapB47) and resuscitation-associated (RpfA and RpfD) antigens in the investigated time frames, and the limited MHC repertoire of C57BL/6 mice. Therefore, BNT164 immunogenicity and efficacy in naive and Mycobacterial antigen-exposed individuals remain to be further determined in clinical trials.

Despite the limitations of the animal models utilized here, our findings provide encouraging indications of diverse and broad immune responses induced by BNT164 candidates. The M72/AS01E subunit vaccine candidate, an important vaccine candidate aimed at preventing TB in adolescents and adults, has shown efficacy in preventing TB disease in *Mtb*-exposed individuals (positive in the IFNγ release assay)^28,47^. M72/AS01E induced polyfunctional CD4^+^ T cells and high IgG responses, with CD8^+^ T cell responses detected in a phase 2 immunogenicity study^48^ but not in the phase 2b efficacy trial, possibly owing to the different time points investigated^28^. Although the mechanisms of protection of M72/AS01E remain to be elucidated, it is encouraging that BNT164 candidates, similar to M72/AS01E, induced strong IgG and CD4^+^ responses to M72 in mice, including polyfunctional CD4^+^ cells secreting IFNγ, TNF and IL-2 (ref. ^49^). Nonetheless, the expected efficacy of BNT164 candidates cannot be inferred from immunological comparisons with other vaccine candidates owing to a lack of correlates of protection and different modes of action. In addition to anti-M72 responses, BNT164 candidates elicited T cell and antibody responses to the other *Mtb* antigens included in the vaccine. Beyond M72, we have identified durable IgG antibody responses to recombinant Ag85A, Hrp1, RpfA, HbhA and VapB47. For the antigens for which IgG responses reduced over time, a third vaccine dose successfully boosted titers. Structural modeling suggested that BNT164-encoded chimeric antigens have the potential to fold correctly and thus to raise immune responses against native protein conformations. This was confirmed by ELISA with *Mtb* H37Rv lysate, which showed increased binding of serum IgG from BNT164-immunized mice after a third dose, possibly owing to increased affinity maturation. Whether these antibodies contribute to TB protection remains to be established.

One limitation of our work was that lung-resident immunity was investigated only in the context of infection. mRNA vaccines administered i.m. can confer protection against respiratory viral pathogens such as SARS-CoV-2 or RSV^18,19,50^. Although neutralizing antibodies are most relevant for protection against these viruses, lung-resident T cell responses have also been detected following mRNA vaccination, albeit to a limited extent and mostly at a lower magnitude compared to infection^51,52^. In our *Mtb* challenge study with HN878, we found evidence of BNT164-induced lymphocyte infiltration in the lung. This was driven by antigen-specific CD8^+^ T cells, indicating that systemically induced T cells have the capacity to migrate to the site of infection. Higher numbers of lymphocytes infiltrating the granulomas were correlated with lower bacterial burdens. Intracellular cytokine staining, after stimulation with antigens’ peptide pools, confirmed that these lung CD8^+^ cells were functional cytokine-secreting cells. Furthermore, the BNT164-induced CD8^+^ T cells exhibited memory precursor phenotypes both in the spleen in the immunogenicity study and in the lung after *Mtb* challenge, although more research is required to fully characterize lung-resident memory formation following BNT164 immunization. The contribution of CD8^+^ T cells to TB protection has not been fully elucidated, but recent data suggest important roles in the NHP model^53^ and indicate that the interplay between CD4^+^ and CD8^+^ T cells may be essential for optimal immunity in mice under specific conditions^54^. It remains to be established whether the BNT164-induced CD8^+^ T cells causally contribute to TB protection. Further investigation of the direct contribution of specific cellular or humoral responses in mice—for instance, by more comprehensive immune profiling, cellular depletion or adoptive transfer studies—would be of scientific interest. However, the results of such investigations may be of limited translational value until BNT164-induced immune responses have been characterized in humans, providing a benchmark for future investigation.

BNT164a1 and BNT164b1 were well tolerated in a GLP-compliant repeat-dose toxicity study in rats, with no vaccine-related systemic clinical signs or mortalities. The candidates induced transient dose-dependent inflammatory responses that could be attributed to vaccine-induced activation of the immune system: increased acute phase reactants, increased counts of white blood cells, transiently increased body temperature and microscopic inflammation at the injection site. The control group immunized with nontranslatable modRNA–LNP showed similar effects, indicating formulation-dependent activation of innate immunity rather than antigen-dependent inflammation. These results are consistent with those of SARS-CoV-2 mRNA–LNP vaccine studies, in which a similar inflammatory response was seen^55^.

In conclusion, preclinical data showed that TB vaccine candidates BNT164a1 and BNT164b1 are immunogenic, well tolerated and efficacious in rodent models, supporting their further clinical development. BNT164a1 and BNT164b1 have entered phase 1/2 clinical testing, where their safety and immunogenicity are being evaluated (NCT05537038, NCT05547464).

## Methods

### Study design

The objective of this study was to assess the immunogenicity, safety and efficacy of two mRNA vaccine candidates against TB in preclinical models. Statistical methods were not used to predetermine group sizes. Only animals with unobjectionable health status were selected for testing procedures, and humane endpoints were predefined. Animals were randomized only for the toxicity study. Investigators were not blinded to group identity during experiments or data analysis except in the pathology assessments. Each in vivo experiment was performed once. Group sizes, replicate numbers and statistical tests are specified in the corresponding Results sections.

### Animal husbandry and ethics

All mouse immunogenicity experiments were approved by the local animal welfare committee (Landesuntersuchungsamt Rheinland-Pfalz, protocol G 20-12-005) and conducted at BioNTech SE according to Federation of European Laboratory Animal Science Associations recommendations and in compliance with the German animal welfare act and Directive 2010/63/EU. Mice were kept under barrier and specific-pathogen-free conditions in individually ventilated cages (maximum five animals of one sex per cage) and under controlled environmental conditions (20–24 °C, 45–55% relative humidity, 75 air circulation per hour). Cages contained dust-free bedding made of debarked chopped aspen wood and additional nesting material. Autoclaved ssniff R/M-H food (ssniff Spezialdiäten GmbH) and autoclaved tap water were provided ad libitum and changed at least once weekly. Female C57BL/6JOlaHsd mice (Janvier Labs) and female BALB/c mice (Janvier Labs) were used at 8–12 weeks of age. HLA-A2.1/DR1 transgenic mice were acquired from INFRAFRONTIER (ID: EM:01783) and bred in-house; male and female mice were used at 20–24 weeks of age.

For the GLP-compliant toxicity study, Wistar Hannover rats (Charles River) were housed in a limited-access rodent facility at European Research Biology Center S.r.l. Italy (a test facility fully accredited by AAALAC) in clear polysulfone solid-bottomed cages (maximum five animals of one sex per cage) and under controlled environmental conditions (20–24 °C, 40–70% relative humidity, 15–20 air circulation per hour. Nesting material was provided inside suitable bedding bags and changed at least twice per week. A commercially available laboratory rodent diet (4 RF 21, Mucedola S.r.l.) and drinking water were offered ad libitum throughout the study, except before urine collection. Procedures and facilities were compliant with the requirements of Directive 2010/63/EU on the protection of animals used for scientific purposes. The national transposition of the Directive is defined in Decreto Legislativo 26/2014. Aspects of the protocol concerning animal welfare were approved by the local animal welfare body (Organismo preposto al benessere animale). Rats were used at 8–9 weeks of age and allocated to groups by computerized stratified randomization to give approximately equal initial group mean body weights.

For the challenge studies, female C57BL/6J mice (The Jackson Laboratory) were housed in specific-pathogen-free facilities at Trudeau Institute. For the H37Rv study, mice were vaccinated at 12–13 weeks of age. For the HN878 study, mice were vaccinated at 8–9 weeks of age. All mice were treated in accordance with the National Institutes of Health guidelines for housing and care of laboratory animals and the Institutional Animal Care and Use Committee guidelines at the Trudeau Institute under approved protocols 13-003.12 and 13-003.14. All efforts were made to minimize suffering and pain as described in the approved protocol.

### Protein structure modeling

Models of the BNT164 antigens were generated with AlphaFold2^30,56^ using MMseqs2 sequence alignment^57^ and PDB100 template mode. Five independent models were generated and compared for each antigen to identify areas of agreement and better understand modeling robustness. Predicted local distance difference test plots were generated via AlphaFold2 to depict model confidence. Using PyMOL (Schrodinger), the top-ranked models of BNT164 antigens were aligned with experimental structures of individual antigens (when available) or AlphaFold2 models of individual antigens from the AlphaFold Protein Structure Database^58^. Experimental structures were available for Ag85A (PDB 1SFR), Hrp1 (PDB 1XKF), PPE18 (the PPE domain from the closely related PPE15 (PDB 5XFS) was used) and ESAT-6 (PDB 3FAV). r.m.s.d. values for the alignments were calculated in PyMOL.

### Cell culture

HEK293T cells (ECACC catalogue number 12022001) were grown in Dulbecco′s modified Eagle medium (DMEM, high glucose, GlutaMAX, pyruvate; Gibco) supplemented with 10% nonheat-inactivated fetal bovine serum (FBS) Superior (Sigma-Aldrich).

### RNA design and production

BNT164a1 and BNT164b1 contain four mRNAs, with each mRNA encoding a fusion of two *Mtb* antigens. A human HLA-A-derived signal peptide was added to the N terminus of each fusion antigen to facilitate secretion^59^, and no linkers were introduced between antigens. This resulted in four fusion proteins of different amino acid sizes to allow the possibility of size-based RNA analytics: Ag85A–Hrp1 (465 amino acids), ESAT-6–RpfD (273 amino acids), RpfA–HbhA (630 amino acids) and M72–VapB47 (847 amino acids). Antigen sequences were derived from the H37Rv *Mtb* strain; exact amino acids included are listed in Extended Data Table 1. M72 contained an N-terminal HIS-tag only in the construct used for the immunogenicity studies in C57BL/6 mice depicted in Figs. 2b,c and 3a and Supplementary Figs. 1a and 2 and was subsequently removed.

BNT164a1 and BNT164b1 DNA templates were cloned into a plasmid vector with backbone sequence elements as described previously^16^. The DNA was purified and spectrophotometrically quantified. The four DNA templates were mixed and transcribed in vitro with T7 RNA polymerase in the presence of a trinucleotide Cap1 analog ((m27,3′-O)Gppp(m2′-O)ApG) (TriLink). For BNT164b1, *N*^1^-methylpseudouridine-5′-triphosphate (Thermo Fisher Scientific) was used instead of uridine-5′-triphosphate. RNA was purified using magnetic particles. RNA integrity was assessed by microfluidic capillary electrophoresis (Agilent Fragment Analyzer), and the concentration, pH, osmolality, endotoxin level and bioburden of the solution were determined.

The DNA template sequences used to generate the RNA-encoded fusion antigens are listed in Supplementary Table 1.

### RNA formulation

Purified RNA was formulated into lipid nanoparticles as described previously (Acuitas Therapeutics Inc)^16^. The lipid nanoparticle contained RNA, an ionizable lipid, ((4-hydroxybutyl)azanediyl)bis(hexane-6,1-diyl)bis(2-hexyldecanoate)), a PEGylated lipid, 2-[(polyethylene glycol)-2000]-*N*,*N*-ditetradecylacetamide and two structural lipids (1,2-distearoyl-sn-glycero-3-phosphocholine and cholesterol).

### Cell transfection

HEK293T cells at 80–90% confluency were seeded in 12-well plates at 2 × 10^5^ cells per well 1 day before transfection. HEK293T cells were transfected with uRNA or modRNA constructs (0.25 μg for single mRNAs or 1 μg for the mix of all four mRNAs) using a RiboJuice mRNA Transfection Kit (Sigma-Aldrich) according to the manufacturer’s instructions. Briefly, mRNA was diluted in OptiMEM and mixed with mRNA boost reagent and RiboJuice mRNA reagent. The transfection mixture was incubated for 4 min at room temperature (RT) and added dropwise to the cells. The plate was gently mixed and incubated overnight (18 h) at 37 °C, 5% CO_2_, in a humidified atmosphere. Nontreated control wells were run in parallel (nontransfected cells).

### Cell lysis and total protein isolation

Transfection media were discarded from transfected cells, and the cells were rinsed with Dulbecco’s phosphate-buffered saline (DPBS) and centrifuged at 300*g* for 5 min at 4 °C. The supernatant was discarded, and 100 μl radioimmunoprecipitation assay buffer with 0.1% SDS containing a protease inhibitor cocktail (one cOmplete ULTRA) was added to the tube, followed by incubation on ice for 10 min to lyse cells. The tube was centrifuged at ~17,000*g* for 10 min at RT, and the supernatant containing the total protein suspension was kept on ice and used immediately for determination of protein concentration by western blot or stored at −80 °C.

### Western blot

Total protein extracts from transfected and nontransfected control HEK293T cells were quantified with a Pierce BCA protein assay kit (Thermo Scientific), according to the manufacturer’s protocol. Total protein extract and recombinant protein controls (produced in-house in an *Escherichia coli* BL21 (DE3) expression system) were denatured under reducing conditions (95 °C) and subjected to SDS–PAGE. Then, 10 μg total protein per well or prestained protein marker (3 μl PageRuler Plus; Thermo Fisher Scientific) was loaded onto an 8–16% polyacrylamide gradient gel (Mini PROTEAN TGX; Bio-Rad) and run at 140 V for ~40 min. Proteins were transferred to a nitrocellulose membrane using a semidry method (Trans-Blot Turbo Transfer System, Bio-Rad). Blotted proteins were detected with primary antibodies: mouse anti-ESAT-6 (clone 11G4, Abcam ab26246, 1:2,000), rabbit polyclonal anti-Hrp1 (BEI NR-36512, 1:2,000), rabbit anti-tubulin (clone 11H10, Cell Signaling Technology 9099S, 1:1,000); and custom-made (Squarix GmbH) mouse anti-RpfD (clone 16C8-1C5, 1:2,000), mouse anti-RpfA (clone 8C3-1C4, 1:500), mouse anti-HbhA (clone 3A4-1G8, 1:500), mouse anti-M72 (clone 10G8-1C1, 1:500), mouse anti-VapB47 (clone 4D10-1C3, 1:2,000) and mouse anti-Ag85A (clone 6G11, nonpurified hybridoma supernatant, 1:500). Custom-made primary antibodies were raised in BALB/c mice following repeated s.c. immunization with individual recombinant proteins mixed with Freund’s incomplete adjuvant and isolated using hybridoma technology. For detection, secondary polyclonal antibodies coupled to HRP were used (anti-mouse IgG, Jackson ImmunoResearch Laboratories catalogue number 115-035-071, 1:10,000; anti-rabbit IgG, Sigma-Aldrich A0545, 1:2,000). Blots were developed with chemiluminescent substrate (Cytiva Amersham ECL Prime Western Blotting Detection Reagent) and analyzed with a ChemiDoc Imaging System and ImageLab software (Bio-Rad).

### Immunizations

For immunization studies, the mice were anesthetized by inhalation of 2.5% isoflurane in oxygen, and the injection site on the hind leg of the mouse was shaved. The mRNA test items (at a dose of 4 μg for BNT164 and 1 μg for individual mRNA cassettes) or saline were injected i.m. into the gastrocnemius muscle in a volume of 20 μl per injection with a 30-gauge-needle insulin syringe. BCG (10^6^ CFUs) was administered s.c. without previous anesthesia. Injection sites were observed every 24 h for 2 days postinjection and at least once weekly throughout the study.

For the toxicity study, rats received i.m. injections of the mRNA test items or saline into the quadriceps muscle (alternating the right and left thigh) with a 30-gauge-needle insulin syringe, at the following dose volumes: 60 μl saline, 30 μg per 60 μl NTL, 10 μg per 20 μl or 30 μg per 60 μl BNT164b1, or 10 μg per 20 μl BNT164a1. Before the first administration, and as necessary during the course of the study, the thigh was shaved. Daily examination of the injection site was performed predose and approximately 4 h and 24 h after each administration.

For the challenge study, mice were immunized i.m. in the gastrocnemius muscle with 4 μg of mRNA test items or saline in a volume of 20 μl per injection using a 30-gauge needle. BCG-containing saline (10^6^ CFU) was injected s.c. either at the nape of the neck or around the hip using a 26-gauge needle. Injection sites were monitored weekly or on consecutive days following injection.

### Animal monitoring

Routine animal monitoring was carried out daily, including inspection for dead mice and control of food and water supplies. The health of each mouse was closely assessed at least once weekly, and the results were documented in health monitoring sheets. The general physical condition of the mice was assessed according to the following parameters: mice were observed daily and weighed at least weekly for the duration of the study. Animals that lost more than 20% body weight were euthanized immediately. Mice were observed for the following clinical signs for the duration of the study: (1) signs of moderate toxicity (intermittent hunching, ruffled coat, intermittent abnormal breathing, or intermittent tremors and convulsions); and (2) signs of substantial toxicity (prolonged immobility, hunching, dehydration, skin tenting, labored breathing or prolonged prostration). Animals exhibiting signs of substantial toxicity were euthanized immediately. During the immunization study in HLA-A2.1/DR1 transgenic mice, one animal in the BNT164a1 group was euthanized owing to health issues unrelated to treatment and was not included in the analysis. In the antigen interference experiment, one mouse in the group immunized with the ESAT-6–RpfD mRNA cassette died during the blood draw on day 28. During the *Mtb* H37Rv challenge study, one animal in the BNT164a1 group was euthanized owing to severe alopecia and erythema (>2.0 cm) at the injection site after the second immunization, and one animal in BNT164b1 group was euthanized owing to an unrelated event. In the *Mtb* HN878 challenge study, one mouse immunized with BCG was euthanized with symptoms of a possible ear infection.

In the toxicity study, rats were checked at least once daily for mortality and clinical signs. Each animal was weighed on the day of allocation to vaccination group, on the day of administration, 24 h and 48 h after each administration, and just before necropsy. During recovery, animals were weighed at weekly intervals. Water consumption for each cage was recorded daily, and food consumption was recorded twice weekly. Rectal body temperature was measured on days 1, 8, 15 and 22 predose and at approximately 4 h and 24 h postdose (days 2, 9, 16 and 23). In cases of recorded postdose rectal body temperature ≥38.5 °C, the temperature was measured the day after (48 h postdose) until the values returned below 38.5 °C.

### Blood collection and serum isolation

In the immunization studies, blood was sampled either from the facial vein using a lancet (without previous anesthesia), or from the retro-orbital venous sinus using a 29-guage glass capillary (upon isoflurane-induced anesthesia). Blood was collected into an appropriate plastic tube (Sarstedt, Z-gel included for clotting activation). Blood samples were centrifuged for 5 min at RT and 10,000*g* for collection of serum, which was then stored at −20 °C.

In the toxicity study in rats, blood samples for clinical pathology investigations were collected under conditions of overnight food deprivation from the retro-orbital sinus on day 3 or from the abdominal vena cava on days 24 and 43. Blood samples were collected into tubes as follows: EDTA anticoagulant for hematological investigations; citrate anticoagulant for coagulation tests (fibrinogen); without anticoagulant for biochemical tests, α2-macroglobulin, and α1-acid-glycoprotein.

### Euthanasia procedure and organ collection

Mice were euthanized by cervical dislocation or by exposure to CO_2_. Rats were euthanized by exsanguination under isoflurane anesthesia, and necropsy was supervised by a pathologist. A detailed post mortem examination was conducted, observations were noted, the requisite organs were weighed, and the required tissue samples were preserved in fixative (10% neutral buffered formalin) and processed for histopathological examination.

### Splenocyte preparation

Spleens were collected and stored in DPBS on ice. Single-cell suspensions were prepared by homogenization through 70-μm cell meshes using the plunger of a syringe. Splenocytes were washed with an excess volume of DPBS followed by centrifugation at 300*g* for 6 min at RT, and the supernatants were discarded. Erythrocytes were lysed with erythrocyte lysis buffer (154 mM NH_4_Cl, 10 mM KHCO_3_, 0.1 mM EDTA) for 5 min at RT. The reaction was stopped with an excess volume of DPBS. After another washing step, cells were resuspended in complete RPMI medium (10% FBS, 1% nonessential amino acids (NEAA), 1% sodium pyruvate, 0.5% penicillin/streptomycin), passed through a 70-μm cell mesh again, counted and stored short-term at 4 °C for use on the same day or frozen in Cryomedium (FBS nonheat-inactivated + 10% dimethyl sulfoxide cell culture grade).

### Sorting of splenocytes

CD4^+^ and CD8^+^ T cells were sorted from single-cell suspensions of splenocytes by magnetic-activated cell sorting (Miltenyi Biotec). Before cell sorting, splenocytes were pooled from all five mice in each group. Mouse CD4^+^ and CD8^+^ T cell isolation kits, a QuadroMACS Separator and LS columns were used, following the manufacturer’s instructions. A negative isolation method was used; this resulted in elution of untouched CD4^+^ and CD8^+^ T cells. Cells were separately centrifuged and prepared at a concentration of 1 × 10^6^ cells ml^−1^ in RPMI 1640 medium supplemented with 10% heat-inactivated FBS, 1% NEAA, 1% sodium pyruvate, 1% HEPES, 0.5% penicillin/streptomycin and 50 μM 2-mercapthoethanol.

### Preparation of bone-marrow-derived dendritic cells

For analysis of the sorted CD4^+^ and CD8^+^ T cells via ELISpot, we used bone-marrow-derived dendritic cells (BMDCs) as antigen-presenting cells. BMDCs were differentiated from the corresponding mouse models by stimulation of bone marrow cells with GM-CSF (1,000 U ml^−1^) for 7 days. Dendritic cell identity was confirmed by expression of CD11c, CD86 and MHC-II via flow cytometry.

### ELISpot

ELISpot assays were performed according to the manufacturer’s protocol (with minor modifications as described below) using a mouse IFNγ ELISpot^PLUS^ kit (Mabtech). Briefly, 96-well ELISpot plates precoated with IFNγ antibody were washed with PBS and blocked with serum-free medium for at least 30 min at RT. The medium was removed, and cells were added to the plates: either 5 × 10^5^ total splenocytes per well (100 μl), or 1 × 10^5^ sorted CD4^+^CD8^+^ T cells per well (100 μl) together with 5 × 10^4^ BMDCs per well (50 μl). BMDCs served as antigen-presenting cells for sorted T cells. Another 100 μl of overlapping peptide pools (15-mer-long peptides with 11 amino acid overlap) were added to the wells at a final concentration of 2 μg ml^−1^: Ag85A (34 overlapping peptides; Pepscan), M72 (180 overlapping peptides; Pepscan), ESAT-6 (21 overlapping peptides; Pepscan), HbhA (47 overlapping; Pepscan), RpfD (36 overlapping peptides; Pepscan), Hrp1 (33 overlapping peptides; JPT Peptide Technologies), RpfA (99 overlapping peptides; Pepscan), VapB47 (22 overlapping peptides; Pepscan). Purified protein derivative (AJ Vaccines) was used at 10 μg ml^−1^. As a positive control, splenocytes were stimulated with 2 μg ml^−1^ concanavalin A (Sigma); for the unstimulated control, only medium was added. TRP1 (for C57BL/6JOlaHsd mice), AH1 (for BALB/c mice) or CMVpp65 (for HLA-A2.1/DR1 transgenic mice) (all JPT Peptide Technologies) were used as nonspecific stimulants at 2 μg ml^−1^. Plates were incubated overnight in a humidified incubator with 5% CO_2_ at 37 °C for 18 h. Next, a detection antibody, streptavidin-ALP, and ready-to-use substrate were added to the wells according to the manufacturer’s protocol. After plates had been dried for 2–3 h under laminar flow or overnight, we used an ELISpot plate reader (ImmunoSpot S6 Core Analyzer, CTL) to count and analyze spot numbers per well.

### T cell phenotyping of noninfected animals via flow cytometry

First, 5 × 10^5^ fresh splenocytes in 100 μl RPMI 1640 medium supplemented with 10% heat-inactivated FBS, 1% NEAA, 1% sodium pyruvate, 1% HEPES, 0.5% penicillin/streptomycin and 50 μM 2-mercapthoethanol were transferred to 96-well U-bottomed cell culture plates. Then, 100 μl of an overlapping peptide mix containing all eight *Mtb* antigens (as specified above) were added at 1 μg ml^−1^ per peptide in the presence of costimulatory antibodies (1 μg ml^−1^ anti-CD28, eBioscience catalogue number 16-0281-38, clone 37.51; and 1 μg ml^−1^ CD49d, BioLegend catalogue number 103629, clone R1-2). As a nonstimulation control, medium only was added to detect unspecific background signals. Plates were incubated for 1 h in a 37 °C humidified incubator with 5% CO_2_ before addition of GolgiStop + GolgiPlug (BD Biosciences). After another 4–5-h incubation, the plates were stored at 4 °C overnight. Cells were then harvested, transferred to a 96-well U-bottomed plate for flow cytometry, centrifuged (360*g*, 5 min, 4 °C), resuspended in 100 μl per well 1× permeabilization buffer (eBiosciences) and incubated for 5 min at 4 °C. After another centrifugation (400*g*, 3 min, 4 °C), the supernatant was discarded, and the cells were stained for 20 min at 4 °C using the following panel: Live/Dead-APC-R700 (1:1,000, BD Biosciences, catalogue number 564997), CD3-BV605 (1:200, clone 145-2C11, BioLegend catalogue number 100351), CD4-BV510 (1:400, clone RM4-5, BioLegend catalogue number 100559), CD8-APC (1:200, clone 53-6.7, BD Biosciences catalogue number 553035), IFNγ-PE-Cy7 (1:500, clone XMG1.2, BioLegend catalogue number 505826), IL-2-BV711 (1:100, clone JES6-5H4, BioLegend catalogue number 503837), TNF-Alexa488 (1:100, clone MP6-XT22, BioLegend catalogue number 506313), CD127-PE (1:200, clone SB/199, BioLegend catalogue number 121111), KLRG1-APC_eFluor780 (1:100, clone 2F1, Thermo Fisher, catalogue number 47-5893-82) and CD62L-BV785 (1:200, clone MEL-14, BioLegend catalogue number 104440). After the staining procedure, cells were washed twice in permeabilization buffer and resuspended in 150 μl FACS buffer (PBS + 0.1% bovine serum albumin) for flow cytometry analysis using FACSCelesta (BD).

### T cell phenotyping via flow cytometry of *Mtb* HN878-infected animals

Lung tissue was prepared by coarse chopping and enzymatic digestion of the tissue in a 0.5 mg ml^−1^ solution of collagenase (Roche) and DNase (Sigma-Aldrich) and incubated for 30–45 min at 37 °C. Single-cell suspensions were prepared from digested lung tissue or mLNs harvested into HBSS by dispersal through a 70-μm nylon tissue strainer (BD Falcon). The cell suspensions were treated with Gey’s solution to remove erythrocytes. Lymphocytes were enriched from lung tissue by differential centrifugation, using a gradient of 40%/80% Percoll (GE Healthcare).

Lymphocytes were transferred to a 96-well plate, at a concentration of 1.5 to 2 × 10^6^ cells per well. After the lymphocytes had been incubated with Fc block (BioLegend) for 15 min on ice, they were stained with tetramer reagents (PE-labeled ESAT-6_1__–20_ I-A^b^ tetramer (MBL Life Science, product code MBL-TS-M707-1) and APC-labeled Mtb32A(PepA)_309__–318_ H-2D^b^-tetramer (produced at Trudeau Institute) for 60 min at RT and with a surface antibody panel for 30 min on ice. The surface antibody panel consisted of CXCR5-FITC (clone L138D7, BioLegend catalogue number 145520, 1:100); CD127-PE-CF594 (clone SB/199, BD catalogue number 562419, 1:100); KLRG1-PerCP-Cy5.5 (clone 2F1/KLRG1, BD catalogue number 563595, 1:100); PD1-Pe-Cy7 (clone RMP1-30, BioLegend catalogue number 109110, 1:100); CD4-BV421 (clone RM4-5, BioLegend catalogue number 100544; 1:100); CD8-BV510 (clone 53-6.7, BioLegend catalogue number 100751; 1:800); CD62L-AF700 (clone MEL-14; BioLegend catalogue number 104426 1:400); CD44.APC-eFluor780 (clone IM7; Invitrogen catalogue number 47-0441-82 1:400) and Zombie UV Live/Dead stain (BioLegend catalogue number 77474).

For intracellular cytokine staining, lung lymphocytes were stimulated with two different peptide pools containing 2 μg ml^−1^ per peptide in complete Dulbecco′s modified Eagle medium, containing 10% fetal calf serum. One pool covered antigens M72, VapB47 and Ag85, with a total size of 1,117 amino acids. The other pool covered Hrp1, ESAT-6, HbhA, RpfA and RpfD, with a total size of 1,172 amino acids. After 1.5 h of stimulation, Brefeldin A was added at 50 μg ml^−1^ (BioLegend), followed by incubation for another 3.5 h at 37 °C. The cells were then incubated with Fc block (BioLegend) and Live/Dead stain (Zombie UV viability dye; BioLegend catalogue number 423107; 1:500) on ice for 15 min, followed by surface staining for 30 min on ice with CD4-BV421 (clone RM4-5, BioLegend catalogue number 100544; 1:100) and CD8-BV510 (clone 53-6.7, BioLegend catalogue number 100751; 1:800). Cells were fixed and permeabilized on ice for 30 min using a transcription factor buffer set (BD Pharmingen) before being stained with anti-IFNγ-APC (clone XMG1.2, BD Pharmingen catalogue number 554413; 1:100) and anti-TNF-AF488 (clone MP6-XT22, Invitrogen catalogue number 53-7321-81; 1:100) for 30 min on ice.

Surface or intracellularly stained cells were fixed in 2% paraformaldehyde for 24 h before being analyzed on a LSRII flow cytometer (BD Biosciences). One lung sample from group BNT164b1 was excluded owing to a staining problem. Three MLN samples (one each from the saline, BCG and BNT164b1 groups) and the CD4 analysis of an additional BNT164b1 sample were excluded owing to CD4^+^CD8^+^ double-positive populations, likely caused by thymic contamination. Flow cytometry data were analyzed using FlowJo v.10.8.1.

### ELISA

Serum samples were tested in 96-well plates to determine their antigen-specific antibody concentrations. Each well of a MaxiSorp plate was coated with 100 ng per 100 μl per well of recombinant *Mtb* proteins produced in *E. coli* or mouse IgG isotype control. For lysate ELISA, 1 μg per 100 μl of *Mtb* H37Rv lysate (BEI resources NR-14822) was used instead. Plates were incubated at 4 °C overnight. Wells were washed with PBS with 0.01% Tween 20 and blocked with casein blocking buffer (Sigma-Aldrich) at 37 °C for 1 h on a shaker. After another round of washing, serially diluted serum samples in blocking buffer or negative control (blocking buffer alone) were added in duplicate, followed by incubation at 37 °C for 1 h on a shaker. After the plates had been washed, HRP-conjugated goat polyclonal anti-mouse IgG secondary antibody was added (Jackson ImmunoResearch 115-035-071, 1:7500 in blocking buffer), and plates were incubated at 37 °C for 45 min on a shaker. The plates were washed again; then, TMB substrate (Biotrend Chemikalien GmbH) was added, followed by incubation for 8 min at RT in the dark. The reaction was stopped with 100 μl 25% sulfuric acid, and the absorbance (450 nm, ref. 620 nm) was measured on an Epoch microplate reader (BioTek). The mean change in optical density measured from the absorbance values at 450 nm and 620 nm for each sample was calculated. IgG concentrations were quantified by interpolation from the IgG isotype standard curve in GraphPad Prism 9 or Genedata Screener.

### Toxicity study blood analysis

Hematological parameters (for example, white blood cells and large unstained cells) were measured using a Siemens Advia 120. Fibrinogen values were determined with an Instrumentation Laboratory ACL Elite PRO. α2-macroglobulin and α1-acid-glycoprotein were analyzed using validated commercial ELISA kits. Some samples were unsuitable for hematological analysis: 2 animals each from the saline group, BNT164 NTL group and BNT164b1 30 μg ml^−1^ group on day 3; 2 animals from the saline group, 1 animal from the BNT164 NTL group and 1 animal from the BNT164b1 10 μg ml^−1^ group on day 24; and 1 animal from the saline group on day 43.

### Challenge studies

C57BL/6J mice between 12 and 13 weeks of age were immunized i.m. with mRNA test items as follows. BNT164a1, BNT164b1 and NTL were given twice (days 0 and 21). A comparison group was given 10^6^ CFUs of BCG s.c. once (day 0). On day 56, animals were challenged with low-dose (~100 CFU) aerosol *Mtb* (strain H37Rv or HN878) using a Glas-Col airborne infection system. Stocks of *Mtb* strains used in the aerosol infection were cultured in Proskauer Beck medium containing 0.05% Tween 80 to mid-log phase and frozen in 1-ml aliquots at −80 °C. Animals were euthanized either 30 or 60 days postchallenge (days 87 and 117 postvaccination), and left lung lobes and spleens were harvested and homogenized in 4.5 ml of normal saline using a glass tube and pestle (Glas-Col). Organ homogenates were serially diluted 1:10, and 100 μl of each dilution step were plated on 7H11 agar on a 100 mm petri dish. Agar plates were incubated for 18 days at 37 °C, 6% CO_2_, at 60% humidity. After the indicated incubation time, bacterial colonies were counted, and the total bacterial burden in tissue homogenate was calculated and recorded as log_10_ CFU.

### Histopathological examinations

For rat samples, fixed and preserved tissues were dehydrated, embedded in paraffin wax, cut into 5-μm-thick sections, and stained with hematoxylin and eosin.

For mouse samples, right lung lobes were inflated with 0.5–0.7 ml neutral buffered formalin, placed in histology cassettes (Fisher Scientific catalogue number 15-182-706) and submerged in 10% neutral buffered formalin for 48 h. The fixed tissues were processed using Tissue-Tek VIP 3000 (Sakura) and embedded in paraffin wax (Fisher Scientific 22900701) using a Tissue-Tek embedding station. Once set, the blocks were sectioned at 5 μm using a Leica RM2125RT microtome and placed on slides (SuperFrost Plus; Fisher Scientific 1255015). After drying, a representative slide from each mouse was heated for 20 min on a slide-warming plate to adhere the tissues to the slides. Following deparaffinization and rehydration, the slides were stained with hematoxylin (Epredia) and eosin (Epredia) or submitted to acid-fast staining using an Acid-Fast Stain Kit (ENG Scientific 6500) following the manufacturer’s protocol. The slides were coverslipped using EcoMount (BioCare Medical, EM897L) and 22 mm × 22 mm coverslips (Fisher Scientific, 12541016). Full slide scans were taken with a ×40 objective using a Leica Aperio AT2 Slide Scanner. Granuloma burden per lung section was quantified by a pathologist blinded to the group identities using the following scoring system: grade 0, no granulomas observed; grade 1: minimal (<5 granulomas); grade 2, mild (5–9 granulomas); grade 3, moderate (10–14 granulomas); grade 4, marked (15–20 granulomas); grade 5, severe (>20 granulomas). The cellular composition of granulomas was further classified by assigning a rounded percentage of the infiltrate or aggregates contributed by macrophages, lymphocytes or neutrophils (0, 25, 50, 75 or 100%); these percentages were multiplied by the granuloma score to obtain an individual score for each cell type. Using acid-fast-stained slides, numbers of discrete granulomas containing acid-fast, rod-shaped bacteria, consistent with *Mtb* morphology, were counted. Percentages of granulomas containing acid-fast bacteria were calculated based on the granuloma count.

### Statistical analysis

GraphPad Prism 9 software was used for generation of graphs and for statistical analysis. Flow cytometry and ELISpot (complete splenocytes) data were analyzed by one-way analysis of variance (ANOVA) with Tukey’s multiple comparisons test. *Mtb* lysate ELISA data were analyzed by ratio-paired *t*-tests corrected for multiple comparisons by false discovery rate calculation using Benjamini, Krieger, Yekutieli with a threshold of q < 0.05. Colony counts and bacterial burden assessment via pathology in challenge studies were analyzed by one-way ANOVA with Dunnett’s multiple comparison test. Immune cell quantification and phenotyping data in challenge studies were analyzed by Kruskal–Wallis test followed by Dunn’s multiple comparison test. An alpha significance level of 0.05 was set as the threshold for *P* values (95% confidence intervals). Statistical significance was reported as follows: *P < 0.05, **P < 0.01, ***P < 0.001, ****P < 0.0001.

### Reporting summary

Further information on research design is available in the Nature Portfolio Reporting Summary linked to this article.

## Online content

Any methods, additional references, Nature Portfolio reporting summaries, source data, extended data, supplementary information, acknowledgements, peer review information; details of author contributions and competing interests; and statements of data and code availability are available at 10.1038/s41590-026-02545-z.

## Supplementary information

Supplementary Information Supplementary Figs. 1–4.

Reporting Summary

Peer Review File

Supplementary Data Table 1 DNA sequences encoding the BNT164a1 and BNT164b1 fusion antigen-encoding RNAs.

## Source data

Source Data Fig. 1 Uncropped western blots used to generate Fig. 1.

## Data Availability

All data associated with this study can be found in the main text or the Supplementary Information. Source data are provided with this paper.

## Extended data

**Extended Data Table 1:** *Mtb* antigens included in BNT164 vaccine candidates

Antigen^a^	Characteristics^a^	AAs in BNT164	Immunogenicity and efficacy
Ag85A (Rv3804c)	Has mycolyltransferase activity; essential for cell wall integrity; secreted protein.	42–338	Human: Humoral, CD4^+^ and CD8^+^ T-cell responses after boost immunization of healthy BCG vaccinated adults^60^. Mouse: Immunogenicity and protection against TB reported from DNA vaccine^61^. **NHP: Multiantigen cytomegalovirus-based vaccine was highly efficacious and even resulted in sterilizing immunity in a subgroup of animals^27^.
ESAT-6 (Rv3875)	Essential *Mtb* virulence factor; part of RD1 loci of *Mtb*; abundantly secreted at early stages of infection; secreted protein.	2–95	Human: CAF01-adjuvanted Ag85B/ESAT-6 fusion protein vaccination induced long-lasting CD4^+^ T-cell response^62^. ESAT-6 is one of the components of IGRA test used to diagnose latently TB infected individuals. Mouse: Immunogenicity and protection against TB reported^63^. Deletion of ESAT-6 and CFP10 from MTBVAC (*Mtb* live-attenuated vaccine candidate) reduced the protection of MTBVAC^64^. **NHP: see above.
M72 (Rv0125 and Rv1196)	Fusion protein consisting of two antigens^45^: Mtb32A (PepA): probable serine protease involved in metabolism and respiration; detected in early stage of infection; secreted protein. Mtb39a (PPE18): PE/PPE family protein of unknown function; detected in late stages of infection; probable membrane-bound protein.	Similarly fused as described before^65^	Human: Immunization led to early onset humoral response and CD4^+^ T-cell responses, sustained for 3 years; 49.7% (95% CI: 2.1 to 74.2) vaccine efficacy observed 3 years after immunization in phase II trial^28^. Mouse: Immunogenicity and protection against TB reported^45^.
VapB47 (Rv3407)	Possible antitoxin; virulence associated; intracellular protein.	2–99	Human: VapB47-specific IFNγ responses have been observed upon restimulating active TB patient blood cells^66^. Mouse: Immunogenicity and protection against TB reported when delivered as a DNA vaccine booster to BCG immunization^67^. **NHP: see above.
Hrp1 (Rv2626)	Dormancy associated; expression induced by oxygen/nutrient stress; detected in culture as well as membrane-bound.	2–143	Human: Hrp1-specific antibody detected in active TB patients^68^. Mouse: Immunogenicity and protection against TB reported. Plasmid DNA vaccination induced strong humoral and Th1-type cellular response^69^. Long-term protection from TB when given together with ESAT6-Ag85B-MPT64_(190–198)_-Mtb8.4^70^. **NHP: see above.
RpfD (Rv2389c)	Probable role in promoting the resuscitation.	2–154	Human: CD4^+^ and CD8^+^ T-cell responses observed in LTBI and active TB patients^71^. IFNγ production by T cells in response to RpfD and RpfA is higher in LTBI than in pulmonary TB^72^. Mouse: Immunogenicity observed after *Mtb* infection and BCG vaccination. Moderate protection from TB was induced by immunization with a family member (RpfB)^73^. **NHP: see above.
RpfA (Rv0867c)	Probable role in promoting the resuscitation; detected in late stage of infection; secreted protein.	2–407	Similar to RpfD.
HbhA (Rv0475)	Required for extrapulmonary dissemination of *Mtb*; membrane-bound.	2–199	Human: T-cell response against HbhA reported from LTBI^74^. Mouse: Immunogenicity and protection against TB reported^75^.

^a^Data source: https://mycobrowser.epfl.ch/

BCG = Bacillus Calmette-Guérin; IFNg = interferon gamma; IGRA = IFN Gamma Release Assay; LTBI = latent tuberculosis infection; *Mtb* = *Mycobacterium tuberculosis*; NHP = non-human primate; Tb = tuberculosi.
