# Supplementary material for: mRNA-based tuberculosis vaccines BNT164a1 and BNT164b1 are immunogenic, well tolerated and efficacious in rodent models
Source: Nat Immunol. 2026 Jun 12;27(8):1653–65. doi: 10.1038/s41590-026-02545-z (PMC13414557; doi:10.1038/s41590-026-02545-z)
Supplement: Supplementary file 1 — Supplementary Figs. 1–4. [file 41590_2026_2545_MOESM1_ESM.pdf]

nature immunology

Supplementary information https://doi.org/10.1038/s41590-026-02545-z

mRNA-based tuberculosis vaccines BNT164a1 and BNT164b1 are immunogenic, well tolerated and efficacious in rodent models

In the format provided by the authors and unedited

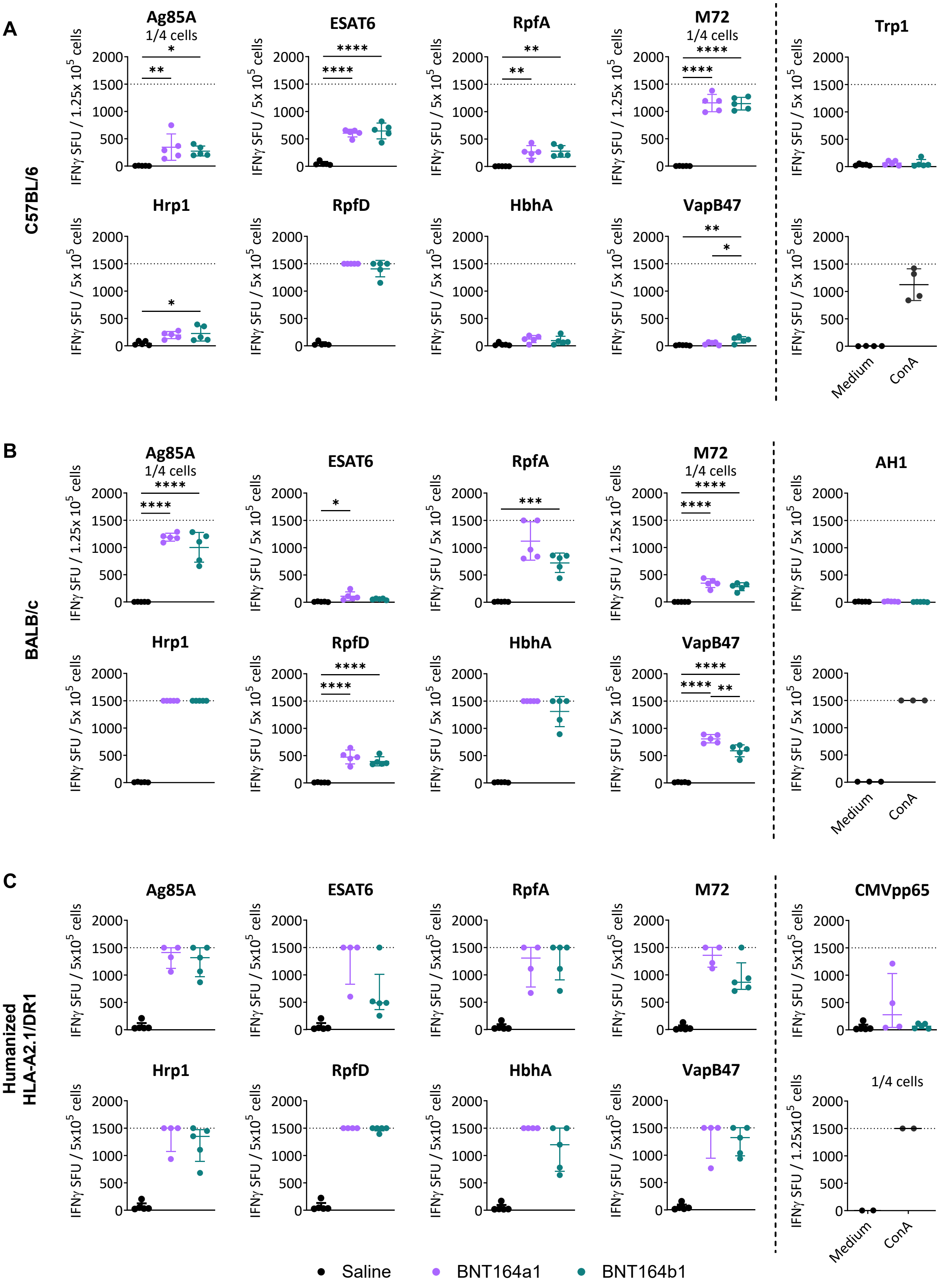

Supplementary Fig.1. BNT164 candidates induced antigen-specific total T-cell responses in different mouse strains

(A) C57BL/6, **(B)** BALB/c, and **(C)** HLA-A2.1/DR1 mice received IM injection of 4 µg BNT164a1, 4 µg BNT164b1, or saline control on Days 0 and 21. Splenocytes were isolated on Day 42 and stimulated with antigen-specific peptide pools, medium only, concanavalin A (con A), or non-specific peptides (TRP1, AH1, CMVpp65). Responses were assessed by IFNγ ELISpot assay after ~18 hours incubation. Group mean values are indicated by horizontal bars (± standard deviations), means from individual mice (n=4-5/group, measured in duplicates) are depicted as circles. For stimulation with medium or concanavalin A, technical replicates are shown. One-way ANOVA with Tukey's multiple comparisons test was performed**;** * = $p < 0.05$; ** = $p < 0.01$; *** = $p < 0.001$; **** = $p < 0.0001$. SFUs for some samples reached the upper limit in number of spots that can be correctly counted (indicated with dashed horizontal line) and were not statistically analyzed). IM = intramuscular; SFU = spot-forming unit.

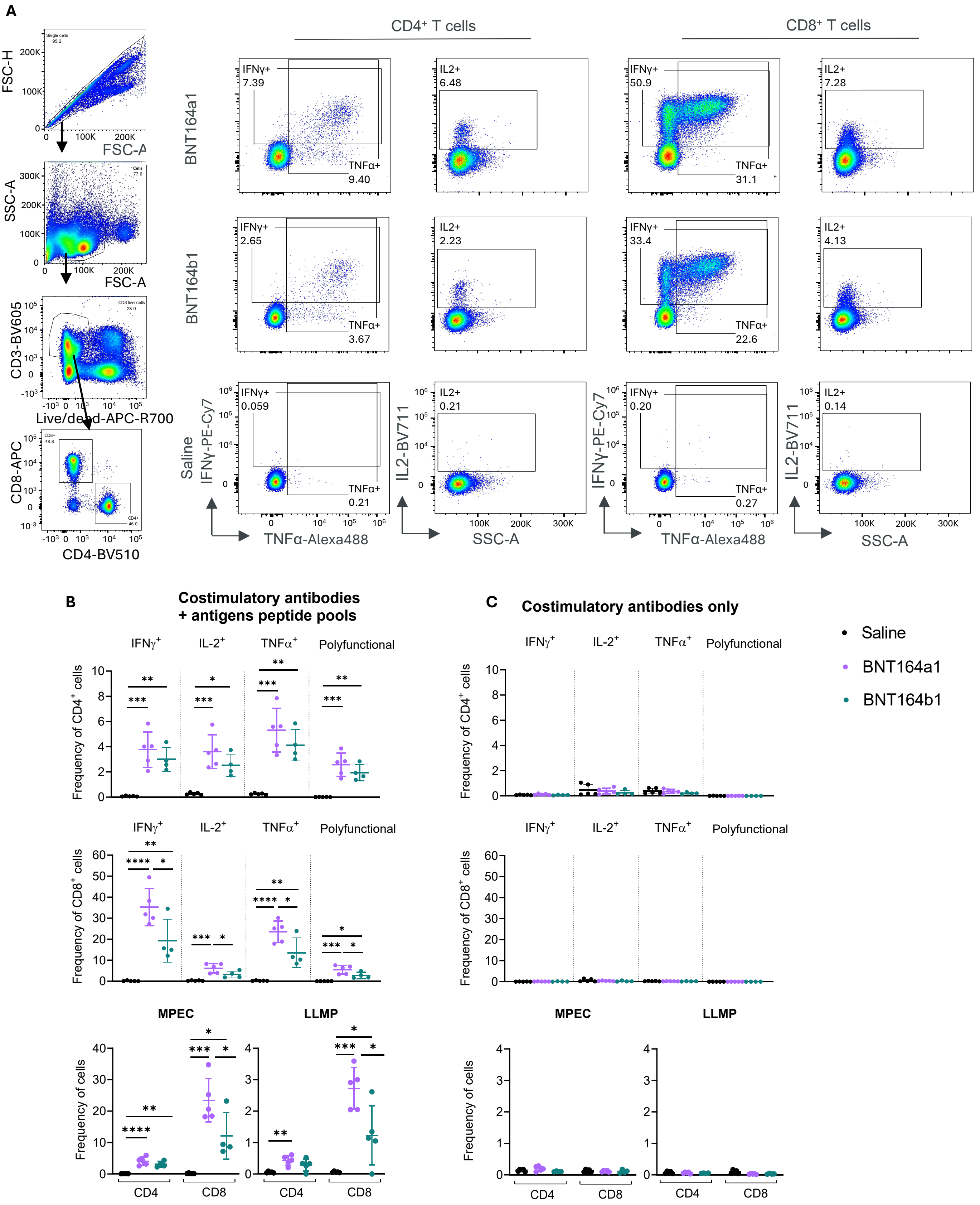

Supplementary Fig. 2. BNT164 candidates induced polyfunctional T cells and T cell memory

Splenocytes were isolated on Day 42 from C57BL/6 mice injected twice (Days 0 and 21) IM with 4 µg BNT164a1, 4 µg BNT164b1, or saline control. Splenocytes were stimulated with costimulatory antibodies (anti-CD49d and anti-CD28) with or without a mix of overlapping peptide pools covering all the encoded antigens. Cells were stained for intracellular and extracellular markers including viability, CD3, CD4, CD8, IFNγ, IL-2, TNFα, CD127, KLRG1, and CD62L. (A) The gating strategy and representative flow cytometry dot plots. (B–C) Percentage of CD4/CD8 cells positive for single cytokines and polyfunctional T cells ($IFN\gamma^+$/$IL\text{-}2^+$/$TNF\alpha^+$) (top and middle) and percentage of MPEC ($TNF\alpha^+$ or $IFN\gamma^+$/ $CD127^+$/$KLRG1^-$/$CD62L^-$) and LLMP ($TNF\alpha^+$ or $IFN\gamma^+$/ $CD127^+$/$KLRG1^-$/$CD62L^+$) CD4/CD8 cells (bottom). Group mean values are indicated by horizontal bars (± standard deviations); individual mouse values (n=5/group) are depicted as circles (one sample from BNT164b1 group was excluded due to insufficient cell number). One-way ANOVA with Tukey's multiple comparisons test was performed: * = $p < 0.05$; ** = $p < 0.01$; *** = $p < 0.001$; **** = $p < 0.0001$. IM = intramuscular; MPEC = memory precursor effector cell; LLMP = long-lived memory precursor cell.

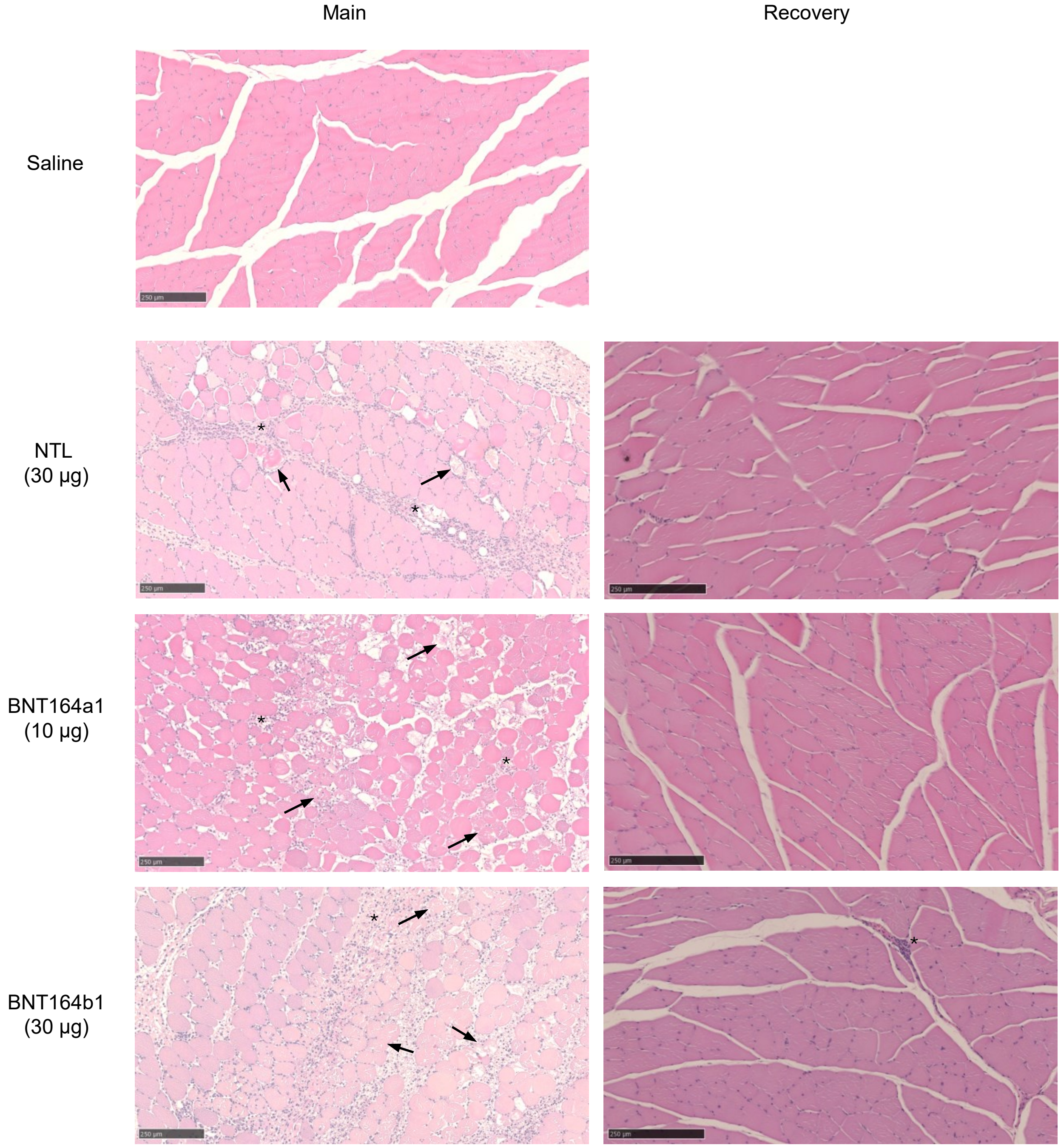

Supplementary Fig. 3. Histopathological assessment of injection sites in Wistar Han rats
Wistar Han rats were injected IM with saline, BNT164 non-translatable control (NTL, 30 µg), BNT164a1 (10 µg), or BNT164b1 (10 µg), on Days 0, 7, 14, and 21. Main study group was sacrificed on Day 23 (n=10), while the recovery group was sacrificed on Day 42 (n=5). Injected muscles were paraffin embedded, formalin fixed and stained with hematoxylin & eosin. Representative images are shown. Scale bars: 250 µm. *Indicates acute inflammation; arrows indicate examples of muscle fiber degeneration. IM = intramuscular.

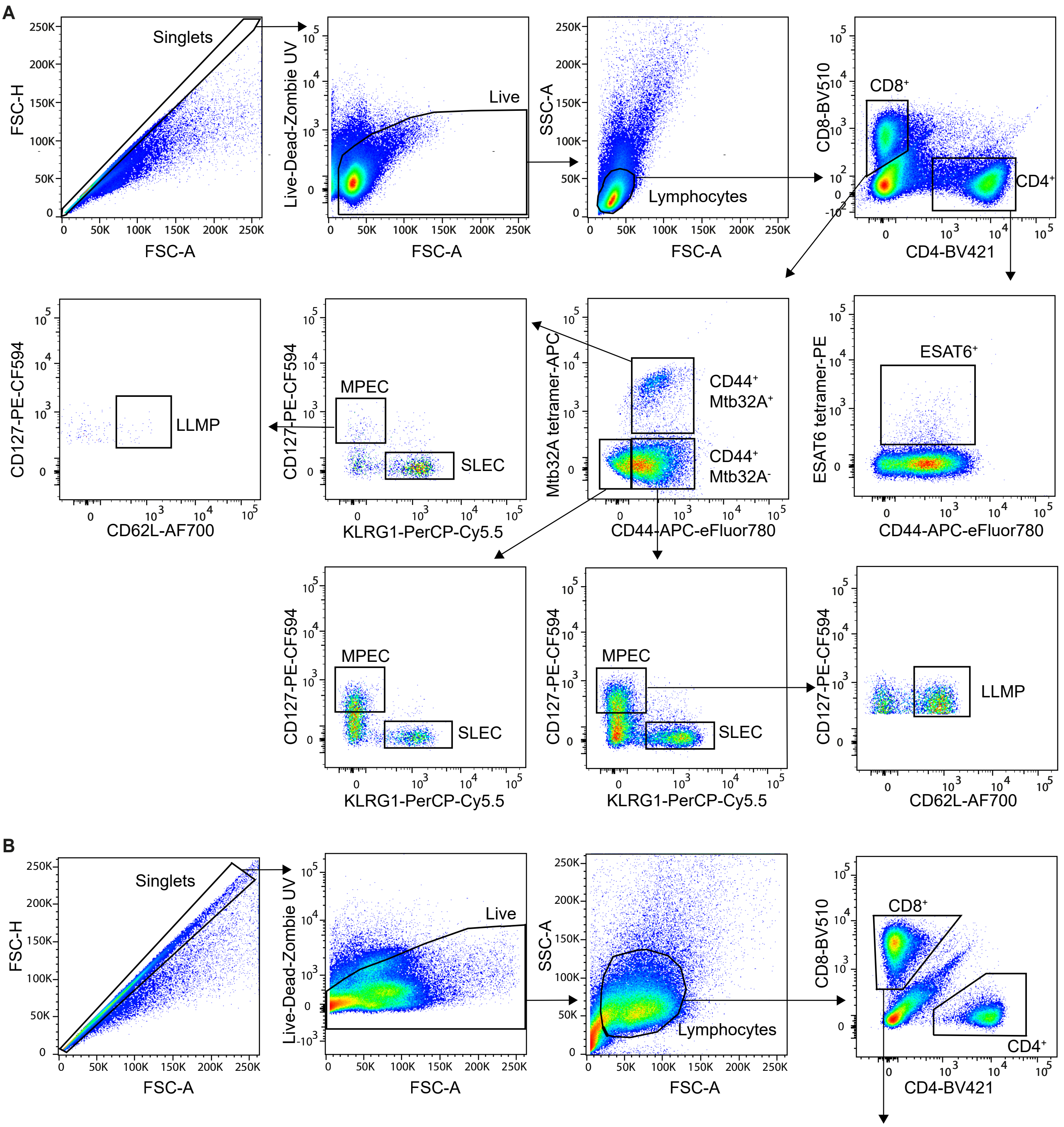

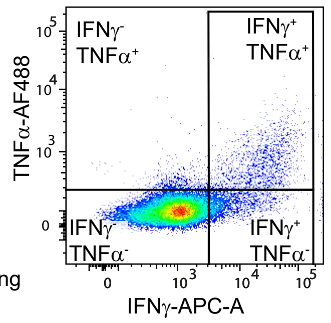

Supplementary Fig. 4. Gating strategy used for T-cell phenotyping in the Mtb HN878 challenge study
The study design is depicted in Fig 5A. Right lung lobes and mLNs were homogenized 30 days post-infection and analyzed by flow cytometry (A) directly or (B) after ex vivo stimulation of lung homogenate with overlapping peptide mixes of BNT164 antigens. Gating strategy is shown on lung cells of (A) a saline-injected mouse and (B) a BNT164a1-injected mouse.
