# Supplementary material for: mRNA-based tuberculosis vaccines BNT164a1 and BNT164b1 are immunogenic, well tolerated and efficacious in rodent models
Source: Nat Immunol. 2026 Jun 12;27(8):1653–65. doi: 10.1038/s41590-026-02545-z (PMC13414557; doi:10.1038/s41590-026-02545-z)

Source data: western blot images from Figure 1
Red rectangles indicate the parts that were cropped and shown in Figure 1.

Antigens bands

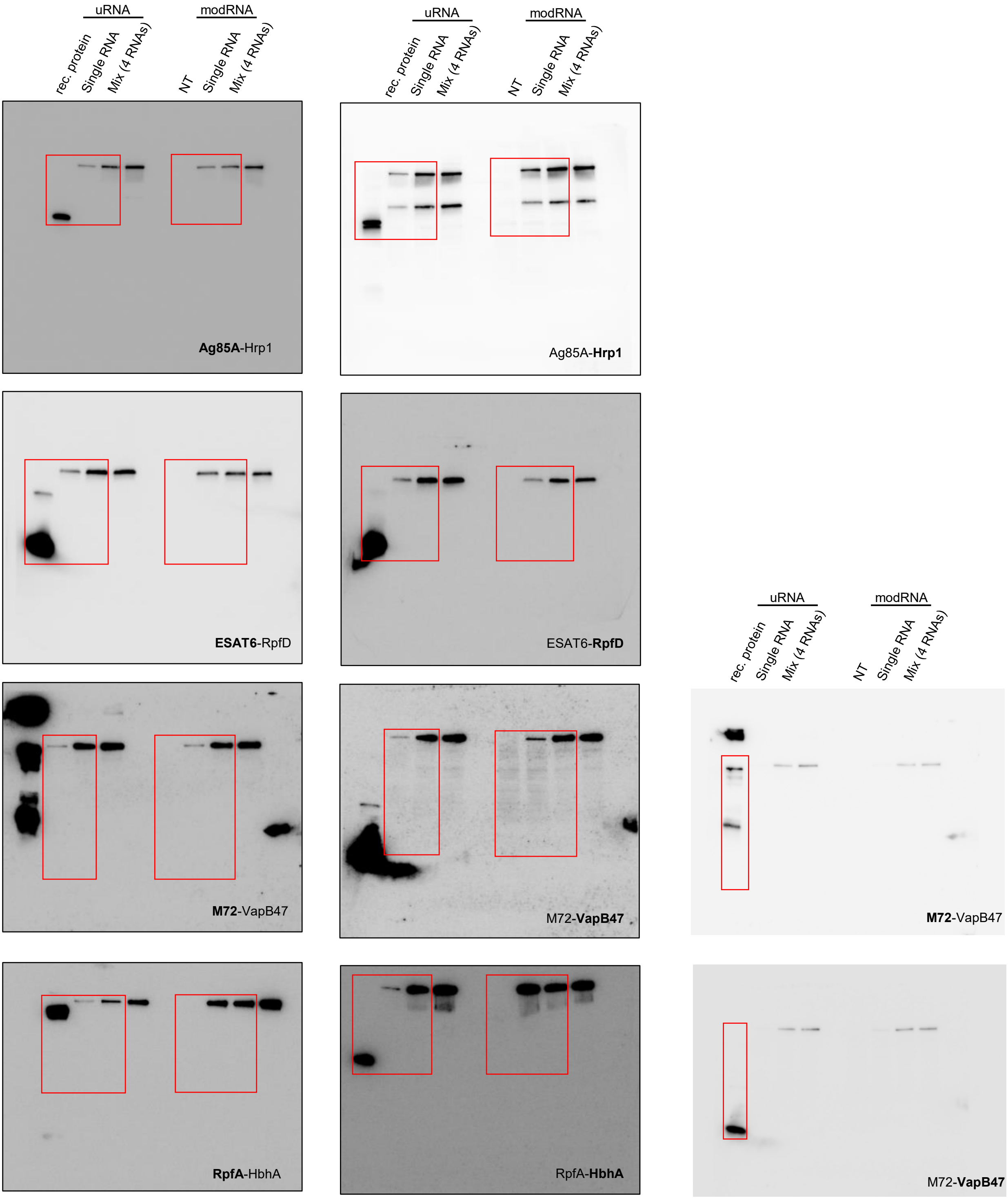

Tubulin bands

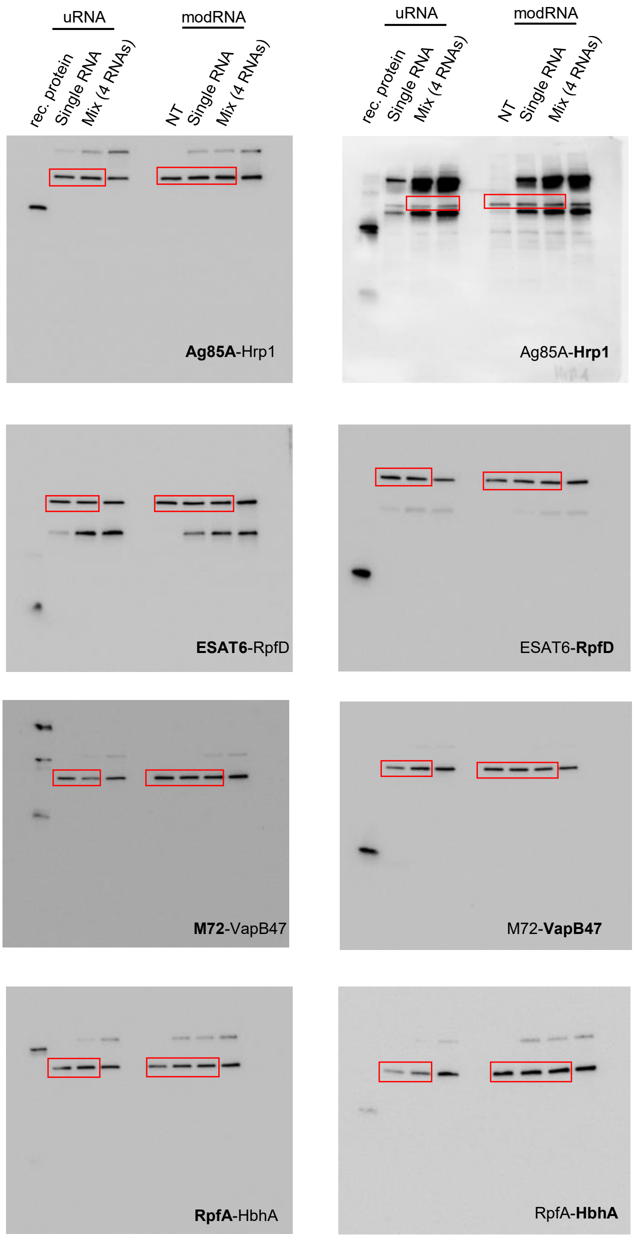

Supplement: Supplementary file 5 — Uncropped western blots used to generate Fig. 1. [file 41590_2026_2545_MOESM5_ESM.pdf]
