# Supplementary material for: mRNA-based tuberculosis vaccines BNT164a1 and BNT164b1 are immunogenic, well tolerated and efficacious in rodent models
Source: Nat Immunol. 2026 Jun 12;27(8):1653–65. doi: 10.1038/s41590-026-02545-z (PMC13414557; doi:10.1038/s41590-026-02545-z)
Supplement: Supplementary file 3 — Peer Review File [file 41590_2026_2545_MOESM3_ESM.pdf]

nature portfolio

Peer Review File

mRNA-based Tuberculosis Vaccines BNT164a1 and BNT164b1 are Immunogenic, Well Tolerated and Efficacious in Rodent Models

Corresponding Author: Dr Louis Ates

This file contains all editorial decision letters in order by version, followed by all author rebuttals in order by version.

Decision Letter:

18th Nov 2025

Dear Dr Ates,

Your Article, "mRNA-based Tuberculosis Vaccines BNT164a1 and BNT164b1 are Immunogenic, Well-tolerated and Efficacious in Rodent Models" has now been seen by 3 referees. You will see from their comments copied below that while they find your work of considerable potential interest, they have raised quite substantial concerns that must be addressed. In light of these comments, we cannot accept the manuscript for publication, but would be very interested in considering a revised version that addresses these serious concerns.

We hope you will find the referees' comments useful as you decide how to proceed. If you wish to submit a substantially revised manuscript, please bear in mind that we will be reluctant to approach the referees again in the absence of major revisions.

If you choose to revise your manuscript taking into account all reviewer and editor comments, please highlight all changes in the manuscript text file.

We are committed to providing a fair and constructive peer-review process. Do not hesitate to contact us if there are specific requests from the reviewers that you believe are technically impossible or unlikely to yield a meaningful outcome.

If revising your manuscript:

* Include a “Response to referees” document detailing, point-by-point, how you addressed each referee comment. If no action was taken to address a point, you must provide a compelling argument. This response will be sent back to the referees along with the revised manuscript.

* If you have not done so already please begin to revise your manuscript so that it conforms to our Article format instructions at http://www.nature.com/ni/authors/index.html. Refer also to any guidelines provided in this letter.

* Include a revised version of any required reporting checklist. It will be available to referees (and, potentially, statisticians) to aid in their evaluation if the manuscript goes back for peer review. A revised checklist is essential for re-review of the paper.

The Reporting Summary can be found here:
https://www.nature.com/documents/nr-reporting-summary.pdf

When submitting the revised version of your manuscript, please pay close attention to our href="https://www.nature.com/nature-portfolio/editorial-policies/image-integrity">Digital Image Integrity Guidelines. and to the following points below:

-- that unprocessed scans are clearly labelled and match the gels and western blots presented in figures.
-- that control panels for gels and western blots are appropriately described as loading on sample processing controls
-- all images in the paper are checked for duplication of panels and for splicing of gel lanes.

Extended Data figures and tables are online-only (appearing in the online PDF and full-text HTML version of the paper),

peer-reviewed display items that provide essential background to the Article but are not included in the printed version of the paper due to space constraints or being of interest only to a few specialists. A maximum of ten Extended Data display items (figures and tables) is typically permitted. When re-submitting your manuscript, please ensure that any supplementary figures and tables that are more critical to the manuscript's conclusions are converted to Extended data to increase these data's visibility.

Finally, please ensure that you retain unprocessed data and metadata files after publication, ideally archiving data in perpetuity, as these may be requested during the peer review and production process or after publication if any issues arise.

You may use the link below to submit your revised manuscript and related files:

Link Redacted

Note: This URL links to your confidential home page and associated information about manuscripts you may have submitted, or that you are reviewing for us. If you wish to forward this email to co-authors, please delete the link to your homepage.

If you wish to submit a suitably revised manuscript we would hope to receive it within 6 months. If you cannot send it within this time, please let us know. We will be happy to consider your revision so long as nothing similar has been accepted for publication at Nature Immunology or published elsewhere.

Nature Immunology is committed to improving transparency in authorship. As part of our efforts in this direction, we are now requesting that all authors identified as 'corresponding author' on published papers create and link their Open Researcher and Contributor Identifier (ORCID) with their account on the Manuscript Tracking System (MTS), prior to acceptance. ORCID helps the scientific community achieve unambiguous attribution of all scholarly contributions. You can create and link your ORCID from the home page of the MTS by clicking on 'Modify my Springer Nature account'. For more information please visit please visit www.springernature.com/orcid.

Please do not hesitate to contact me if you have any questions or would like to discuss the required revisions further.

Thank you for the opportunity to review your work.

Sincerely,
Paula

Paula Jauregui, PhD
Senior Editor
Nature Immunology

Version 0:

Reviewer comments:

Reviewer #1

(Remarks to the Author)
This manuscript presents the preclinical evaluation of two mRNA–LNP vaccines encoding eight Mycobacterium tuberculosis antigens. The work is timely in light of the need for improved TB vaccines and the growing interest in mRNA platforms. The dataset is extensive, and the fact that these constructs have advanced into clinical testing underscores the potential translational relevance. However, several aspects of the immunogenicity and efficacy data need to be addressed.

Major Comments
1. A central issue concerns the immunogenicity of the individual antigens. Although the vaccines encode eight full-length Mtb antigens, the data in Fig. 2 reveal that M72 is essentially the only antigen eliciting a strong T-cell response in either mouse strain. The remaining seven antigens—including Ag85A, ESAT-6, RpfA, RpfD, Hrp1, HbhA, and VapB47—induce only weak, inconsistent, or barely detectable T-cell responses, often hovering near background. This outcome undermines the rationale for a multi-antigen approach designed to span different stages of Mtb infection. If the T-cell response is effectively dominated by M72, it is unclear whether the other antigens contribute meaningfully to the vaccine's immunological profile or protective effect.

2. The humoral data raise similar concerns. In Fig. 3, several antigens—most notably ESAT-6, RpfD, HbhA, and VapB47—elicit little to no detectable IgG, even after boosting. These patterns reinforce the notion that a majority of the antigens in the construct do not generate measurable adaptive immune responses in this system. The authors should clarify whether this reflects poor antigen expression, suboptimal processing, intrinsic limitations of murine models, immunodominance by M72, or other factors. As currently presented, the data leave open the possibility that the protective responses observed are driven almost entirely by M72, raising the question of whether a simplified construct might perform comparably.

3. Another major limitation lies in the exclusive reliance on splenocytes to assess T-cell responses in Fig. 2B. TB is fundamentally a pulmonary disease, and protective immunity depends on T-cell populations that localize to the lung—such as lung-resident memory cells, Th1 and Th17 lineages, and $CD8^+$ memory precursor cells. Splenic responses often overestimate immunogenicity and may not reflect protective potential in the primary site of infection. Assessing antigen-specific responses in lung leukocytes or draining lymph nodes, or at least acknowledging this limitation explicitly, is essential for interpreting the data.

4. The efficacy data in Fig. 5 also require more careful contextualization. While the mRNA vaccines reduce bacterial burden by approximately half to one log in both H37Rv and HN878 challenge models, BCG performs equivalently—and in some cases better. Indeed, BCG consistently achieves roughly a one-log reduction, and in several panels the mRNA vaccines do not surpass this benchmark. It would be important for the authors to discuss these observations more explicitly and temper claims of efficacy accordingly. Given that the mRNA vaccines do not exceed BCG in these models, readers will reasonably question the mechanistic or translational rationale for advancing such constructs, especially if only one antigen induces strong adaptive immunity.

5. Beyond these specific issues, the manuscript would benefit from deeper mechanistic insight. For a study submitted to Nature Immunology, the current analysis is heavily phenotypic, relying on enumeration rather than mechanistic dissection. The authors touch on the potential differences between unmodified and modified mRNA platforms but do not explore early innate responses, antigen presentation dynamics, or tissue-resident memory formation, all of which are central to understanding TB vaccine efficacy. Likewise, the presence of $CD8^+$ memory precursor phenotypes in lung tissue is intriguing, but without functional data—such as depletion or blocking studies—it is difficult to ascertain their causal role in protection.

Reviewer #2

(Remarks to the Author)
In this manuscript, Agrawal et al evaluate safety profiles, immunogenicity, and efficacy of BNT164a1 and BNT164b1, two mRNA-LNP-based TB vaccine candidates, in preclinical rodent models. Each vaccine construct expresses 8 Mtb antigens that are expressed across different infection states. The two formulations (a1 and b1) represent differences in mRNA chemistry: nucleoside-unmodified RNA (uRNA, a1) and N1-methyl pseudouridine-containing modified RNA (modRNA, b1). The authors report that BNT164a1/b1 are immunogenic, well tolerated and efficacious in these preclinical studies in rodents. BNT164a1 and BNT164b1 represent promising TB vaccine candidates, and these preclinical studies represent an important step in providing rationale for continued development and testing of these candidates in clinical trials. A phase Ia trial the safety, reactogenicity, and immunogenicity of BNT164a1 and BNT164b1 was conducted non-BCG-vaccinated, Mtb-uninfected healthy adults in Germany (completed in 2025). A phase 1b/2a trial of the same candidates is currently ongoing in BCG-vaccinated, IGRA+ and IGRA- adults in Mozambique and South Africa (estimated completion in 2026). The manuscript is clearly presented and well written, with thorough reporting of the methods. However, there are limitations of the study that impact interpretation of the data as noted in the comments below.

Comments:
1. Figure 2 represents a schematic for immunogenicity studies. Were prior dose escalation experiments conducted that resulted in selection of 4ug dose of BNT164a1/b1? Do the authors have prior data as well to demonstrate that 2 doses of BNT164a1/b1 is more optimal than a single dose? If dosing strategy is based on prior studies of mRNA-based vaccines generated by BioNtech, this may be helpful to mention in the text.

2. Although vaccine-specific IgG responses were measured at 3 time points (d14, d28, d42), T cell immunogenicity is reported only at day 42. Data on the kinetics of T cell responses (peak immunogenicity time point) and durability and persistence of vaccine-induced T cell responses is lacking.

3. Figure 3: A third dose of BNT164a1/b1 was administered on day 134, which boosted IgG responses to RfpA, M72, HbhA, and VapB47. It is unclear why vaccine-induced T cell responses were not also evaluated at later time points and/or after administration of a 3rd vaccine dose.

4. Lines 201-203: muscle fiber damage and inflammatory reaction related to BNT164 administration are noted as “data not shown”. It would be useful to include these data in Supplementary Figure 5.

5. BNT164a1/b1-induced T cell immunogenicity studies were conducted in splenocytes only. For TB vaccine candidates, it would also be important to demonstrate the presence of Ag-specific T cells in the lung (there are limited data from tetramer staining conducted in the lung post-Mtb challenge, but otherwise no immunogenicity studies of T cells in the lung after BNT164a1/b1 immunization).

6. No ESAT6- and RpfD-specific IgG responses were induced in any of the 3 mouse strains by either vaccine construct. Can the authors comment on why these antigens are not inducing IgG antibodies? Did the investigators evaluate induction of other Ab isotypes?

7. Figure 5B, C: were statistical comparisons performed for CFUs between BCG, BNT164a1, and BNT164b1-immunized mice post-challenge? CFUs for BNT164a1/b1-immunized mice appear to be higher in the lung and spleen 30 days post-challenge, compared with BCG-immunized mice. The results section describes comparison of CFU between BNT164a1/b1-immunized and saline controls only, but it would be important to demonstrate protection of BNT164a1/b1 relative to BCG, the only currently licensed TB vaccine.

8. There does not seem to be a group of animals that is primed with BCG, then immunized with BNT164a1/b1, which would more closely represent real-world applications of this vaccine in which much of the population at risk for TB reside in countries in which BCG is routinely administered. Since BNT164a1/b1 immunization alone does not seem to be more protective than BCG (by CFU data in Fig 5), it would be interesting to see if BCG prime + boosting with BNT164a1/b1 is more protective than either BCG prime alone or BNT164a1/b1 prime/boost alone.

9. Evaluation of Ag-specific T cell responses in the lungs and mLNs of mice post-challenge is restricted to evaluation of frequency of CD8 T cells staining with one M72-specific tetramer and CD4 T cells staining with one ESAT-6 tetramer. Given the 8 antigens encoded by BNT164a1/b1, it remains unclear what the overall frequency of Ag-specific CD4 and CD8 T cells is in the lungs and lymph nodes of BNT164a1/b1-immunized mice. It is not clear why the investigators did not also conduct intracellular cytokine staining assays to get a more complete measurement of Ag-specific T cells in the lungs/LN.

10. Figure 6 and Supplementary Figure 7: tetramer staining of Ag-specific T cells in the lung is depicted as % of CD8 T cells. The data should also be presented as absolute number of tetramer+ cells in the lung.

11. Supplementary Figure 7: ESAT6 tetramer+ CD4 T cells are low frequency and not a clearly defined population. It would be useful to show ESAT6 tetramer staining of lung CD4 T cells from an uninfected mouse to better evaluate background/non-specific binding of this tetramer.

12. Conclusions regarding elicitation of lung-resident CD8 T cells by BNT164a1/b1 and interpretation of these cells as potential correlates of protection should be interpreted with caution, given the limited approaches used to evaluate Ag-specific T cells (which are restricted to 1 CD8 tetramer and 1 CD4 tetramer that did not demonstrate evidence of clear staining). Measurement CD4 and CD8 T cells in the lung that are specific for the 8 antigens in BNT164a1/b1 could have been more comprehensively evaluated by intracellular cytokine staining.

13. Lines 278-287: Supplementary Figure 7 is repeatedly referred to as Fig S77.

Reviewer #3

(Remarks to the Author)
Review for "A Multi-Antigen mRNA-LNP Vaccine Elicits T-Cell Immunity and Protection Against Mycobacterium tuberculosis in Preclinical Models" by Agrawal et. Tuberculosis remains the leading infectious disease cause of death worldwide. The currently available approved vaccine against TB provides protection against TB in newborns, but limited to protection to adolescents or adults, that harbor the majority of disease and drive transmission. The current manuscript addresses this gap, and describes a tour de force study focused on the development of an Mtb mRNA/LNP vaccine consisting of key Mtb antigens able to induce protection against Mtb in rodents.

The COVID-19 pandemic highlighted the potency and flexibility of the mRNA/LNP vaccine platform, able to induce robust Th1 immunity. Given the flexibility and multivalent capacity of mRNA, the authors selected an 8 antigen cocktail, representing key antigens that have been shown to provide protection historically using a CMV-vectored vaccine, as well as including 2 additional antigens, one that was recently shown to provide protection in a human Phase2b trial (M72) and one that was shown to provide protection against dissemination (HBHA). The authors designed 4 fusion proteins, encoded on 4 mRNAs, using either modified (BNT164b1) or unmodified (BTN164a1) mRNA, that produced antigens in vitro, all showed comparable T and B cell immunogenicity in rodent models, safety in a tox rat model, and afforded protection against challenge with 2 Mtb strains, in the setting of infiltrating T cells into lungs.

The major advances include:
-The study includes a multi-antigen design, covering antigens expressed at key steps of bacterial infection selected based on their previous capacity to provide protection against Mtb, all of which were screened rigorously and expressed in vitro and showed some level of both B and T cell immunogenicity in vivo. These are absolutely critical data as the expression of bacterial antigens in a mammalian cell in a manner that can induce conformationally correct immunity (based on antibody binding to Mtb lysates and recombinant antigens) is a major step forward for the mRNA vaccine world.
-The study highlights the induction of robust CD8 T cell responses, that have not been induced readily by other vaccine platforms that are currently in testing across the globe, but that have been linked to the control and elimination of Mtb, immunity that is currently not elicited by other Mtb vaccines that are in preclinical or clinical development
-Given the emerging appreciation for the potential role for antibodies in Mtb control, the study also demonstrates the induction of humoral immune responses to both individual antigens but also to Mtb lysates, including durability and boostability data for nearly all tested antigens, that is likely to be absolutely essential for long-lived protection for decades of life in highly endemic areas.
-The study explores protection not just against the commonly used lab strain of Mtb (H37Rv), but also a more virulent Mtb strain (HN878).
- The study provides interesting first insights on correlates of immunity in the lungs by tissue immune-histochemistry and flow

cytometry demonstrating the infiltration of CD8+ T cells in the lungs of mRNA immunized animals.

This incredible body of work provides robust, mechanistic evidence to support the development of this vaccine in humans, marking the development of an exciting new strategy to combat Mtb via the potential induction of lung-trafficking T cells, long-lived humoral immunity, and robust boostability, that may be key to protection against Mtb in adolescents, most vulnerable to the disease. With human testing already underway, these data will be of great interest to the TB field, moving the entire field closer to a transmissible-disease-blocking vaccine. However, there are a few comments below that could improve the manuscript:

1. The authors describe the rationale for the selection of the 8 antigens and provide a cartoon of the designs. However, more detail on the precise arrangement and linkers used to generate these fusions, and the arrangement of each of the genes could be very helpful and provide completeness for the paper. Alpha-fold projections of the fusion constructs and single proteins can help readers visualize the likely preservation of antigenicity. Some rationale for why particular sets was fused could also be helpful.
2. Interference across antigens has been observed with multiplexing. While the authors show the expression of individual proteins in vitro and immunogenicity in vivo, it would be helpful for the authors to include data on single immunization efforts to understand the level – if any – of interference that was observed during the design stage.
3. Given the paucity of reagents to test for correct conformation/antigenicity, the authors used a tag to measure protein expression. A monoclonal antibody was used to test the antigenicity of Ag85a. Given that Mtb exposed individuals are also likely exposed to these antigens, it would be quite interesting to see whether mRNA encoded antigens can be recognized by plasma samples from individuals naturally exposed to Mtb as a translational datapoint.
4. The authors show T and B cell immunity as individual datapoints from the same mouse studies, however whether B and T cell responses are induced in a coordinated manner is unclear. It would be helpful if the authors could include some correlational analyses to help the reader understand if antigens induced more coordinated T/B cell immune responses- or whether some antigens were strong B or T cell targets.
5. Substantial variation was observed in T cell immune induction across the antigens. It is unclear if this is related to differences in antigen expression. Was there any correlation between in vitro expression levels and likelihood of seeing a T cell immune response? Additionally, despite the variation across mouse strains, it is unclear whether these responses are correlated (ie. If the response is high in a B6 is it also high in an A2 or BALBc)? This knowledge would be incredibly helpful for the field and readers.
6. There is limited discussion on why limited differences were not observed across modified and unmodified mRNA that showed dramatic differences for COVID-19 vaccines in both humans and NHP. Some discussion should focus on this biology to help readers contextualize these results in the broader understanding of the use of these different mRNA types.
7. Moreover, despite the limited expression and peak immunogenicity levels across the 2 mRNA types, BNT164b1 exhibited a 40-fold boost compared to BNT164a1 (8-fold) . Do the authors believe this difference is related to the better priming by modified mRNA?
8. For infiltrates, the authors demonstrate the selective increase in CD8+ T cell populations, and mention limited CD4+ T cell infiltration. The inclusion of negative data can still be helpful to the field. Additionally, were other lymphocytes, including NK cells also observed, potentially important given the presence of vaccine induced antibodies that may have functional impact on limiting bacterial growth.
9. Finally, for the protection studies, it is unclear whether M72, currently in Phase3 trials, was tested alone, but could provide vital insights into the additional benefit of more antigens that may help provide enhanced protection against the bacteria.

Decision Letter:

13th Mar 2026

Dear Dr Ates,

Your Article, "mRNA-based Tuberculosis Vaccines BNT164a1 and BNT164b1 are Immunogenic, Well-tolerated and Efficacious in Rodent Models" has now been seen by 3 referees. You will see from their comments below that while they find your work of interest, some important points are raised. We are very interested in the possibility of publishing your study in Nature Immunology, but would like to consider your response to these concerns in the form of a revised manuscript before we make a final decision on publication.

Please address the remaining issues from reviewer #3 that can be solved with text changes, adding a statement that BCG is not protective in humans despite it being a benchmark in mice, that M72 was protective in humans but not in mice, and a note in the discussion related to the use of mRNA vaccines to prevent respiratory viruses using intra-muscular immunization. Reviewer #3 also wants you to add a heat map of all the integrated data. Please also convert some of the Supplementary Figures into Extended Data Figures. We only allow up to 10 Extended Data Items, so we suggest to covert to Extended Data Figures all the data that would be more important to be more accessible to readers.

We therefore invite you to revise your manuscript taking into account all reviewer and editor comments. Please highlight all changes in the manuscript text file in Microsoft Word format.

We are committed to providing a fair and constructive peer-review process. Do not hesitate to contact us if there are specific requests from the reviewers that you believe are technically impossible or unlikely to yield a meaningful outcome.

When revising your manuscript:

* Include a “Response to referees” document detailing, point-by-point, how you addressed each referee comment. If no action was taken to address a point, you must provide a compelling argument. This response will be sent back to the referees along with the revised manuscript.

* If you have not done so already please begin to revise your manuscript so that it conforms to our Article format instructions at http://www.nature.com/ni/authors/index.html. Refer also to any guidelines provided in this letter.

* Please include a revised version of any required reporting checklist. It will be available to referees to aid in their evaluation of the manuscript goes back for peer review. They are available here:

Reporting summary:
https://www.nature.com/documents/nr-reporting-summary.pdf

When submitting the revised version of your manuscript, please pay close attention to our href="https://www.nature.com/nature-portfolio/editorial-policies/image-integrity">Digital Image Integrity Guidelines. and to the following points below:

-- that unprocessed scans are clearly labelled and match the gels and western blots presented in figures.
-- that control panels for gels and western blots are appropriately described as loading on sample processing controls
-- all images in the paper are checked for duplication of panels and for splicing of gel lanes.

Please note, Extended Data figures and tables are online-only (appearing in the online PDF and full-text HTML version of the paper), peer-reviewed display items that provide essential background to the Article but are not included in the printed version of the paper due to space constraints or being of interest only to a few specialists. A maximum of ten Extended Data display items (figures and tables) is typically permitted. When re-submitting your manuscript, please ensure that any supplementary figures and tables that are more critical to the manuscript’s conclusions are converted to Extended data to increase these data’s visibility.

Finally, please ensure that you retain unprocessed data and metadata files after publication, ideally archiving data in perpetuity, as these may be requested during the peer review and production process or after publication if any issues arise.

Please use the link below to submit your revised manuscript and related files:

Link Redacted

Note: This URL links to your confidential home page and associated information about manuscripts you may have submitted, or that you are reviewing for us. If you wish to forward this email to co-authors, please delete the link to your homepage.

We hope to receive your revised manuscript within two weeks. If you cannot send it within this time, please let us know. We will be happy to consider your revision so long as nothing similar has been accepted for publication at Nature Immunology or published elsewhere.

Please do not hesitate to contact me if you have any questions or would like to discuss these revisions further.

Nature Immunology is committed to improving transparency in authorship. As part of our efforts in this direction, we are now requesting that all authors identified as ‘corresponding author’ on published papers create and link their Open Researcher and Contributor Identifier (ORCID) with their account on the Manuscript Tracking System (MTS), prior to acceptance. ORCID helps the scientific community achieve unambiguous attribution of all scholarly contributions. You can create and link your ORCID from the home page of the MTS by clicking on ‘Modify my Springer Nature account’. For more information please visit please visit www.springernature.com/orcid.

We look forward to seeing the revised manuscript and thank you for the opportunity to review your work.

Sincerely,
Paula

Paula Jauregui, PhD
Senior Editor
Nature Immunology

Version 1:

Reviewer comments:

Reviewer #1

(Remarks to the Author)
The authors have satisfactorily addressed my concerns, and the paper is now substantially improved.

Reviewer #2

(Remarks to the Author)
The authors have provided thoughtful and thorough responses to each of the concerns raised on the initial submission. I believe the revisions have strengthened the manuscript. The data presented in the manuscript are overall descriptive and less depth into mechanisms of protection mediated by this vaccine platform. Nevertheless, these mRNA TB vaccine constructs are important data for advancing the TB vaccine field the study is overall well done. I would have like to see more functional analysis of vaccine Ag-specific T cell responses at more time points, although I understand the limitations noted by the authors in response to this comment. I don't have any additional concerns for the authors to address.

Reviewer #3

(Remarks to the Author)
The authors responded skillfully across a diverse set of questions from all 3 reviewers.

While the immunological data is very clear, it is still not clear to me precisely which mice mounted antibodies and/or T cells. A heat map of all the integrated data could be very helpful even if only as supplemental data.

The supplemental figure with the alpha-fold proteins is an important addition. I would suggest pulling this into figure 1 for transparency on the predicted constructs.

It will be important to add a statement about the failures of the BCG revaccination studies that showed BCG is not protective in humans despite it being a benchmark in mice - and further emphasizing that M72 was protective in humans but not in mice. This will help contextualize these data that are essential to derisk the transition to humans which will be most informative. It may also be important to move the BCG results in the challenge study to the right side of the graph - as it is a distracting comparison - but a necessary control.

It may be important to add a note in the discussion related to the use of mRNA vaccines to prevent COVID, Flu, and RSV - all respiratory viruses using intra-muscular immunization. Although the vaccines do not prime T cells within the lungs, the platform has proven to be quite effective against respiratory pathogens, demonstrating an ability to raise peripheral T cells that can effectively migrate and respond to infections in the lungs.

Version 2:

Decision Letter:

Our ref: NI-A41473B

31st Mar 2026

Dear Dr. Ates,

Thank you for submitting your revised manuscript "mRNA-based Tuberculosis Vaccines BNT164a1 and BNT164b1 are Immunogenic, Well-tolerated and Efficacious in Rodent Models" (NI-A41473B). It has now been seen by the original referees and their comments are below. The reviewers find that the paper has improved in revision, and therefore we'll be happy in principle to publish it in Nature Immunology, pending minor revisions to comply with our editorial and formatting guidelines.

We will now perform detailed checks on your paper and will send you a checklist detailing our editorial and formatting requirements in about a week. Please do not upload the final materials and make any revisions until you receive this additional information from us.

If you had not uploaded a Word file for the current version of the manuscript, we will need one before beginning the editing process; please email that to immunology@us.nature.com at your earliest convenience.

Thank you again for your interest in Nature Immunology Please do not hesitate to contact me if you have any questions.

Sincerely,
Paula

Paula Jauregui, PhD
Senior Editor
Nature Immunology

Open Access This Peer Review File is licensed under a Creative Commons Attribution 4.0 International License, which permits use, sharing, adaptation, distribution and reproduction in any medium or format, as long as you give appropriate credit to the original author(s) and the source, provide a link to the Creative Commons license, and indicate if changes were made.
In cases where reviewers are anonymous, credit should be given to 'Anonymous Referee' and the source.
The images or other third party material in this Peer Review File are included in the article's Creative Commons license, unless indicated otherwise in a credit line to the material. If material is not included in the article's Creative Commons license and your intended use is not permitted by statutory regulation or exceeds the permitted use, you will need to obtain permission directly from the copyright holder.
To view a copy of this license, visit https://creativecommons.org/licenses/by/4.0/

Response to reviewers.

We thank the reviewers for their fair and thorough feedback.

The reviewer comments are cited in blue italic font. Our responses are provided in line in black.

The uploaded revised manuscript file highlights all changes in yellow.

1. Reviewer #1 (Remarks to the Author):

This manuscript presents the preclinical evaluation of two mRNA–LNP vaccines encoding eight Mycobacterium tuberculosis antigens. The work is timely in light of the need for improved TB vaccines and the growing interest in mRNA platforms. The dataset is extensive, and the fact that these constructs have advanced into clinical testing underscores the potential translational relevance. However, several aspects of the immunogenicity and efficacy data need to be addressed.

Major Comments

1. A central issue concerns the immunogenicity of the individual antigens. Although the vaccines encode eight full-length Mtb antigens, the data in Fig. 2 reveal that M72 is essentially the only antigen eliciting a strong T-cell response in either mouse strain. The remaining seven antigens—including Ag85A, ESAT-6, RpfA, RpfD, Hrp1, HbhA, and VapB47—induce only weak, inconsistent, or barely detectable T-cell responses, often hovering near background. This outcome undermines the rationale for a multi-antigen approach designed to span different stages of Mtb infection. If the T-cell response is effectively dominated by M72, it is unclear whether the other antigens contribute meaningfully to the vaccine's immunological profile or protective effect.

We thank the reviewer for the encouraging general words on our manuscript, underscoring the timeliness and translational potential of our work. We understand the concern of the reviewer in observing limited immunogenicity of certain antigens in specific models. When the data are analyzed holistically across the three different mouse models (Fig. 2 C-D, Fig. S2 and Fig. S3), it becomes clear that the strength of T-cell responses against specific antigens is not consistent across models. These aggregated data also do not suggest M72 immune dominance, as most other antigens induced robust T-cell responses in two out of three tested mouse models. Multiple antigens that did not induce T-cell responses in one model reached the upper limit of detection in another model (e.g., Hrp1, HbhA, VapB47, RpfA). In addition, the newly added Fig. S7 (in response to reviewer 2) shows that the third BNT164 dose further

boosts T-cell responses to multiple antigens in the C57BL/6 model, including Ag85A, RpfA and ESAT-6.

Furthermore, we have included a new dataset addressing possible antigen interference as Fig. S6. We compared the splenocyte and antibody responses between the BNT164 candidate (4x2 antigens) and the four individual RNA-cassettes and observed minor, yet statistically significant, differences in T-cell immunogenicity when combining the antigen cassettes. However, the small effect sizes and trends going in both directions illustrate that there is no immunodominant effect of M72 overriding T-cell responses to other antigens. Finally, we added intracellular cytokine staining of lung CD8+ cells after *Mtb* challenge as Fig. S12 G-H. Cell stimulation was done with two different peptide pools of comparable size, with one peptide pool covering M72, VapB47 and Ag85A, and the second peptide pool covering the other five antigens. IFNγ and TNFα production were seen in response to both pools and were slightly more pronounced in response to the peptide pool without M72. Taken together, our data show that CD8+ T-cell responses are not limited to M72-specific T-cells.

We believe that observed differences in T-cell responses across mouse models are rather due to MHC/HLA differences. We based our antigen selection predominantly on immunogenicity and protection data from humans and non-human primates (NHP). Because of high HLA diversity in the human population, we did not exclude antigens based on limited immunogenicity in any single specific mouse model. Rather, we used this combined multi-model immunogenicity framework to show that all antigen cassettes are delivered efficiently and induce immunogenicity if the model genetic background allows this. Importantly, out of three tested mouse models, the most robust T-cell responses were induced in the human HLA-A02:01/DR1 transgenic mice (Fig. S3 C), confirming that BNT164 antigens are compatible with these human HLA alleles.

We realize that this approach was insufficiently introduced in the manuscript and that an integrated overview across models was missing.

Therefore, we have:

1) Added a new figure with a visual overview of splenocyte responses across models (Fig. 2E) and added the following text to the results section:
   Taken together, all BNT164-encoded antigens raised T-cell responses in at least two out of three tested mouse models, with observed differences likely being driven by MHC/HLA variations between those mouse models.
2) Added a section to the discussion, addressing the differences in T-cell responses across models:
   Antigen selection for BNT164 candidates was driven by high quality immunogenicity data in humans and, where available, efficacy signals in humans or NHP (Table S1). To reduce the murine bias in BNT164 immunogenicity testing,

we used three different mouse models with varying MHC/HLA backgrounds. Antigens with limited T-cell immunogenicity in one model, such as VapB47 in C57BL/6 or ESAT-6 in BALBc, performed well in other models. Furthermore, antigens with low T-cell immunogenicity in C57BL/6, such as Hrp1 and VapB47, induced IgG responses, confirming they were successfully expressed. Taken together, these results indicate that delivery and expression of BNT164 antigens is consistent in tested murine models and that differences in splenocyte immunogenicity are likely driven by MHC/HLA differences. The data, therefore, support the inclusion of all eight antigens into the final BNT164 candidates aimed at diverse human HLA backgrounds.

3) Added a new dataset addressing the antigen interference (Fig. S6) and corresponding description, as specified in the reply to the reviewer's question 2 below.
4) Added intracellular cytokine staining of lung CD8+ cells with two different peptide pools (Fig. S12 G-H). Further details are provided in response to question 9 by reviewer 2.

2. The humoral data raise similar concerns. In Fig. 3, several antigens—most notably ESAT-6, RpfD, HbhA, and VapB47—elicit little to no detectable IgG, even after boosting. These patterns reinforce the notion that a majority of the antigens in the construct do not generate measurable adaptive immune responses in this system. The authors should clarify whether this reflects poor antigen expression, suboptimal processing, intrinsic limitations of murine models, immunodominance by M72, or other factors. As currently presented, the data leave open the possibility that the protective responses observed are driven almost entirely by M72, raising the question of whether a simplified construct might perform comparably.

We thank the reviewer for their comment, which encompasses multiple related yet distinct concerns. We agree that little to no humoral responses are induced to ESAT-6 and RpfD and discuss this in detail below. However, we do not agree with the reviewer's statement that HbhA and VapB47 "elicit little to no detectable IgG, even after boosting". A two-dose regimen clearly induced specific titers to these antigens in C57BL6 and BALB/c mice (Fig. 3A, Fig. S5A). Furthermore, after the third dose ΔOD values are above 1.0 even at the relatively high serum dilution of 1:8,100. The assertion that the protective responses observed are driven almost entirely by M72 is not supported by presented data (Fig. 3 and Fig. S5A), as IgGs against Ag85A, Hrp1 and RpfA, are consistently detected at similar or higher levels compared with those against M72.

Regarding the lack of humoral responses to ESAT-6 and RpfD, this was noted by all three reviewers and we appreciate the request to discuss this matter in more detail. First, the lack of IgG titers to ESAT-6 and RpfD cannot be explained by poor antigen

expression, since the cassette induces robust T-cell responses, especially in the C57BL/6 and humanized HLA models. Newly added antigen interference data (Fig. S6B) shows that the loss of ESAT-6 antibodies was a clear example of antigen interference in the octavalent BNT164b1 candidate, compared to the individual RNA cassette. As for RpfD, antibodies were not raised with either BNT164b1 or individual RNA cassette. We suspected the lack of antibodies to RpfD to be a consequence of the predicted transmembrane domain in this protein. Efforts to optimize antibody responses to the antigen cassette through modification or deletion of this transmembrane domain did not markedly improve antibody responses. Rather, the modifications strongly impaired the induced T-cell responses to RpfD, as this domain contains highly immunogenic T-cell epitopes. Since ESAT-6 and RpfD were included as T-cell targets (Hansen et al. 2018, Nat. Med.), the lack of antibody responses was considered acceptable in the context of an octavalent vaccine candidate. The manuscript was adapted to include additional data (Fig. S6) and discussion on lack of IgG antibodies to ESAT-6 and RpfD.

Results (Section "BNT164 candidates elicit cellular and humoral immunogenicity in mice"):

Antigen interference is important to investigate for multivalent vaccine candidates. After confirming no interference at expression level (Fig. 1), we also investigated interference at immunogenicity level in vivo. To this end, we compared BNT164b1 (4 µg) and four individual antigenic cassettes of BNT164b1 (1 µg) in prime-boost regimen in C57BL/6 mice. For T-cell responses, a modest antigen interference was observed for Hrp1, RpfA and HbhA, as mice immunized with individual cassettes showed higher responses than BNT164b1-immunized mice (Fig. S6A). IgG responses were overall comparable between groups, except for ESAT-6 (Fig. S6B). Whereas immunization with the individual cassette induced an IgG response to ESAT-6, this response was abrogated upon combining with the other three cassettes in BNT164b1. Since ESAT-6 was primarily included in BNT164 as a T-cell antigen, loss of ESAT-6 antibody responses was considered acceptable. Taken together, interference between eight selected BNT164 antigens was acceptable for an octavalent vaccine, with most antigen-specific immune responses being preserved.

Discussion:

No IgG responses were detected against RpfD in our experiments, and antibodies against ESAT-6 were abrogated in the octavalent BNT164b1 candidate compared with individual ESAT-6-RpfD mRNA cassette, suggesting antigen interference. This was considered acceptable, since ESAT-6 and RpfD were primarily included as T-cell antigens.

3. Another major limitation lies in the exclusive reliance on splenocytes to assess T-cell responses in Fig. 2B. TB is fundamentally a pulmonary disease, and protective

immunity depends on T-cell populations that localize to the lung—such as lung-resident memory cells, Th1 and Th17 lineages, and $CD8^+$ memory precursor cells. Splenic responses often overestimate immunogenicity and may not reflect protective potential in the primary site of infection. Assessing antigen-specific responses in lung leukocytes or draining lymph nodes, or at least acknowledging this limitation explicitly, is essential for interpreting the data.

We acknowledge this is an important question and we believe our data already partially provide some answers. We show increased CD8+ T-cell infiltration in the lungs of BNT164-immunized mice upon *Mtb* challenge (Fig. 6D), suggesting successful migration of raised T cells from the spleen or periphery to the infection site. Furthermore, we show an increase in memory precursor effector CD8+ cells and long-lived memory precursor CD8+ cells in the lung (Fig. 6 F and I), and Mtb32A-tetramer+ (antigen-specific) CD8+ cells in the lung and draining lymph node (Fig. 6 E and H). We have also added intracellular cytokine staining of lung CD8+ T-cells upon peptide stimulation with two different peptide pools together covering all eight BNT164 antigens (Fig. S12 G-H). These data provide supporting evidence that detected pulmonary CD8+ cells are antigen-specific and functional, although we acknowledge limited data granularity. Regarding other T-cell subsets and further organ-specific responses, we have now included this limitation in the discussion.

The updated text in the Discussion reads:

Observed lymphocyte infiltration in the lung was driven by antigen-specific $CD8^+$ T cells, indicating that systemically induced T-cells have the capacity to migrate to the site of infection. Intracellular cytokine staining, after stimulation with antigens' peptide pools, confirmed these lung $CD8^+$ cells are functional cytokine-secreting cells. Furthermore, the BNT164-induced $CD8^+$ T cells exhibited memory precursor phenotypes both in the spleen in the immunogenicity study, and in the lung after Mtb challenge. More research is required to fully characterize lung resident memory formation following BNT164 immunization.

4. The efficacy data in Fig. 5 also require more careful contextualization. While the mRNA vaccines reduce bacterial burden by approximately half to one log in both H37Rv and HN878 challenge models, BCG performs equivalently—and in some cases better. Indeed, BCG consistently achieves roughly a one-log reduction, and in several panels the mRNA vaccines do not surpass this benchmark. It would be important for the authors to discuss these observations more explicitly and temper claims of efficacy accordingly. Given that the mRNA vaccines do not exceed BCG in these models, readers will reasonably question the mechanistic or translational rationale for advancing such constructs, especially if only one antigen induces strong adaptive immunity.

We thank the reviewer for requesting more explanation on this important matter. It is known that BCG is highly protective in these murine models, but offers little to no protection against disease in the adult human setting (Schmidt *et al*. 2025, NEJM). Therefore, a direct comparison with BCG protection is not valid in these mouse models. We acknowledge this limitation was not sufficiently discussed and have now addressed these points more explicitly in the discussion. A reference was added to the excellent recently published work, systematically comparing published clinical vaccine candidate responses in animal models (Pozo-Ramos & Kupz 2025, Front. Immunol.).

The following section on the interpretation of murine efficacy was added to the discussion:

It is also important to note that BCG was included in our studies as a positive control but should not be used for efficacy comparisons, as BNT164 candidates are aimed at different indications and target populations. However, BNT164 preclinical efficacy data are in line with reported preclinical data on other clinical vaccine candidates aimed at prevention of disease in adolescents and adults[43,44], although caution is needed when comparing to historical data.

5. Beyond these specific issues, the manuscript would benefit from deeper mechanistic insight. For a study submitted to Nature Immunology, the current analysis is heavily phenotypic, relying on enumeration rather than mechanistic dissection. The authors touch on the potential differences between unmodified and modified mRNA platforms but do not explore early innate responses, antigen presentation dynamics, or tissue-resident memory formation, all of which are central to understanding TB vaccine efficacy. Likewise, the presence of $CD8^+$ memory precursor phenotypes in lung tissue is intriguing, but without functional data—such as depletion or blocking studies—it is difficult to ascertain their causal role in protection.

The reviewer touches on topics that are of course of great interest on scientific and vaccine development levels, such as early innate signatures. Indeed, a wide array of platform data on such responses is already available in the literature. Since such responses are predominantly antigen-independent, those platform data are likely applicable to our candidates. In parallel, these available platform data show that animal models are of limited predictive value to the human setting, because of inter-species differences in innate signaling. Conversely, *in vitro* models with human materials do not cover the complexity of prime-boost regimens and complex cellular composition in tissues. Therefore, such responses will be analyzed in detail in the ongoing clinical trials (NCT05537038; NCT05547464). Reviewer 3 (Comment 6) also requested more

discussion on the platform differences and expected translation towards humans. We have therefore provided more detailed discussion and references on these aspects in the manuscript. The changes are described in detail in the response to reviewer 3, comment 6.

On the mechanistic studies of adaptive responses, we strongly agree with the interpretation of the reviewer that the correlations we find do not establish a causal role. Depletion studies of these populations would perhaps be of scientific interest in understanding how these vaccine candidates function in murine tuberculosis. However, as discussed above, these models are of limited predictive value for human tuberculosis. Therefore, such data are unlikely to be of translational value, without first obtaining human immunogenicity that can be used as a reverse translation framework.

We have reflected these considerations by adding the following text to the discussion:

It remains to be established whether the BNT164-induced CD8$^+$ T cells causally contribute to TB protection. Further investigation of the direct contribution of specific cellular or humoral responses in mice, by for instance more comprehensive immune profiling, cellular depletion, or adoptive transfer studies, would be of scientific interest. However, these may be of limited translational value until BNT164-induced immune responses are characterized in humans, providing a benchmark for future investigation.

2. Reviewer #2 (Remarks to the Author)

In this manuscript, Agrawal et al evaluate safety profiles, immunogenicity, and efficacy of BNT164a1 and BNT164b1, two mRNA-LNP-based TB vaccine candidates, in preclinical rodent models. Each vaccine construct expresses 8 Mtb antigens that are expressed across different infection states. The two formulations (a1 and b1) represent differences in mRNA chemistry: nucleoside-unmodified RNA (uRNA, a1) and N1-methyl pseudouridine-containing modified RNA (modRNA, b1). The authors report that BNT164a1/b1 are immunogenic, well tolerated and efficacious in these preclinical studies in rodents. BNT164a1 and BNT164b1 represent promising TB vaccine candidates, and these preclinical studies represent an important step in providing rationale for continued development and testing of these candidates in clinical trials. A phase Ia trial the safety, reactogenicity, and immunogenicity of BNT164a1 and BNT164b1 was conducted non-BCG-vaccinated, Mtb-uninfected healthy adults in Germany (completed in 2025). A phase 1b/2a trial of the same candidates is currently ongoing in BCG-vaccinated, IGRA+ and IGRA- adults in Mozambique and South Africa (estimated completion in 2026). The manuscript is clearly presented and well written, with thorough reporting of the methods. However, there are limitations of the study that impact interpretation of the data as noted in the comments below.

Comments:

1. Figure 2 represents a schematic for immunogenicity studies. Were prior dose escalation experiments conducted that resulted in selection of 4ug dose of BNT164a1/b1? Do the authors have prior data as well to demonstrate that 2 doses of BNT164a1/b1 is more optimal than a single dose? If dosing strategy is based on prior studies of mRNA-based vaccines generated by BioNtech, this may be helpful to mention in the text.

We thank the reviewer for their kind general words on our work and their fair assessment thereof. Regarding this comment, the reviewer is correct that this was based on the aggregate experience within our vaccine development pipeline. As suggested, **this is now mentioned in the results section** where we introduce the immunization schedule, as follows:
The dosing regimen was based on internal experience with mRNA vaccines, such as described previously[16].

2. Although vaccine-specific IgG responses were measured at 3 time points (d14, d28, d42), T cell immunogenicity is reported only at day 42. Data on the kinetics of T cell responses (peak immunogenicity time point) and durability and persistence of vaccine-induced T cell responses is lacking.

Indeed, T-cell responses were only assessed at times of sacrifice. The reason for this is that the read-out of antigen specific T-cell responses of eight different antigens requires relatively large cell numbers. Ethical guidelines of maximum blood draw volume therefore do not allow meaningful analysis of functional T-cell responses from blood. Especially since this material is also required to generate serum for ELISA read-outs. However, we have added the additional dataset of splenocyte responses at Day 155 as described in our response to question 3 below.

3. Figure 3: A third dose of BNT164a1/b1 was administered on day 134, which boosted IgG responses to RfpA, M72, HbhA, and VapB47. It is unclear why vaccine-induced T cell responses were not also evaluated at later time points and/or after administration of a 3rd vaccine dose.

We thank the reviewer for this comment. Splenocyte responses at the time of sacrifice were indeed obtained, but initially not presented. We have now added these data to the manuscript as Fig. S7 and have integrated a description of these data into the manuscript **(in the Durability and boostability Results section):**

Similarly, splenic T-cell responses three weeks after the third BNT164a1/b1 dose (Fig. S7) were comparable to, or higher than, those obtained three weeks after the second dose (Fig. S3). The greatest increases in splenocyte response were observed to Ag85A, ESAT-6, RpfD, RpfA and M72 following the three-dose schedule.

4. Lines 201-203: muscle fiber damage and inflammatory reaction related to BNT164 administration are noted as "data not shown". It would be useful to include these data in Supplementary Figure 5.

We thank the reviewer for this suggestion and have added corresponding histopathology images in the manuscript as Fig. S9. The text in the Results section was adapted accordingly.

5. BNT164a1/b1-induced T cell immunogenicity studies were conducted in splenocytes only. For TB vaccine candidates, it would also be important to demonstrate the presence of Ag-specific T cells in the lung (there are limited data from tetramer staining conducted in the lung post-Mtb challenge, but otherwise no immunogenicity studies of T cells in the lung after BNT164a1/b1 immunization).

In response to comment 9 of this reviewer, as well as comments by reviewer 1, we have now added two figure panels on intracellular cytokine staining of lung lymphocytes after peptide pool stimulation (Fig. S12 G-H). Because of the envisaged mode-of-action and target product profile of the BNT164 vaccine candidates, we have purposely not investigated mucosal immune responses outside of the infection context. The aim of the candidates is to induce systemic adaptive responses after intramuscular vaccination. Our immune phenotyping data in the challenge context were primarily aimed at investigating whether the induced lymphocytes have the capacity to migrate towards the site of infection. This was confirmed by the tetramer staining data and is now bolstered by the addition of the intracellular cytokine staining data.

A brief reflection on this subject was added to the discussion:
Observed lymphocyte infiltration in the lung was driven by antigen-specific $CD8^+$ T cells, indicating that systemically induced T cells have the capacity to migrate to the site of infection. Intracellular cytokine staining, after stimulation with antigens' peptide pools, confirmed these lung $CD8^+$ cells are functional cytokine-secreting cells.

6. No ESAT6- and RpfD-specific IgG responses were induced in any of the 3 mouse strains by either vaccine construct. Can the authors comment on why these antigens are not inducing IgG antibodies? Did the investigators evaluate induction of other Ab isotypes?

We thank the reviewer for addressing this important point, which was also raised by the other two reviewers. We have now included an additional dataset addressing antigen immune interference as Fig. S6, which shows that combining the individual ESAT-6-RpfD cassette with other three mRNA cassettes in BNT164, leads to loss of ESAT-6 antibodies. As for RpfD, we suspected the lack of antibodies to be a consequence of the predicted transmembrane domain in this protein. Efforts to optimize the antigen cassette by modifying or deleting this transmembrane domain did not markedly improve antibody responses, while strongly impairing the induced T-cell responses to RpfD, as this domain contains highly immunogenic T-cell epitopes. Since ESAT-6 and RpfD were included as T-cell targets, the lack of antibody responses was considered acceptable in the context of an octavalent vaccine candidate. Induction of other antibody isotypes was not evaluated. **The manuscript was edited to describe the newly added dataset as described in our response to Reviewer 1, comment 2.**

7. Figure 5B, C: were statistical comparisons performed for CFUs between BCG, BNT164a1, and BNT164b1-immunized mice post-challenge? CFUs for BNT164a1/b1-immunized mice appear to be higher in the lung and spleen 30 days post-challenge, compared with BCG-immunized mice. The results section describes comparison of CFU between BNT164a1/b1-immunized and saline controls only, but it would be important to demonstrate protection of BNT164a1/b1 relative to BCG, the only currently licensed TB vaccine.

We thank the reviewer for this comment, which is related to comment 4 of reviewer 1. Statistical comparisons between BCG and BNT164 candidates were purposefully not performed. The research question of our experiments was whether BNT164a1/b1 had the potential to protect mice against Mtb infection. BCG was included in these studies as an essential internal benchmark and positive control of the assay. Even though the reviewer rightfully states that BCG is the only licensed vaccine against TB, it is important to discern that the efficacy of BCG is predominantly in neonates and young children. The BNT164 candidates were designed as a prevention of disease (POD) vaccine for adults and adolescents where BCG has little to no proven efficacy. A more relevant comparison would be with other experimental vaccine candidates targeting POD in adolescents and adults. We have added a section to the discussion describing these considerations, which were not sufficiently described in the original manuscript. Furthermore, we have added the reference to the excellent recent work by Pozo-Ramos & Kupz 2025, Front. Immunol. which systematically compares preclinical results to clinical TB vaccine candidates over the past decades. **Changes made in the Discussion are provided in our response to Reviewer 1, comment 4.**

8. There does not seem to be a group of animals that is primed with BCG, then

immunized with BNT164a1/b1, which would more closely represent real-world applications of this vaccine in which much of the population at risk for TB reside in countries in which BCG is routinely administered. Since BNT164a1/b1 immunization alone does not seem to be more protective than BCG (by CFU data in Fig 5), it would be interesting to see if BCG prime + boosting with BNT164a1/b1 is more protective than either BCG prime alone or BNT164a1/b1 prime/boost alone.

Similar to the comment above, we strongly agree with the reviewer on the importance of this scientific question: how BCG, Mtb, or other mycobacterial pre-exposure would impact immunogenicity of the vaccine candidates. However, we believe that the proposed murine model would not give the scientific answers we are striving for. A BCG prime/BNT164 boost study would not be comparable with a situation in humans who are vaccinated with BCG early in life and would receive BNT164 candidates decades later, where BCG protection has likely waned. However, we would like to emphasize that this question will be addressed in the ongoing clinical trials. As the reviewer mentioned, the BNT164-01 trial in Germany includes individuals with minimal pre-exposure from BCG vaccination or mycobacterial infection, while the BNT164-02 trial in South Africa and Mozambique is further stratified into Interferon Gamma Release Assay negative and positive populations. The datasets obtained from these three populations will form a very exciting source to provide answers to the question posed.

This has already been mentioned in the discussion:

Further BNT164 immunogenicity and efficacy testing, in naïve and Mycobacterial antigen-exposed individuals, therefore remains to be determined in clinical trials.

9. Evaluation of Ag-specific T cell responses in the lungs and mLNs of mice post-challenge is restricted to evaluation of frequency of CD8 T cells staining with one M72-specific tetramer and CD4 T cells staining with one ESAT-6 tetramer. Given the 8 antigens encoded by BNT164a1/b1, it remains unclear what the overall frequency of Ag-specific CD4 and CD8 T cells is in the lungs and lymph nodes of BNT164a1/b1-immunized mice. It is not clear why the investigators did not also conduct intracellular cytokine staining assays to get a more complete measurement of Ag-specific T cells in the lungs/LN.

Due to the limited availability of biological samples from this experiment, CFU counting, pathology and the tetramer and T-cell phenotype panel were prioritized. However, our experiment did include a modest intracellular cytokine staining read-out, which we had initially omitted to streamline the manuscript. However, reading the comments from both reviewer one and two, it became clear that these data address an important shared concern, and we have added them to the revised manuscript as Fig. S12 G-H, accompanied by the **following text in the results section**:

Functionality of $CD8^+$ lung lymphocytes was confirmed by intracellular staining of IFNγ and TNFα after stimulation with two peptide pools of similar amino acid content, encompassing BNT164 antigens. One pool included peptides from M72, VapB47 and Ag85, and the other included peptides from Hrp1, ESAT-6, RpfD, HbhA and RpfA. Stimulation with either pool induced significantly higher numbers of $IFN\gamma^+/TNF\alpha^+$ double-positive cells compared with the control (Fig. S12 G-H), confirming that BNT164-induced antigen-specific $CD8^+$ T cells are functional.

10. Figure 6 and Supplementary Figure 7: tetramer staining of Ag-specific T cells in the lung is depicted as % of CD8 T cells. The data should also be presented as absolute number of tetramer+ cells in the lung.

As requested by the reviewer, we have added the absolute numbers of these cell populations to Fig. S12 (panels D-F).

11. Supplementary Figure 7: ESAT6 tetramer+ CD4 T cells are low frequency and not a clearly defined population. It would be useful to show ESAT6 tetramer staining of lung CD4 T cells from an uninfected mouse to better evaluate background/non-specific binding of this tetramer.

We agree in principle that inclusion of a non-infected cohort of animals would have been a nice resource for certain immunogenicity read-outs. However, we would like to emphasize that the ESAT-6_{1-20} tetramer employed in our study has been used extensively within the field for over two decades (e.g. Winslow *et al*. 2003, *J. Immunol*). The frequency of ESAT-6_{1-20} positive cells in the lungs (1-5%) and lymph nodes (0.1-1%) of unvaccinated infected animals, are fully congruent with in-house historical comparisons and those available in the literature (e.g. Basile et al. 2020, Frontiers in Immunology). In light of this, we did not consider it ethical or otherwise pertinent to include additional mice in these studies as negative controls. This would of course have been essential if less established reagents had been employed.

12. Conclusions regarding elicitation of lung-resident CD8 T cells by BNT164a1/b1 and interpretation of these cells as potential correlates of protection should be interpreted with caution, given the limited approaches used to evaluate Ag-specific T cells (which are restricted to 1 CD8 tetramer and 1 CD4 tetramer that did not demonstrate evidence of clear staining). Measurement CD4 and CD8 T cells in the lung that are specific for the 8 antigens in BNT164a1/b1 could have been more comprehensively evaluated by intracellular cytokine staining.

We thank the reviewer for this multifaceted comment. Firstly, we agree that caution is warranted in the interpretation of our data. Although these responses were correlated with protection in our experiments, this does not mean that such responses can be seen as bona fide correlates of protection. That terminology should be reserved for correlates with clinical relevance, which our murine data cannot provide.

To emphasize this point we have added the following sentence to the discussion: *It remains to be established whether the BNT164-induced CD8$^+$ T cells causally contribute to TB protection.*

Furthermore, to avoid the risk of being too speculative on this matter, in the same section we have deleted the sentence: *[… cytokine-producing CD8$^+$ T cells], for which our data suggest a potentially beneficial role in protection.*

To address the second part of the comment we have now included intracellular cytokine staining data as detailed in the response to comment 9 from the same reviewer.

13. Lines 278-287: Supplementary Figure 7 is repeatedly referred to as Fig S77.

We thank the reviewer for making us aware of this error. This has been corrected.

3. Reviewer #3 (Remarks to the Author):

Review for "A Multi-Antigen mRNA-LNP Vaccine Elicits T-Cell Immunity and Protection Against Mycobacterium tuberculosis in Preclinical Models" by Agrawal et. Tuberculosis remains the leading infectious disease cause of death worldwide. The currently available approved vaccine against TB provides protection against TB in newborns, but limited to protection to adolescents or adults, that harbor the majority of disease and drive transmission. The current manuscript addresses this gap, and describes a tour de force study focused on the development of an Mtb mRNA/LNP vaccine consisting of key Mtb antigens able to induce protection against Mtb in rodents.

The COVID-19 pandemic highlighted the potency and flexibility of the mRNA/LNP vaccine platform, able to induce robust Th1 immunity. Given the flexibility and multivalent capacity of mRNA, the authors selected an 8 antigen cocktail, representing key antigens that have been shown to provide protection historically using a CMV-vectored vaccine, as well as including 2 additional antigens, one that was recently shown to provide protection in a human Phase2b trial (M72) and one that was shown to provide protection against dissemination (HBHA). The authors designed 4 fusion proteins, encoded on 4 mRNAs, using either modified (BNT164b1) or unmodified (BTN164a1) mRNA, that produced antigens in vitro, all showed comparable T and B cell

immunogenicity in rodent models, safety in a tox rat model, and afforded protection against challenge with 2 Mtb strains, in the setting of infiltrating T cells into lungs.

The major advances include:
-The study includes a multi-antigen design, covering antigens expressed at key steps of bacterial infection selected based on their previous capacity to provide protection against Mtb, all of which were screened rigorously and expressed in vitro and showed some level of both B and T cell immunogenicity in vivo. These are absolutely critical data as the expression of bacterial antigens in a mammalian cell in a manner that can induce conformationally correct immunity (based on antibody binding to Mtb lysates and recombinant antigens) is a major step forward for the mRNA vaccine world.
-The study highlights the induction of robust CD8 T cell responses, that have not been induced readily by other vaccine platforms that are currently in testing across the globe, but that have been linked to the control and elimination of Mtb, immunity that is currently not elicited by other Mtb vaccines that are in preclinical or clinical development
-Given the emerging appreciation for the potential role for antibodies in Mtb control, the study also demonstrates the induction of humoral immune responses to both individual antigens but also to Mtb lysates, including durability and boostability data for nearly all tested antigens, that is likely to be absolutely essential for long-lived protection for decades of life in highly endemic areas.
-The study explores protection not just against the commonly used lab strain of Mtb (H37Rv), but also a more virulent Mtb strain (HN878).
- The study provides interesting first insights on correlates of immunity in the lungs by tissue immune-histochemistry and flow cytometry demonstrating the infiltration of CD8+ T cells in the lungs of mRNA immunized animals.

This incredible body of work provides robust, mechanistic evidence to support the development of this vaccine in humans, marking the development of an exciting new strategy to combat Mtb via the potential induction of lung-trafficking T cells, long-lived humoral immunity, and robust boostability, that may be key to protection against Mtb in adolescents, most vulnerable to the disease. With human testing already underway, these data will be of great interest to the TB field, moving the entire field closer to a transmissible-disease-blocking vaccine. However, there are a few comments below that could improve the manuscript:

1. The authors describe the rationale for the selection of the 8 antigens and provide a cartoon of the designs. However, more detail on the precise arrangement and linkers used to generate these fusions, and the arrangement of each of the genes could be very helpful and provide completeness for the paper. Alpha-fold projections of the fusion

constructs and single proteins can help readers visualize the likely preservation of antigenicity. Some rationale for why particular sets was fused could also be helpful.

We thank the reviewer for their kind words. We had described the RNA design in the respective Methods section, specifying the signal peptide used and stating that no linkers were used to generate fusions. **We have now supplemented this section with additional details including the construct sizes and the rationale for pairing:**

This resulted in four fusion proteins of different amino acid sizes to allow the possibility of size-based RNA analytics: Ag85A-Hrp1 (465AA), ESAT-6-RpfD (273AA), RpfA-HbhA (630 AA), and M72-VapB47 (847AA).

Importantly, we have now also added the requested structural models as Fig. S1 to the results section for antigen design. This new figure depicts the Alphafold models of fusion proteins overlapped with determined or predicted structures of the single antigens. The models show a high structural similarity of antigens where data is available. Lower structural similarity in antigens where only predicted models are available, could be attributed to regions of low prediction confidence.

The following text has been added to the results section to describe these data:
To better understand how the BNT164 antigen design strategy could impact protein conformation and antigenicity, we generated structure predictions of the four chimeric fusion antigens with AlphaFold2 and aligned them to protein structures of single antigens (Fig. S1). For antigens where experimental structures were available (Ag85A, Hrp1, ESAT-6, and the PPE-domain within M72), there was a high level of overlap between the chimeric fusion protein models and structures of single antigens, with calculated root mean square deviation (RMSD) values of <1 Å. RMSD values were higher, indicating poor alignment, for antigens and domains lacking experimental structures and for regions predicted to be unstructured. Regions of lower structural overlap between single and fusion antigens were especially concentrated in domains with low model confidence as indicated by local distance difference testing (LDDT, Fig. S1). These predictions suggest that the chimeric antigens of BNT164 have the potential to be folded correctly and can access native protein conformations capable of inducing relevant immune responses.

Corresponding methodology, references and a sentence in the discussion have also been added to the revised manuscript.

2. *Interference across antigens has been observed with multiplexing. While the authors show the expression of individual proteins in vitro and immunogenicity in vivo, it would be helpful for the authors to include data on single immunization efforts to understand the level – if any – of interference that was observed during the design stage.*

We thank the reviewer for raising this important matter, which is closely related to some of the concerns raised by reviewer 1 (comments 1 and 2). In our initial submission we did not provide much detail on the design consideration of the BNT164 candidates. However, the potential for antigen interference was investigated in detail. To provide more detail on this topic we have added a full new supplemental figure describing an important immunogenicity study in this context. **Accompanying text was added to the Results section, as stated in our response to Reviewer 1, comment 2.**

3. *Given the paucity of reagents to test for correct conformation/antigenicity, the authors used a tag to measure protein expression. A monoclonal antibody was used to test the antigenicity of Ag85a. Given that Mtb exposed individuals are also likely exposed to these antigens, it would be quite interesting to see whether mRNA encoded antigens can be recognized by plasma samples from individuals naturally exposed to Mtb as a translational datapoint.*

The reviewer is correct about the technical challenge of reagent scarcity in the tuberculosis field. However, we would like to clarify that no tags were used to measure protein expression throughout the study. Rather, for BNT164 antigens for which commercial antibodies were not available, we developed custom-made antibodies through mouse immunization with recombinant proteins. Monoclonal antibodies were then isolated using hybridoma technology and used in the western blots presented in Figure 1B-E. We have adapted the methodology section to emphasize the use of antigen-specific antibodies and provide additional information on how the antibodies were raised.

The following text was added:

Custom-made primary antibodies were raised in BALB/c mice following repeated SC immunization with individual recombinant protein mixed with Freund's incomplete adjuvant, and isolated using hybridoma technology.

4. The authors show T and B cell immunity as individual datapoints from the same, mouse studies, however whether B and T cell responses are induced in a coordinated manner is unclear. It would be helpful if the authors could include some correlational analyses to help the reader understand if antigens induced more coordinated T/B cell immune responses- or whether some antigens were strong B or T cell targets.

We thank the reviewer for this thoughtful question, which is highly related to question 5. Both matters are addressed together below.

5. Substantial variation was observed in T cell immune induction across the antigens. It

is unclear if this is related to differences in antigen expression. Was there any correlation between in vitro expression levels and likelihood of seeing a T cell immune response? Additionally, despite the variation across mouse strains, it is unclear whether these responses are correlated (ie. If the response is high in a B6 is it also high in an A2 or BALBc)? This knowledge would be incredibly helpful for the field and readers.

Thanks to the comments by this reviewer as well as reviewer 1, we realized a more thorough depiction and discussion of the data across models was warranted.

We have now added a novel figure (Fig. 2E) to the main text to address the question on T-cell responses across models. This figure depicts aggregated splenocyte responses against each of the antigens in the three different mouse models employed in the study. In multiple cases, an antigen without significant induction of T-cell responses in one model reached the upper limit of detection in the other models (e.g., Hrp1, HbhA, VapB47, RpfA). The colored visualization used in this graph should also help the reader to assess the responses by either individual antigen, or by fusion protein antigen cassettes. These data thereby also help to address whether these antigen responses are dependent on *in vivo* expression. For instance, antigens with limited T-cell immunogenicity in the C57BL/6 mice, such as Hrp1 and VapB47, are co-encoded as fusion proteins with other antigens which exhibited high T-cell immunogenicity (Ag85A and M72 respectively). These findings do not support the hypothesis that low *in vivo* expression drives the lower T-cell immunogenicity. Even stronger evidence to reject this hypothesis is that Hrp1 and VapB47 induced IgG titers in the same experiment. Taken together, our data also do not suggest that observed differences in T-cell responses were due to different *in vivo* expression levels, but rather point towards MHC/HLA differences across models.

The novel overview of splenocyte responses across models also helps to illustrate the correlation between B- and T-cell responses, or rather lack thereof. While IgG-responses to all antigens were highly similar between BALB/c (Fig S5A) and C57BL/6 mice (Fig 3A), splenocyte responses per antigen varied across models (Fig 2E).

The following text was added to the results section:

ELISpot analysis using total splenocytes reflected the trends obtained with purified CD4+ and CD8+ splenic T cells in all three mouse models, but showed overall higher responses (Fig. S3 and summary in Fig. 2E). Taken together, all BNT164-encoded antigens raised T-cell responses in at least two out of three tested mouse models, with observed differences likely being driven by MHC/HLA variations between those mouse models.

And these considerations are more thoroughly discussed in the Discussion, as described in our response to Reviewer 1, comment 1 (bullet point 2).

6. There is limited discussion on why limited differences were not observed across modified and unmodified mRNA that showed dramatic differences for COVID-19 vaccines in both humans and NHP. Some discussion should focus on this biology to help readers contextualize these results in the broader understanding of the use of these different mRNA types.

We thank the reviewer for this suggestion and have extended the discussion, including recent publications on the topic and an extra sentence on the hallmark paper by Tahtinen et al.

The section now reads:

Innate signaling pathways induced by mRNA-LNP are species-specific and remain to be fully elucidated[36]. For example, Tahtinen et al. recently showed that human immune cells respond to mRNA by producing inflammatory IL-1 cytokines, while mice produce markedly higher levels of anti-inflammatory IL-1ra[37]. Non-human primate studies can be more informative for comparing mRNA platforms[38], but even these higher order models do not fully recapitulate human signaling pathways.

7. Moreover, despite the limited expression and peak immunogenicity levels across the 2 mRNA types, BNT164b1 exhibited a 40-fold boost compared to BNT164a1 (8-fold). Do the authors believe this difference is related to the better priming by modified mRNA?

We thank the reviewer for this intriguing question, prompting us to reanalyze our available data. A technical replicate measuring lysate binding of the same samples, confirms the relatively higher values of BNT164b1 lysate-binding titers compared with BNT164a1. The relative platform differences in fold-change from Day 42 to Day 155 were slightly less pronounced in that replicate, with a 15.3-fold increase for BNT164a1, and 37.7-fold increase for BNT164b1. When comparing these data with recombinant protein ELISA we do not do not consider it appropriate to state that the observed difference in this experiment is a bona-fide platform difference. For example, data on M72 ELISA at Day 42 of another experiment (Figure 3A), suggest higher responses in the BNT164b1 candidate. Yet, M72 ELISA data from Day 42 of the long-term study in the same mouse model (Figure 3B) would suggest a trend slightly favoring BNT164a1.

Taken together, we cannot exclude that the minor changes between the fold-change increase of lysate-binding across the platforms is caused by experimental variation. Therefore, we would prefer not to overinterpret the apparent differences across those platforms and rather focus on the highly robust findings that the third BNT164 dose increased antibody binding to Mtb lysate irrespective of the mRNA platform used.

8. For infiltrates, the authors demonstrate the selective increase in CD8+ T cell populations, and mention limited CD4+ T cell infiltration. The inclusion of negative data can still be helpful to the field. Additionally, were other lymphocytes, including NK cells also observed, potentially important given the presence of vaccine induced antibodies that may have functional impact on limiting bacterial growth.

We thank the reviewer for emphasizing the importance for negative data and have added the corresponding plots on CD4 + T-cell infiltrates to what is now supplemental Figure 11. No data on NK cells or other lymphocytic populations were generated.

We have added the following text on the limitations of our study to the Discussion:

Further investigation of the direct contribution of specific cellular or humoral responses in mice, by for instance more comprehensive immune profiling, cellular depletion, or adoptive transfer studies, would be of scientific interest. However, these may be of limited translational value until BNT164-induced immune responses are characterized in humans, providing a benchmark for future investigation.

9. Finally, for the protection studies, it is unclear whether M72, currently in Phase3 trials, was tested alone, but could provide vital insights into the additional benefit of more antigens that may help provide enhanced protection against the bacteria.

We have not performed protection studies with the M72 antigen alone. The final antigen composition of BNT164a1 and BNT164b1 was determined based on expression and immunogenicity studies, and we confirmed that M72 remained highly immunogenic in the octavalent BNT164 candidates. We would like to emphasize that even if challenge data on the M72 antigen alone would be available, it would still be challenging to infer contribution of the other antigens based on additive value. Especially for Hrp1, VapB47, RpfA and RpfD, as they are not expected to be expressed in the used mouse model. As we now discussed more thoroughly in the new discussion section, this is reflected in our general antigen selection approach: Antigens included based on evidence from human immunogenicity and NHP-protection data, should not be excluded based on murine data in models of limited predictive value.

Response to reviewers

We would like to thank all three reviewers again for their help in improving our manuscript. We have addressed the remaining points raised by Reviewer #3 below.

Beyond those changes we have restructured the document according to the journal's editorial instructions including a separation of Extended data and Supplemental information items. We have generated figures depicting full western blot images and have updated figure 1 to improve the quality of the figure changed it to portrait format. And we have corrected a mistake, changing mesenteric to mediastinal lymph nodes.

We hope that the editor and reviewers agree with these changes and find our revised work suitable for publication.

Reviewer #3 (Remarks to the Author):

The authors responded skillfully across a diverse set of questions from all 3 reviewers. While the immunological data is very clear, it is still not clear to me precisely which mice mounted antibodies and/or T cells. A heat map of all the integrated data could be very helpful even if only as supplemental data.

To make the overview of all measured responses more visible we have generated the following heatmap which is added to the manuscript as Extended data figure 3. We thank the reviewer for the suggestion as we are sure that this figure is going to be of much use summarizing our immunogenicity results.

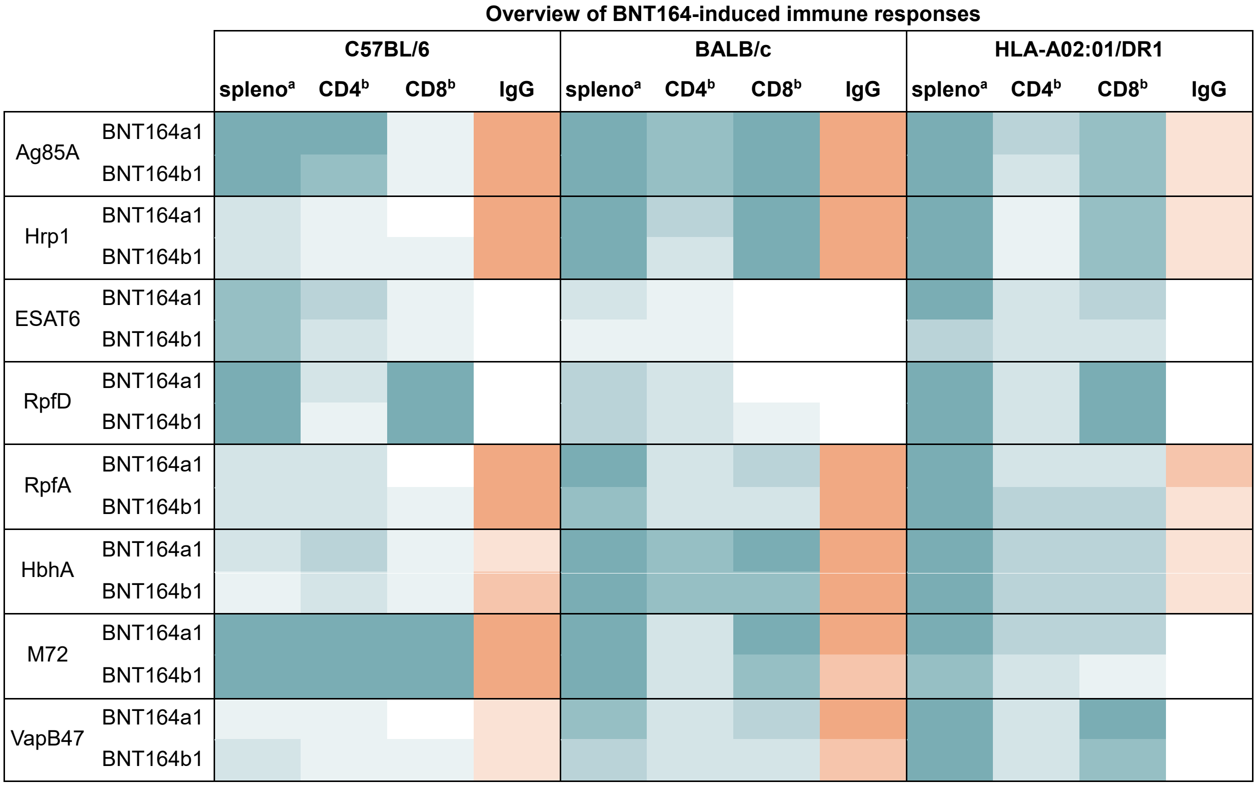

IFNγ SFU	<20	21-100	101-300	301-600	601-1000	>1000
IgG [μg/mL]	<1	1-10	10-100	>100		

Extended Data Figure 3. Overview of BNT164-induced immune responses

Heatmap summary of T-cell responses measured via IFNγ ELISpot, and IgG serum concentrations, measured via ELISA in three different mouse models following prime-boost immunization with BNT164a1 or BNT164b1. Data are presented in Supplementary Fig. 1, Fig. 2 C-D, Extended Data Fig. 2, Fig. 3A, and Extended Data Fig. 4.

[a] total splenocytes; SFU/5 x 10^5 cells. [b] purified splenic $CD4^+$ or $CD8^+$ T cells; SFU/ 1 x 10^5 cells.

The supplemental figure with the alpha-fold proteins is an important addition. I would suggest pulling this into figure 1 for transparency on the predicted constructs.

We thank the reviewer for their kind comment on the new figure. Unfortunately, it was not feasible to integrate the plots depicting these models within the current figure 1. However, in previous submissions we had not made the distinction between extended data figures and supplementary data figures. In preparing the final formatted manuscript we propose to make this figure Extended data figure 1, so that it is directly available in the online version without the need to download and scroll through a supplemental information document. We hope the editor and reviewer agree with this approach.

It will be important to add a statement about the failures of the BCG revaccination studies that showed BCG is not protective in humans despite it being a benchmark in mice - and further emphasizing that M72 was protective in humans but not in mice. This will help contextualize these data that are essential to derisk the transition to humans which will be most informative. It may also be important to move the BCG results in the challenge study to the right side of the graph - as it is a distracting comparison - but a necessary control.

We thank the reviewer for this finetuning of our statements and have added and expanded the corresponding section in the discussion to make these points more explicit. As suggested, we have emphasized the limitations of using BCG in these models and clearly state that BCG should not be used as a comparator. We initially followed the suggestion by the reviewer to place BCG on the right side of all graphs, yet found this draws the eye to this group even more. During this process we noticed that the bright pink color used for the BCG group is a major factor in inviting the unwanted comparison. Therefore, we have now changed the color coding of BCG throughout the manuscript to dark grey, to signal its relation to the other control groups. We feel that this achieves the goal set out by the reviewer efficiently and that they agree with this approach.

Finally, we followed the reviewer suggestion and added a discussion on comparison to preclinical data available for the M72 candidate including references to the original Skeiky et al. 2004 paper as well as the excellent recent historical overview of the field provided by Pozo-Ramos and Kupz 2025.

The section now reads:
"It is important to note that BCG was included in our studies as a positive control, but should not be used for efficacy comparisons. Despite BCG's efficacy in mouse models and human infants, BCG revaccination does not protect against TB disease in adolescents and adults[43]. Rather, achieving similar lung CFU reductions with BCG as previously reported[44], helps validate the fidelity of the performed experiment and allows

cautious historical comparisons to other vaccine candidates[44]. Our preclinical efficacy data for BNT164 align with findings for other vaccine candidates aimed at prevention of disease in adolescents and adults[44-46]. This includes the M72 antigen adjuvanted with AS02 or AS01B, which provided approximately 0.6 - 0.7 Log_{10} CFU reduction compared with saline or adjuvant-only controls in the same C57BL/6 challenge model[45]. No murine protection studies with the clinical candidate M72/AS01E, currently being evaluated in a phase III trial (NCT06062238), are publicly available to our knowledge[44]. "

It may be important to add a note in the discussion related to the use of mRNA vaccines to prevent COVID, Flu, and RSV - all respiratory viruses using intra-muscular immunization. Although the vaccines do not prime T cells within the lungs, the platform has proven to be quite effective against respiratory pathogens, demonstrating an ability to raise peripheral T cells that can effectively migrate and respond to infections in the lungs.

We thank the reviewer for this suggestion and have restructured the discussion to integrate these considerations with the limitations of our work regarding limited investigation of lung-resident immune responses. The section now reads:

"One limitation of our work is that lung-resident immunity was only investigated in the context of infection. Intramuscularly administered mRNA vaccines can confer protection against respiratory viral pathogens like SARS-CoV-2 or RSV [18,19,50]. While neutralizing antibodies are most relevant for protection against these viruses, lung-resident T-cell responses have also been detected, albeit to a limited extent and mostly at a lower magnitude compared to actual viral infection[51,52]. In our Mtb challenge study with HN878 we found evidence of BNT164-induced lymphocyte infiltration in the lung. This was driven by antigen-specific $CD8^+$ T cells, indicating that systemically induced T cells have the capacity to migrate to the site of infection. Higher numbers of lymphocytes infiltrating the granulomas correlated with lower bacterial burdens."
